# Supplementary material for: Deployment expectations of multi-gigatonne scale carbon removal could have adverse impacts on Asia’s energy-water-land nexus
Source: Nat Commun. 2024 Jul 27;15:6342. doi: 10.1038/s41467-024-50594-5 (PMC11283554; doi:10.1038/s41467-024-50594-5)
Supplement: Supplementary file 1 — Supplementary Information [file 41467_2024_50594_MOESM1_ESM.pdf]

Supplementary Information

Deployment expectations of multi-gigatonne scale carbon removal could have adverse impacts on Asia's energy-water-land nexus

Jeffrey Dankwa Ampah[1,2], Chao Jin[1*], Haifeng Liu[2*], Mingfa Yao[2*]

Sandylove Afrane[1], Humphrey Adun[3], Jay Fuhrman[4], David T. Ho[5,6], Haewon McJeon[7]

[1] School of Environmental Science and Engineering, Tianjin University, 300072 Tianjin, China

[2] State Key Laboratory of Engines, Tianjin University, 300072 Tianjin, China

[3] Operational Research Centre in Healthcare, Near East University, TRNC Mersin 10, 99138 Nicosia, Turkey

[4] Joint Global Change Research Institute, University of Maryland and Pacific Northwest National Laboratory, College Park, MD, USA

[5] Department of Oceanography, University of Hawaii at Mānoa, 1000 Pope Road, Honolulu, Hawaii 96822, USA

[6] [C]Worthy, Boulder, Colorado 80302, USA

[7] KAIST Graduate School of Green Growth & Sustainability, Daejeon 34141, Korea

*Corresponding authors: Haifeng Liu (haifengliu@tju.edu.cn), Chao Jin (jinchao@tju.edu.cn), Mingfa Yao (y_mingfa@tju.edu.cn)

Contents

Supplementary Tables 3
Supplementary Figures 7
Supplementary Discussion 1: Impact on primary and final energy demand, and abatement costs 32
Supplementary Discussion 2: Impact on positive and negative emissions, net zero timing, and pollutants 35
Supplementary Discussion 3: Impact on land, water, and fertilizer consumption 39
Supplementary Discussion 4: Validation 42
Supplementary Discussion 5: Sensitivity analysis 43
Supplementary Note 1: Estimating capital stock turnover 49
Supplementary Note 2: Land use change as a carbon dioxide removal method in GCAM 49
Supplementary Note 3: BECCS as a carbon dioxide removal method in GCAM 50
Supplementary Note 4: DACCS as a carbon dioxide removal method in GCAM 51
Supplementary Note 5: DORCS as a carbon dioxide removal method in GCAM 51
Supplementary Note 6: Biochar as a carbon dioxide removal method in GCAM 52
Supplementary Note 7: ERW as a carbon dioxide removal method in GCAM 53
Supplementary Note 8: GCAM's land module 54
Supplementary Note 9: GCAM's water module 57
Supplementary Note 10: GCAM's fertilizer module 59
Supplementary Note 11: Model parameterization 60
Supplementary Method 1: Global Change Assessment Model (GCAM) 70
Supplementary Method 2: Demand for energy services in GCAM 71
Supplementary Method 3: Economic choice function in GCAM 72
Supplementary Method 4: Total technology cost in GCAM 75
Supplementary Method 5: Renewable resource supply in GCAM 75
Supplementary References 75

Supplementary Tables

Supplementary Table 1 Impact of varying levels of CDR reliance on primary and final energy demand, and abatement costs

Indicator	REFERENCE	HIGH	MODERATE	LOW
**Share in total primary energy by 2050 (%)**				
Fossil fuel without CCS	80	33.3	8.4	5.4
Fossil fuel and biomass with CCS	0	38	21	15.7
Renewables	18.5	21.7	51.5	59
Nuclear	1.5	7	19.2	20
**Final energy consumption by 2050 (Exajoule (EJ))**				
Coal and natural gas	123.5	84	12	10
Refined liquids	105.6	92.6	49.4	47.9
Electricity and hydrogen	96	139.7	162.1	166.3
Biomass	15.5	10.7	2.3	1.6
**Marginal abatement cost of carbon (\$ per tCO_2)**				
Annual average carbon price from 2025-2050	0	248	831	1038

CCS: Carbon capture and storage

Supplementary Table 2 Impact of varying levels of CDR reliance on positive and negative emissions

Indicator	REFERENCE	HIGH	MODERATE	LOW
**Positive CO_2 emissions by 2050 Gigatonnes CO_2yr^{-1} ($GtCO_2yr^{-1}$)**				
Building	2.00	0.64	0.03	0.01
Industry	9.80	2.64	0.19	0.13
Electricity	12.2	1.45	0.06	0.02
Transport	4.19	3.12	0.55	0.36

Other energy transformation	1.19	0.13	0.02	0.07
**Positive non-CO_2 emissions by 2050 ($GtCO_2eyr^{-1}$)**				
Methane	5.3	3.3	2.5	2.4
Nitrous oxides	1.8	1	1.3	1.3
Fluorinated gases	1.7	0.7	0.7	0.7
**Negative CO_2 emissions by 2050 ($GtCO_2yr^{-1}$)**				
LUC[a]	0.02	0.33	0.45	0.54
BECCS	0	4.60	1.88	0
DACCS	0	4.20	0	0
Biochar	0	0.44	0	0
ERW	0	2.15	0	0
DORCS	0	~0	0	0

[a] Net negative emission from land use change (LUC); BECCS: bioenergy with carbon capture and storage, DACCS: direct air capture and carbon storage, DORCS: direct ocean removal and carbon storage, ERW: enhanced rock weathering

Supplementary Table 3 Cumulative pre-mature retirement of power plants in Asia between 2015 to 2050

Fuel	Capacity (Gigawatt (GW))			Cost (2015 billion USD)		
	HIGH	MODERATE	LOW	HIGH	MODERATE	LOW
Renewables [a]	2	1	1	11	5	7
Nuclear	0.3	1	0.5	1	1	1
Biomass	20	54	123	133	328	742
Biomass CCS	28	1	0	139	3	0
Coal	824	882	916	4259	4612	4864
Coal CCS	2	591	672	10	2519	2894
Fossil (other) [b]	275	379	389	344	485	517
Fossil CCS (other) [b]	16	262	309	29	407	521

a: Includes solar, wind, geothermal, and hydro; b: Includes oil and natural gas. CCS: carbon capture and storage. USD: United States Dollar

Supplementary Table 4 Cumulative newly installed power plants in Asia between 2015 to 2050

Fuel	Capacity (GW)			Cost (2015 billion USD)		
	High CDR	Moderate CDR	Low CDR	High CDR	Moderate CDR	Low CDR
Renewables [a]	6064	9299	9473	12117	21220	21614
Nuclear	1149	2136	2241	6297	11854	12475
Biomass	27	60	128	43	213	551
Biomass CCS	448	192	0	3350	1496	0
Coal	1050	1047	1043	672	660	644
Coal CCS	677	641	698	3941	3783	4145
Fossil (other) [b]	604	601	597	92	89	84
Fossil CCS (other) [b]	717	754	1053	1487	1592	2185

a: Includes solar, wind, geothermal, and hydro; b: Includes oil and natural gas. CCS: carbon capture and storage. USD: United States Dollar

Supplementary Table 5 Impact of varying levels of CDR reliance on land use and demand for water under the different scenarios

Indicator	REFERENCE	HIGH	MODERATE	LOW
**Land allocation by 2050 (million square kilometers (Mkm^2))**				
Bioenergy crops	0.25	1	0.97	1
Crops	4.68	4.20	4.10	4.03
Other agroLand (shrubs, grass, other arable, pasture)	16.29	16.08	16.29	16.32
**Average water consumption from 2025-2050 (cubic kilometers (km^3))**				
Bioelectricity CCS	0	2.74	1.67	0
CDR (DACCS)	0	3.35	0	0
Bioenergy crops	4.84	33	28.42	30.70
Electricity generation	53.58	54.70	59.44	62.38

CCS: carbon capture and storage. CDR: carbon dioxide removal. DACCS: direct air capture and carbon storage

Supplementary Table 6 Comparison of results here with existing mitigation pathways for Asia

Indicator	Existing literature	Current study		
	**Previous scenarios**	**High CDR**	**Moderate CDR**	**Low CDR**
2050 Sequestration ($GtCO_2yr^{-1}$)				
BECCS	0.03-5.30	4.6	1.9	0
Fossil CCS	0-10	7.1	2.0	2.4
DACCS	0	4.2	0	0
Afforestation	0.2-2.6	0.3	0.5	0.5
2050 Emissions ($GtCO_2yr^{-1}$)				
Energy and industry	-1.9 to 11.5	11	7.0	8.4
2050 Energy ($EJyr^{-1}$)				
Final energy	72-308	328	226	226
Primary energy	109-415	450	312	313
2050 Land (million hectares (million ha))				
Cropland	90-890	417	410	400
Forest	316-706	550	540	550
2050 Policy cost (US$2010 per tCO_2)				
Carbon price	97-14122	296	2355	3093
2050 Water (km^3)				
Total water consumption	96-2238	1399	1344	1336

BECCS: bioenergy with carbon capture and storage. CCS: carbon capture and storage. DACCS: direct air capture and carbon storage.

Supplementary Figures

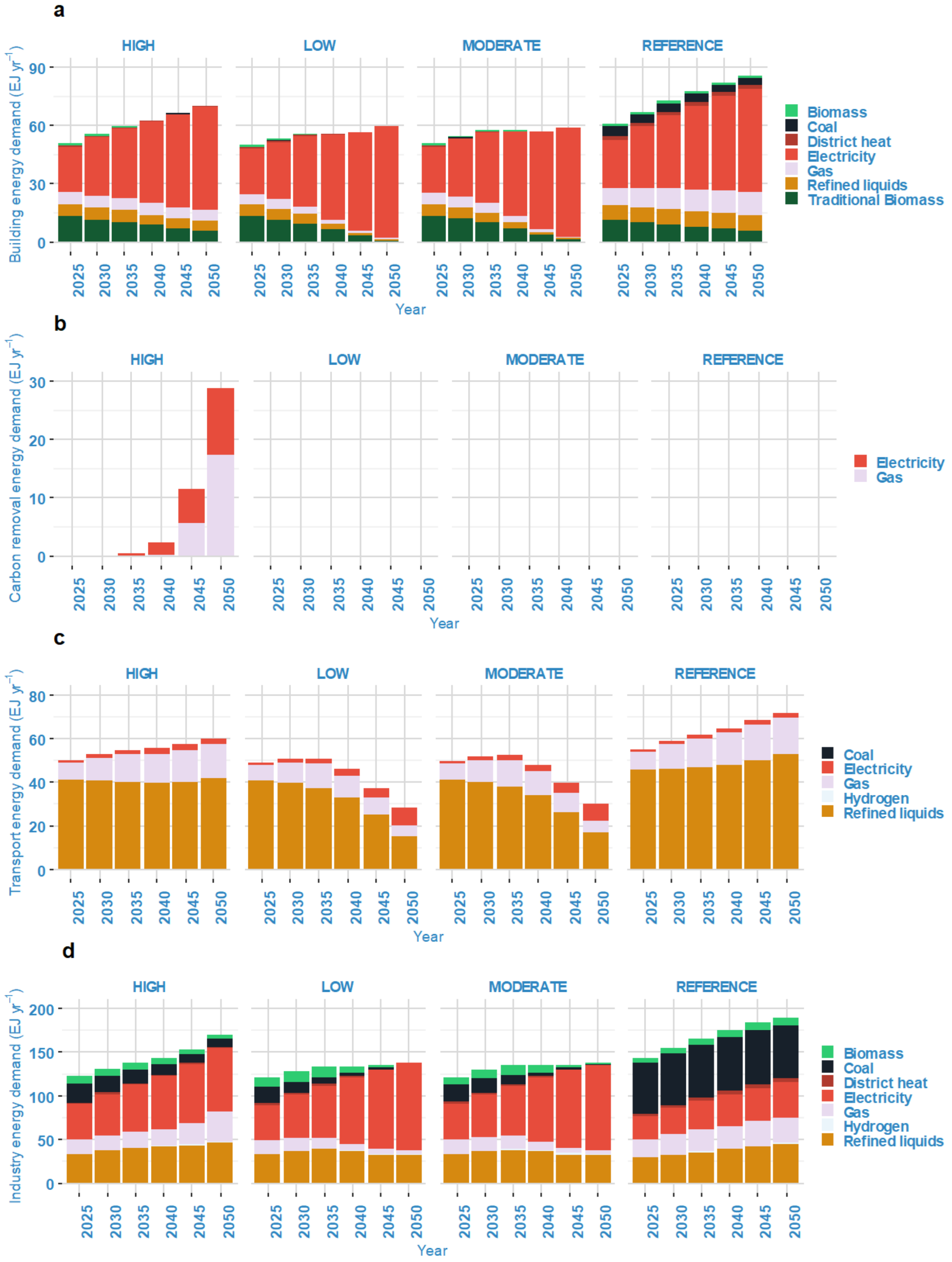

Supplementary Figure 1 Impact on final energy. Final energy consumption by fuel under varying CDR levels in building (a) CDR (b) transport (c) and industry (d) sectors. HIGH CDR pathway results in increased energy demands with relatively higher shares of fossil fuels across all sectors. Higher shares of electricity consumption in end-use sectors are recorded under LOW and MODERATE CDR due to the need for rapid emission cuts compared to the HIGH CDR scenario. Since LOW and MODERATE CDR scenarios do not rely on any energy-consuming carbon removal approach, there is no consumption of final energy for meeting demands of carbon removal. In all sectors except for transport, energy use increases with time. The situation is different for LOW and MODERATE CDR scenarios where total transport energy demands begin to decline towards mid-century. CDR: carbon dioxide removal; $EJyr^{-1}$: Exajoule per year

a

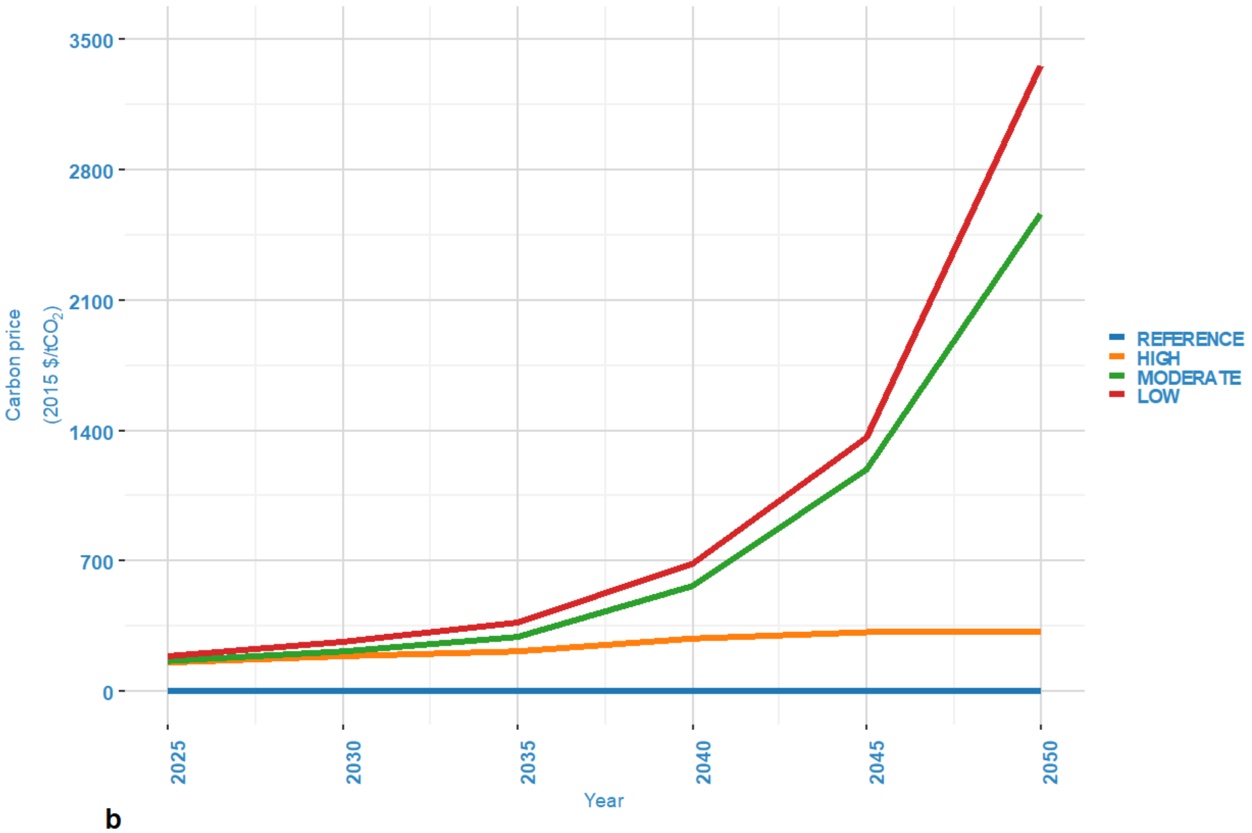
3500
2800
2100
1400
700
0
Carbon price
(2015 $/tCO$_2$)
2025
2030
2035
2040
2045
2050
Year
REFERENCE
HIGH
MODERATE
LOW

b

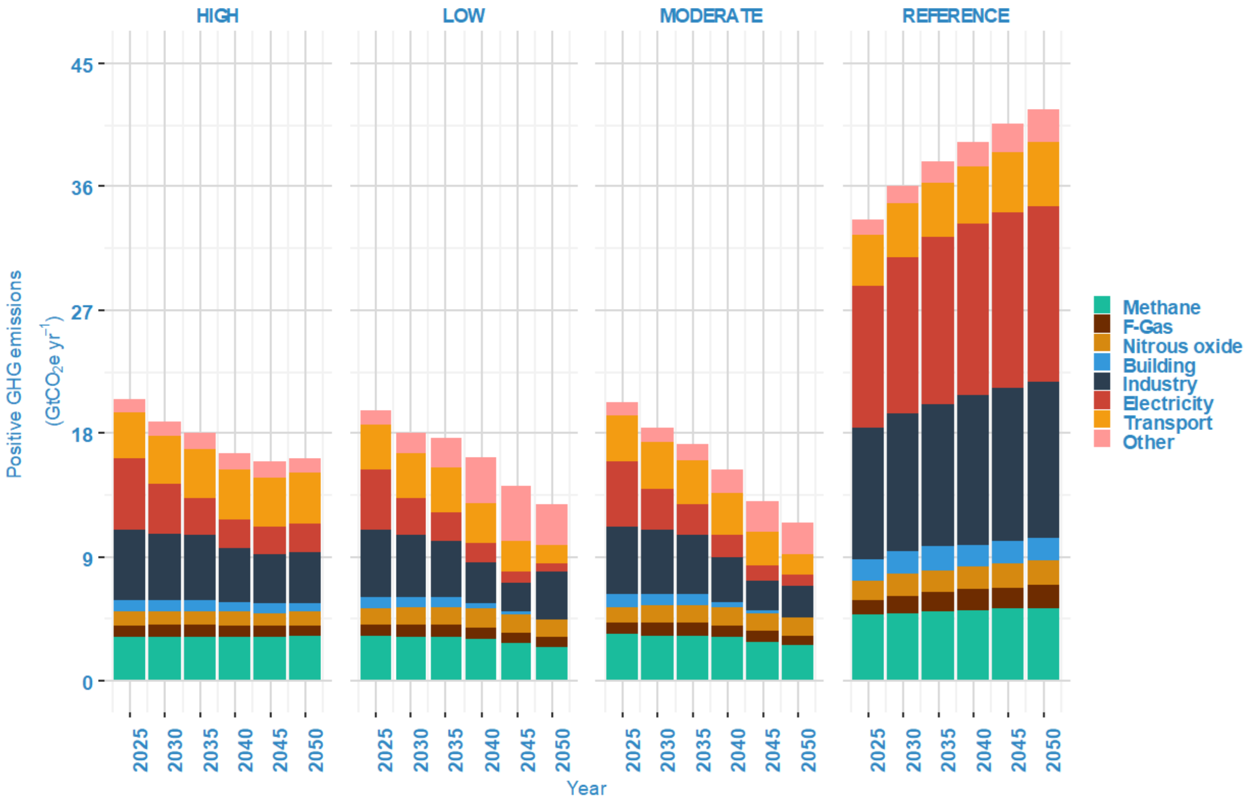
HIGH
LOW
MODERATE
REFERENCE
45
36
27
18
9
0
Positive GHG emissions
(GtCO$_2$e yr^{-1})
2025
2030
2035
2040
2045
2050
Year
Methane
F-Gas
Nitrous oxide
Building
Industry
Electricity
Transport
Other

Supplementary Figure 2 Impact on carbon price and emissions. Carbon price trajectory under varying levels of CDR reliance in Asia alongside a reference scenario (a). HIGH CDR pathway lowers the carbon price to achieve mid-century net zero target in Asia due to the higher reliance on fossil fuels and the ability to offset emissions later on. In contrast, rapid emission cuts are required under LOW and MODERATE CDR scenarios causing the price to reduce the last ton of emissions to increase significantly. In the absence of new climate change mitigation policies, carbon price stays zero. Total positive greenhouse gas (GHG) emissions by sector and species (Includes both fossil fuel and industry, and bio-derived CO_2 emissions) under varying levels of CDR alongside a reference scenario. F-Gas: fluorinated gases. Other refers to all other energy transformation processes such as refining and hydrogen production (b) The availability of CDR at multi-gigatonne scale (HIGH CDR) induces moral hazard significantly, slowing down fossil fuel phase out and causing higher residual GHG emissions. $/t$CO_2$: United States Dollar per ton of carbon dioxide; GtCO_2eyr^{-1}: gigatonnes of carbon dioxide equivalent per year

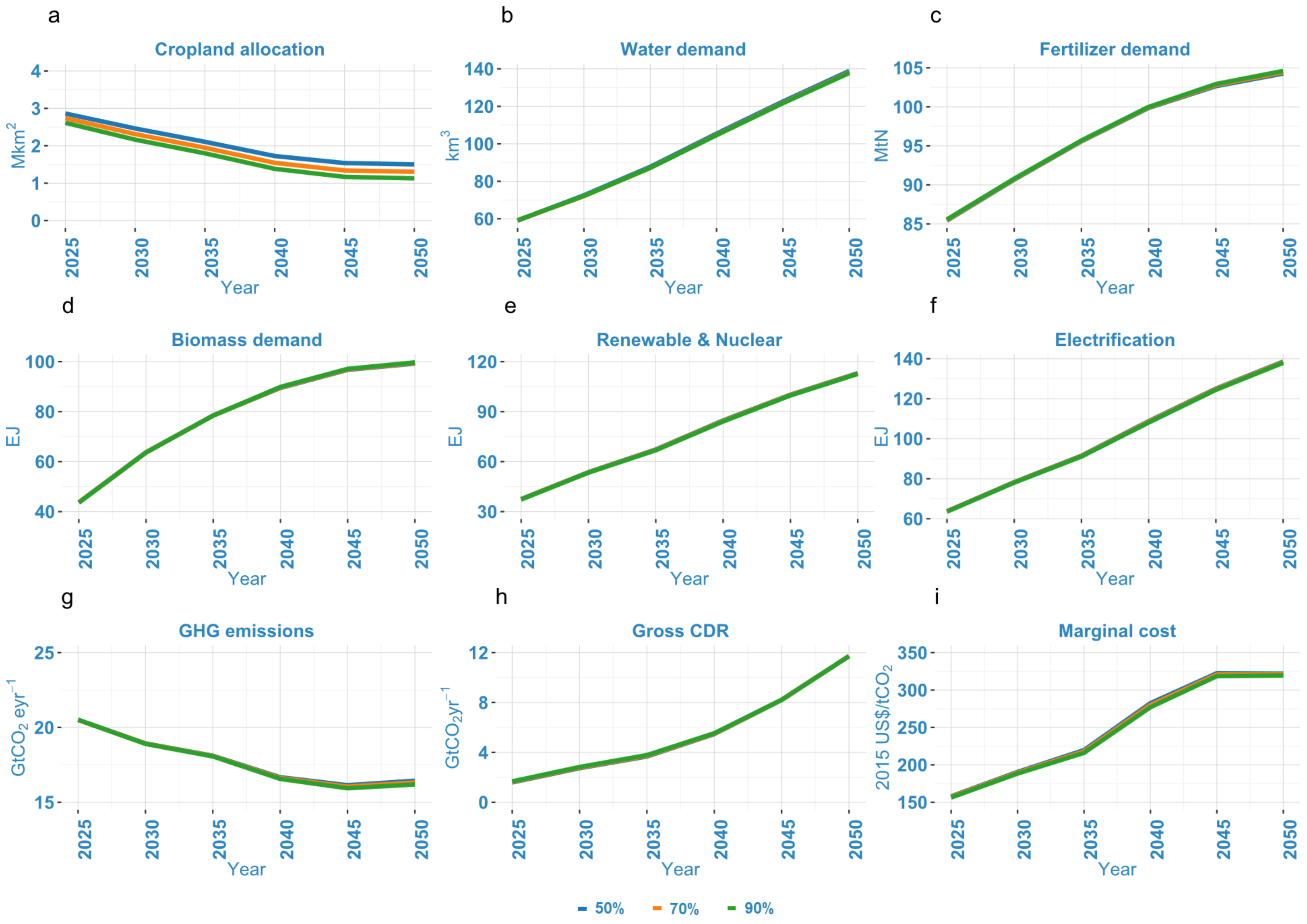

a
Cropland allocation
Mkm^2
b
Water demand
km^3
c
Fertilizer demand
MtN
d
Biomass demand
EJ
e
Renewable & Nuclear
EJ
f
Electrification
EJ
g
GHG emissions
$GtCO_2$ eyr^{-1}
h
Gross CDR
$GtCO_2yr^{-1}$
i
Marginal cost
2015 US$/$tCO_2$
Year
2025 2030 2035 2040 2045 2050
50%
70%
90%

Supplementary Figure 3 Influence of biochar sequestration rate. Impacts on cropland allocation without biochar (a) water demand (b) fertilizer demand (c) biomass demand (d) renewable and nuclear deployment (e) electricity consumption in end-use sectors (f) GHG emissions (g) gross CDR (h) carbon price (i). Since biochar, as a CDR approach, is only considered in our 'High' scenario, the sensitivity analysis was exclusively conducted on this particular scenario. Water demand represents water consumption for negative emissions and energy generation. $GtCO_2eyr^{-1}$*: gigatonnes of carbon dioxide equivalent per year; EJ: Exajoule;* $US\$/tCO_2$*: United States Dollars per ton of carbon dioxide;* Mkm^2*: million square kilometers;* km^3*: cubic kilometers; MtN: million tonnes of nitrogen; CDR: carbon dioxide removal; GHG: greenhouse gas*

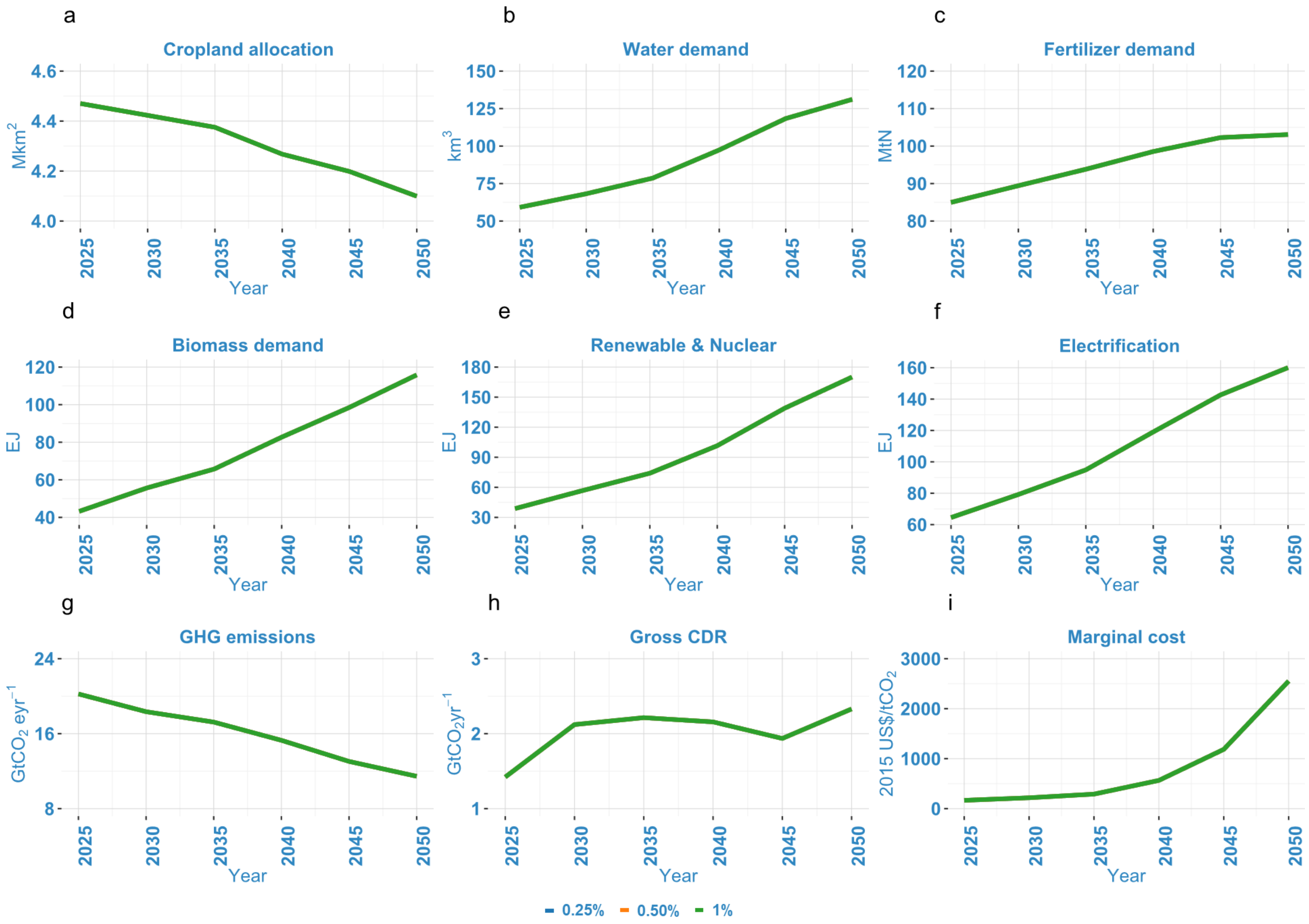

a
Cropland allocation
Mkm²
b
Water demand
km³
c
Fertilizer demand
MtN
d
Biomass demand
EJ
e
Renewable & Nuclear
EJ
f
Electrification
EJ
g
GHG emissions
GtCO2 eyr−1
h
Gross CDR
GtCO2yr−1
i
Marginal cost
2015 US$/tCO2
Year
0.25%
0.50%
1%

Supplementary Figure 4 Influence of negative emissions budget. Impacts on cropland allocation (a) water demand (b) fertilizer demand (c) biomass demand (d) renewable and nuclear deployment (e) electricity consumption in end-use sectors (f) GHG emissions (g) gross CDR (h) carbon price (i). Since biochar, as a CDR approach, is only considered in our 'High' scenario, the sensitivity analysis was exclusively conducted on this particular scenario. Water demand represents water consumption for negative emissions and energy generation. The sensitivity analysis here is based on our 'Moderate' scenario, since this scenario represents moderate challenges and regional impacts between decarbonization and carbon removal. Water demand represents water consumption for negative emissions and energy generation. While the negative emissions budget yields somewhat different results across scenarios, the differences between these scenarios are relatively negligible. Thus, the line charts representing these scenarios tend to overlap owing to their similarity in outcomes. $GtCO_2eyr^{-1}$: gigatonnes of carbon dioxide equivalent per year; EJ: Exajoule; US$/$tCO_2$: United States Dollars per ton of carbon dioxide; Mkm^2: million square kilometers; km^3: cubic kilometers; MtN: million tonnes of nitrogen; CDR: carbon dioxide removal; GHG: greenhouse gas

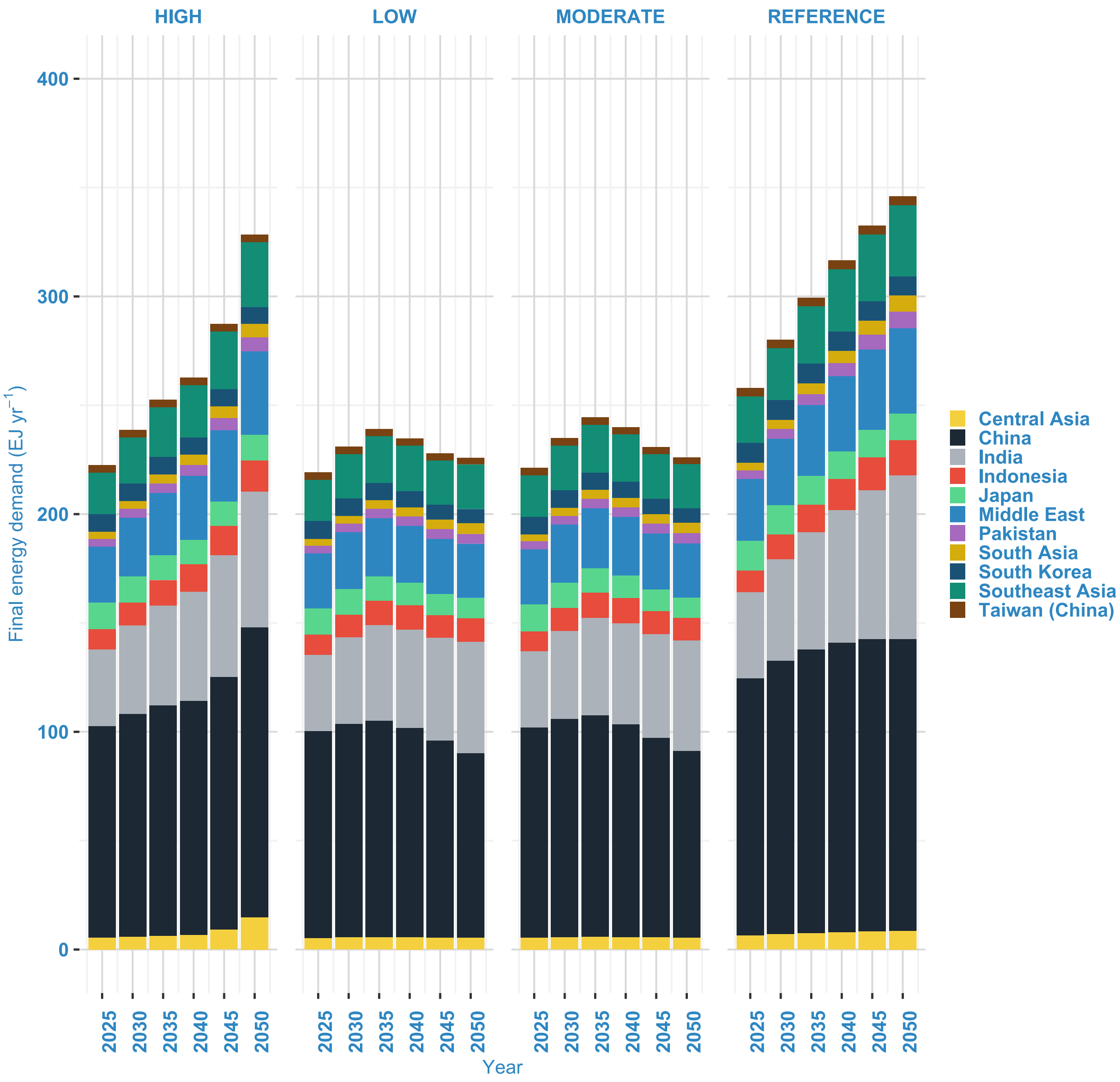

Supplementary Figure 5 *Impact on final energy demand. Final energy demand by individual Asian country/region under varying levels of carbon dioxide removal (CDR) alongside a reference scenario. HIGH CDR leads to more reliance on fossil fuels and less efficient use of energy, leading to relatively higher consumption of energy compared to LOW and MODERATE CDR scenarios. High population and gross domestic product (GDP) result in the highest share of final energy needs in China across all net zero scenarios and REFERENCE scenario. EJyr^{-1}: Exajoule per year*

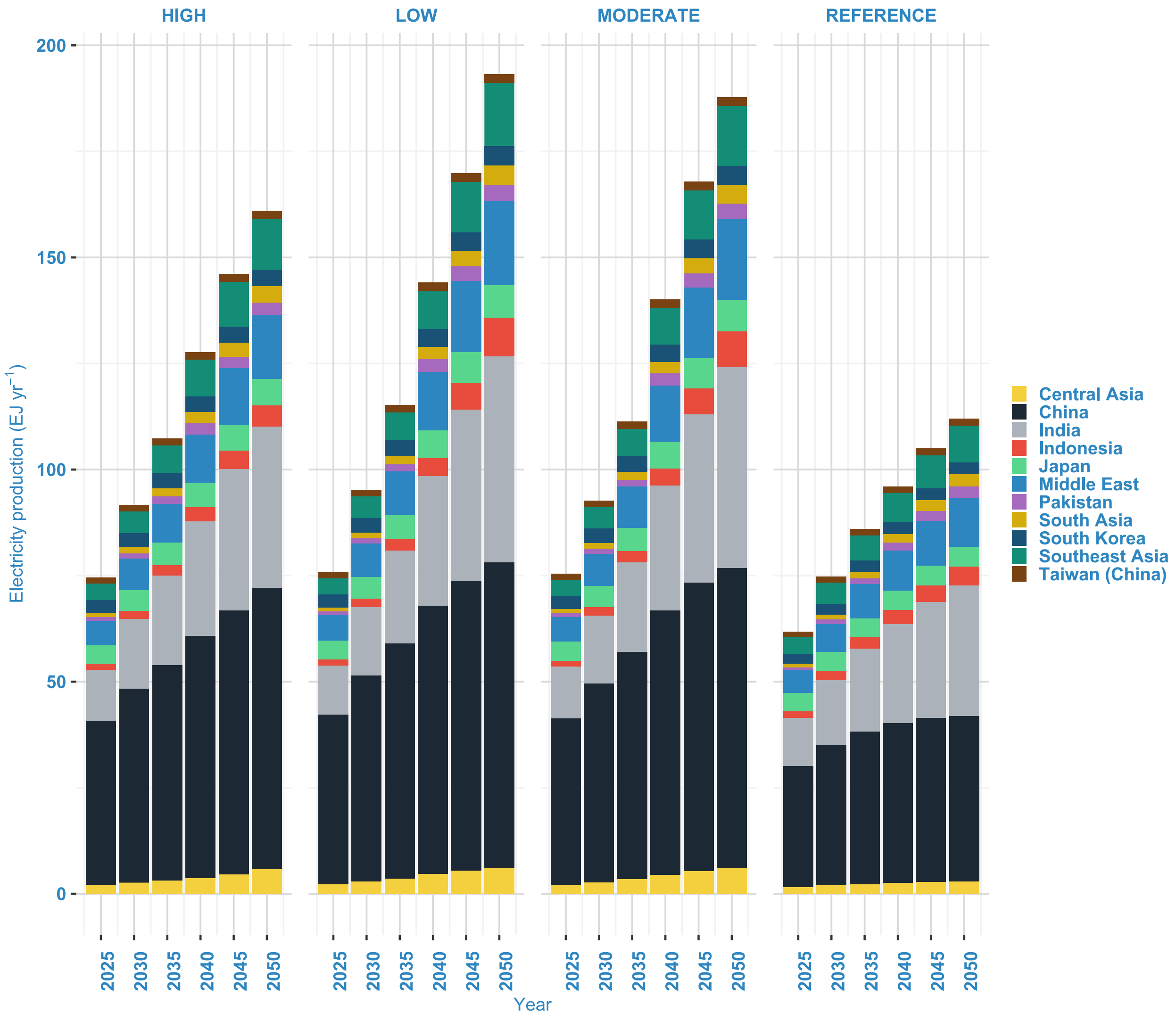

Supplementary Figure 6 Impact on electricity production. Electricity production by individual Asian country/region under varying levels of carbon dioxide removal (CDR) alongside a reference scenario. LOW and MODERATE CDR scenarios require rapid emission reduction in the absence of sufficient negative emissions leading to relatively higher electrification compared to HIGH CDR scenario. High population and gross domestic product (GDP) result in the highest share of electricity demand and production in China across all net zero scenarios and REFERENCE scenario. EJyr^{-1}: Exajoule per year

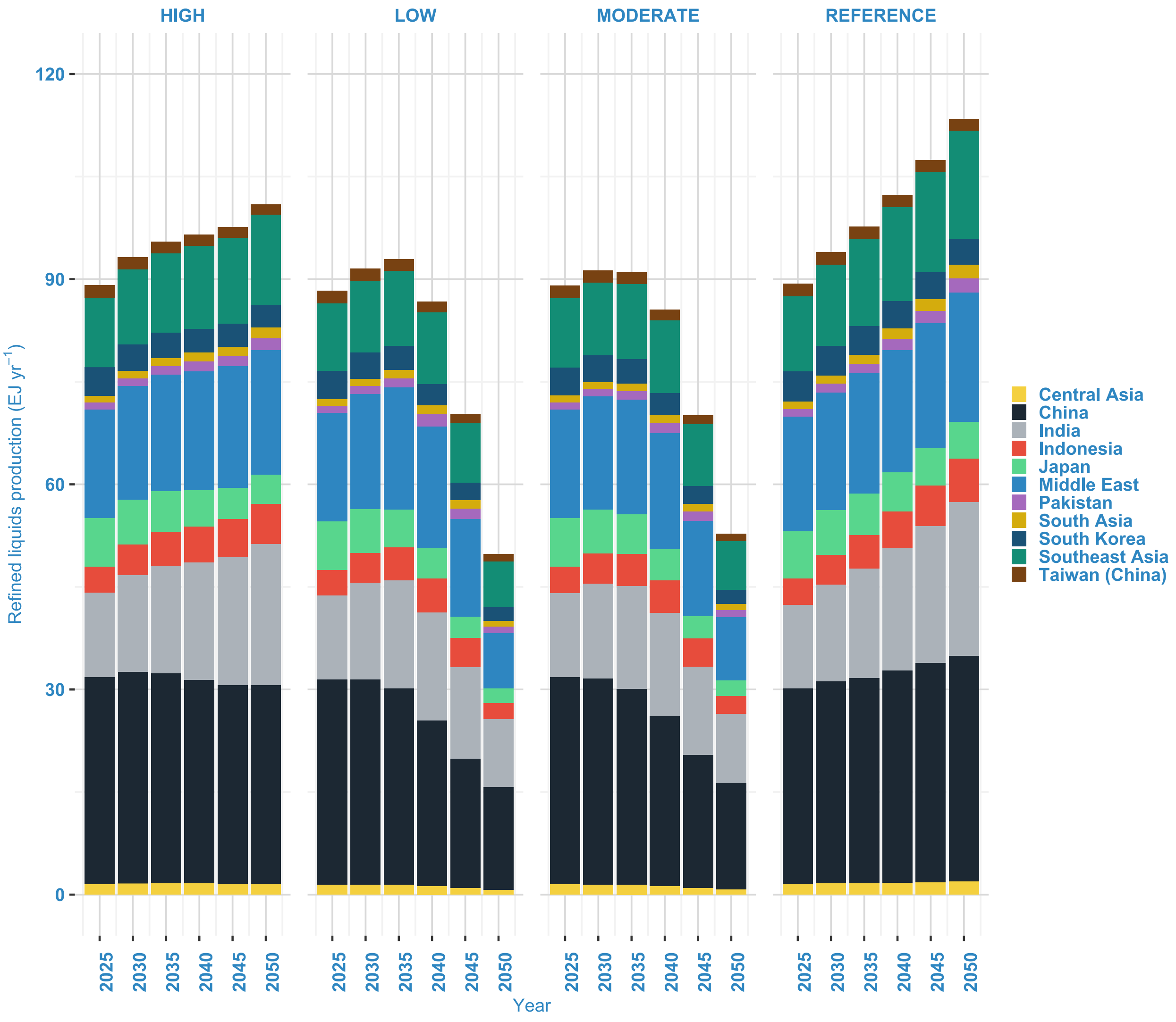

Supplementary Figure 7 Impact on refined liquids production. Refined liquids production by individual Asian country/region under varying levels of carbon dioxide removal (CDR) alongside a reference scenario. In Global Change Assessment Model (GCAM), refined liquids comprise biomass liquids, natural gas to liquids, oil refining, and coal to liquids, and these are carbon-based fuels. LOW and MODERATE CDR scenarios require rapid emission reduction in the absence of sufficient negative emissions leading to relatively lower demand for carbon-based fuels compared to HIGH CDR scenario. EJyr^{-1}: Exajoule per year

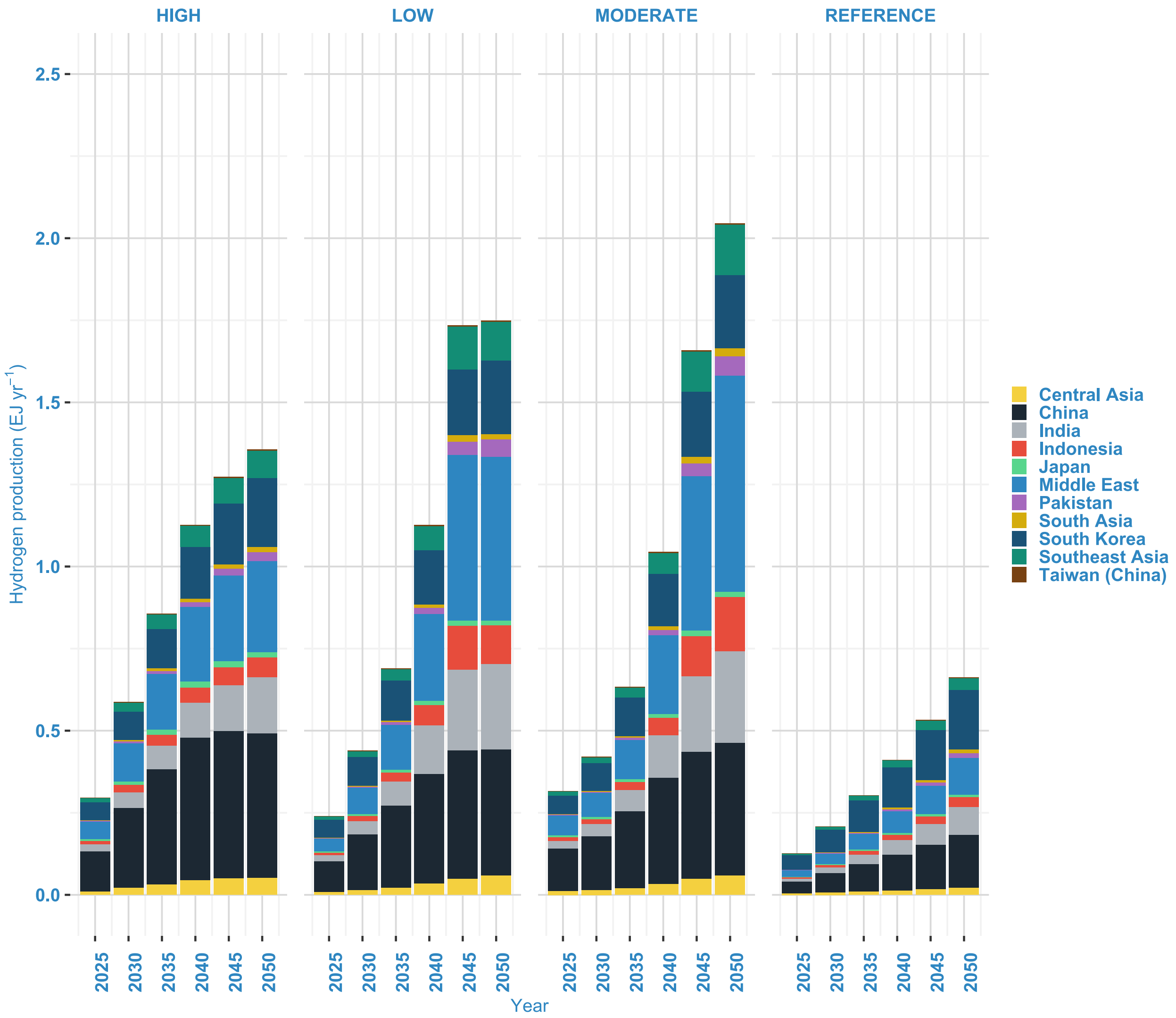

Supplementary Figure 8 Impact on hydrogen production. Hydrogen production by individual Asian country/region under varying levels of carbon dioxide removal (CDR) alongside a reference scenario. Similar to observations in electricity production, LOW and MODERATE CDR scenarios require rapid emission reduction in the absence of sufficient negative emissions leading to relatively higher requirement for hydrogen compared to HIGH CDR scenario. Hydrogen provides a means of deep decarbonizing sectors that are hard-to-electrify such as aviation and cement. With sufficient CDR as in the case of HIGH CDR scenario, it is relatively cheaper to balance the residual emissions from these sectors with CDR than to reduce their emissions with hydrogen deployment. $EJyr^{-1}$: Exajoule per year

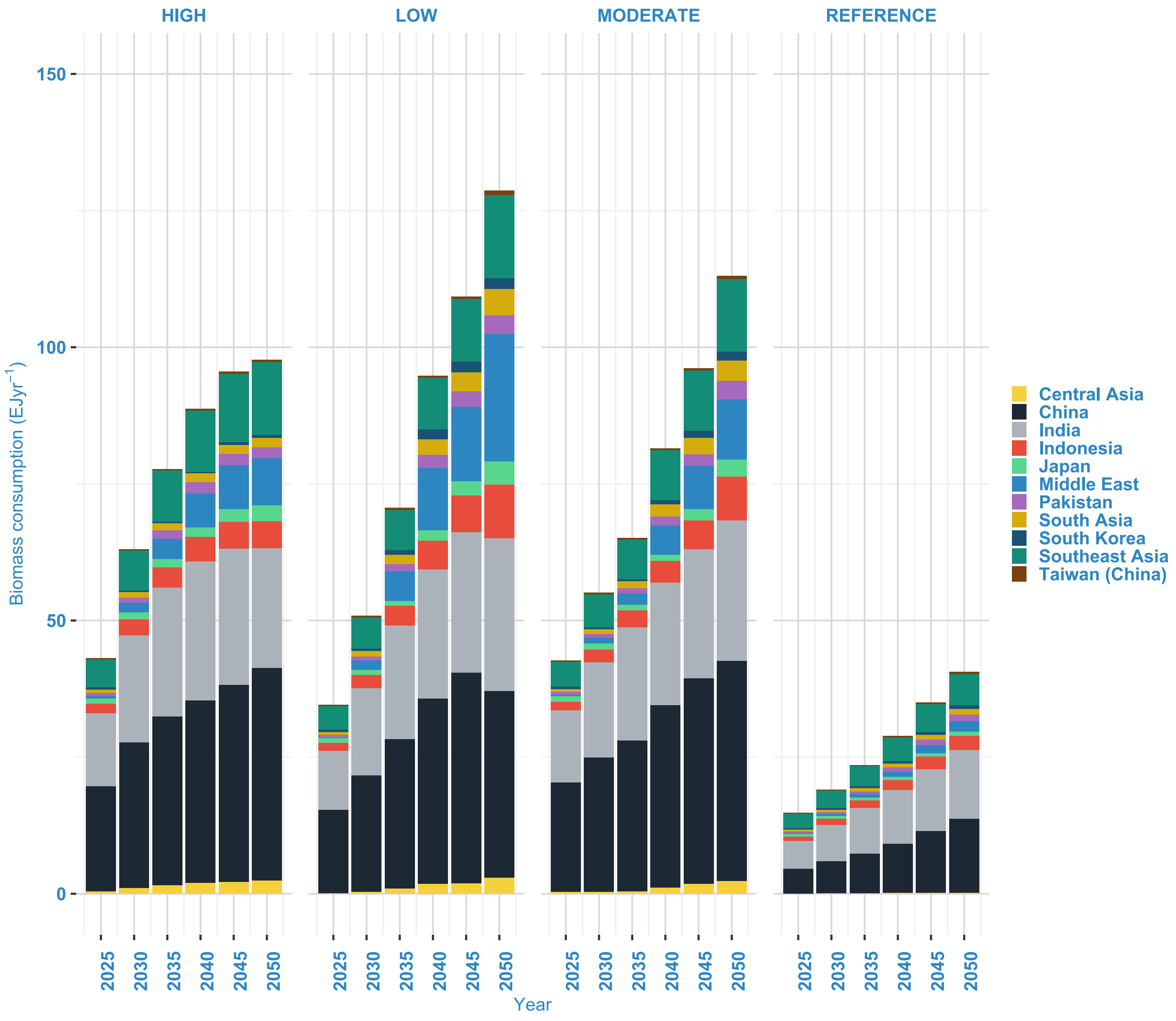

Supplementary Figure 9 Impact on biomass consumption. Consumption of biomass by individual Asian country/region under varying levels of carbon dioxide removal (CDR) alongside a reference scenario. Without sufficient CDR to offset residual emissions, low carbon sources, in this case, biomass and other zero carbon sources increase. This causes LOW and MODERATE CDR scenarios to exhibit relatively higher biomass consumption compared to HIGH CDR scenario especially towards mid-century. $EJyr^{-1}$: Exajoule per year

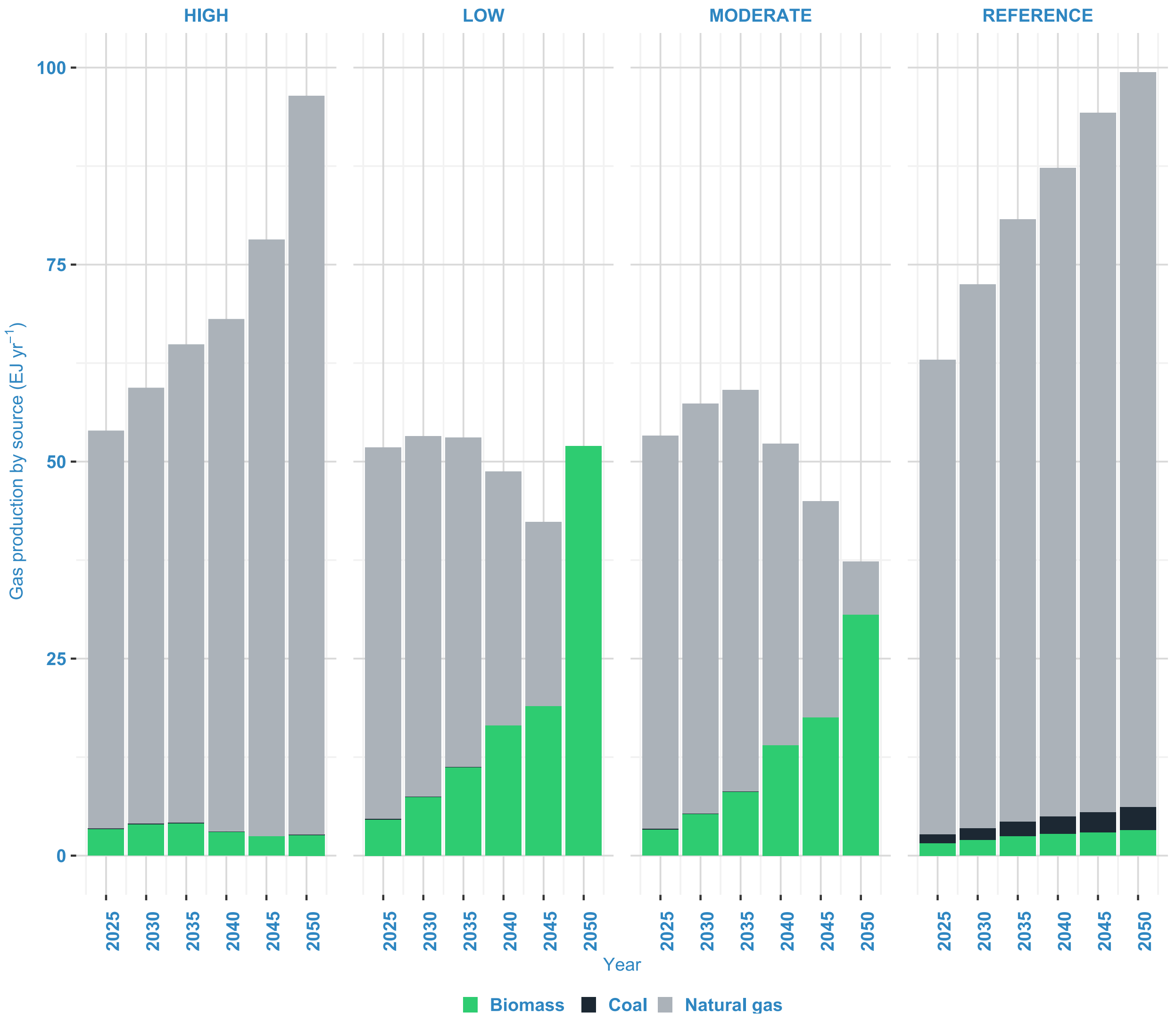

Supplementary Figure 10 Impact on gas production. Total gas production by source in Asia under varying levels of carbon dioxide removal (CDR) alongside a reference scenario. Rapid emission cuts required under LOW and MODERATE CDR scenarios imply that they are limited by the amount of high carbon-based production. Thus, total gas production in HIGH CDR remains the highest of the three CDR scenarios but the share of biomass in this production will remain higher under the LOW and MODERATE CDR scenarios due to their requirement for low and zero energy sources and fuels. Coal for gas production grows under REFERENCE scenario but completely phases out under LOW and MODERATE CDR scenarios. EJyr^{-1}: Exajoule per year

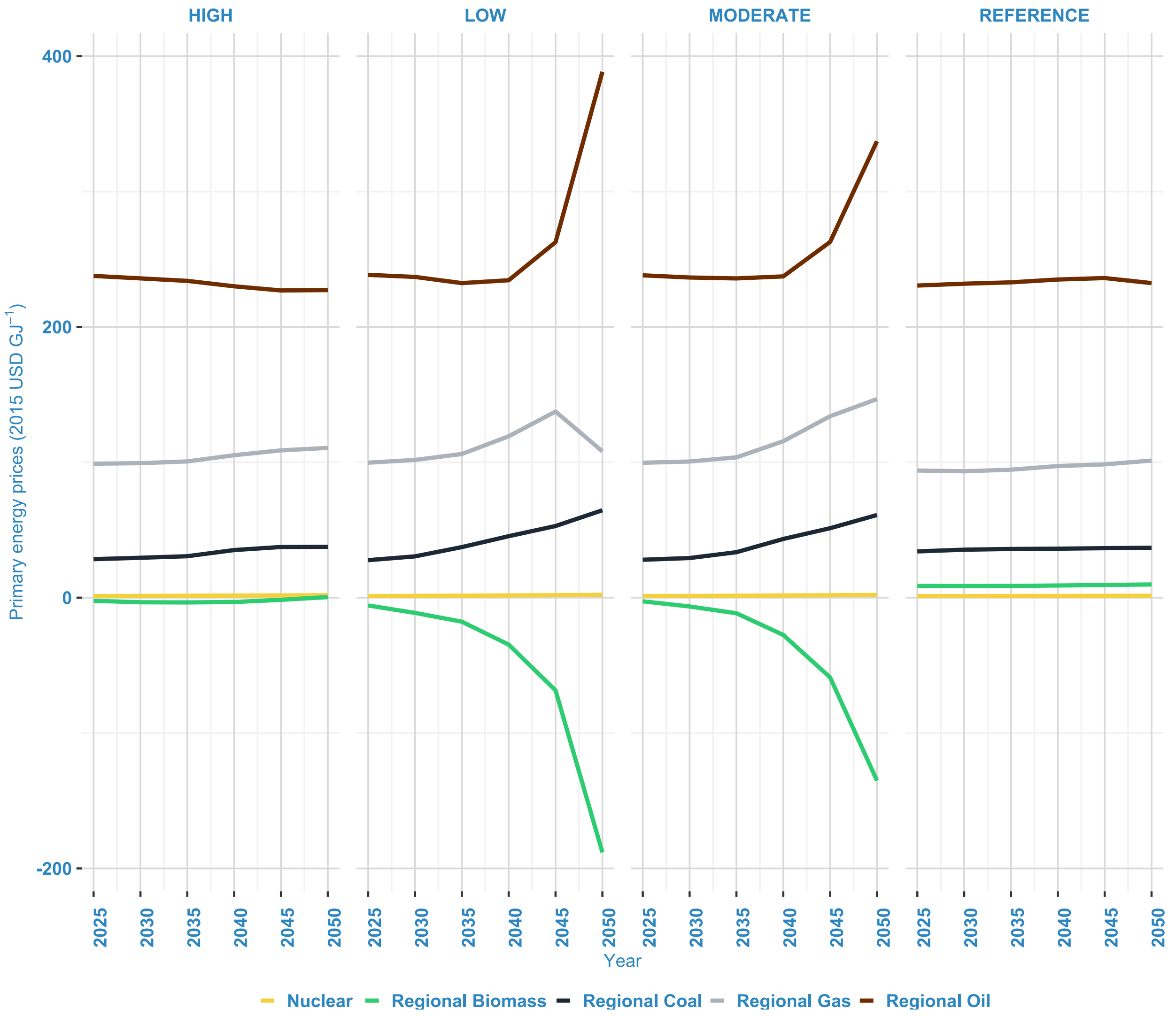

Supplementary Figure 11 Impact on primary energy prices. Average regional primary energy prices in Asia under varying levels of carbon dioxide removal (CDR) alongside a reference scenario. Without new climate mitigation policies (REFERENCE scenario), primary energy supply remains cheaper and the situation is similar to that of HIGH CDR scenario due to the slow phase down in fossil fuel consumption under this pathway. Regional coal and oil prices will significantly increase under LOW and MODERATE CDR scenarios due to the higher carbon prices under these two pathways. As a result of larger consumption of biomass under the MODERATE and LOW CDR scenarios, higher subsidies are made available for biomass consumption. USD GJ^{-1}: United States Dollar per Gigajoule

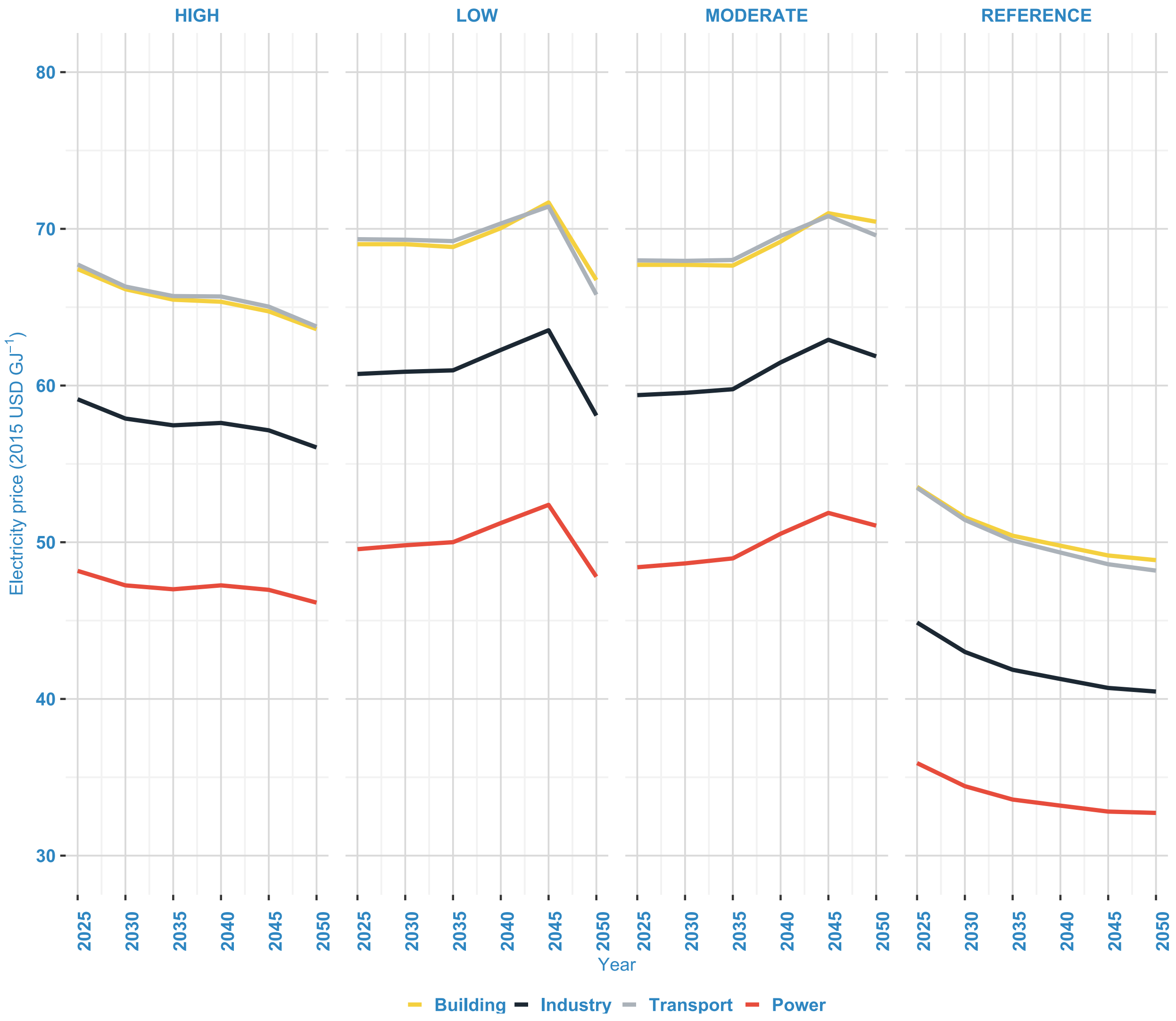

Supplementary Figure 12 Impact on electricity prices. Average electricity prices by sector in Asia under varying levels of carbon dioxide removal (CDR) alongside a reference scenario. Electricity prices increase with an increase in demand for electricity. Without new climate change mitigation policies (REFERENCE) electricity consumption across all sectors remains the lowest. Higher emission reduction requirements under LOW and MODERATE CDR scenarios implies that electricity prices for all sectors under these two pathways are higher than their corresponding prices under HIGH CDR scenario. In the near-term, as the cost of electricity producing/supply technologies is growing and demand is growing, electricity prices will keep on increasing until the technologies' costs start declining which will cause electricity prices to decline towards mid-century. USD GJ^{-1}: United States Dollar per Gigajoule

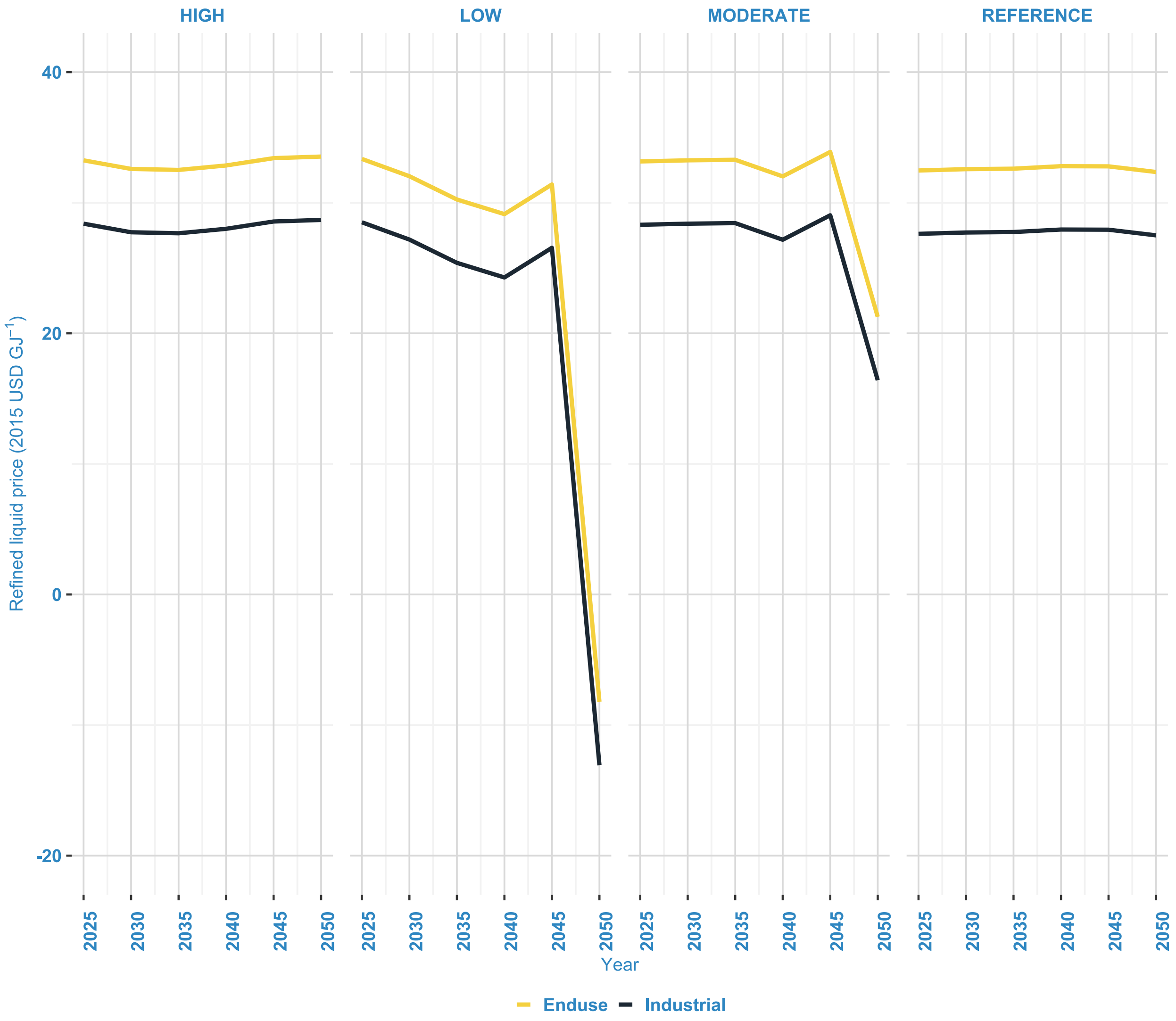

Supplementary Figure 13 Impact on refined liquid prices under refined liquids enduse/industrial. Average refined liquids prices in Asia under varying levels of carbon dioxide removal (CDR) alongside a reference scenario. In Global Change Assessment Model (GCAM), refined liquids comprise biomass liquids, natural gas to liquids, oil refining, and coal to liquids, and these are carbon-based fuels. LOW and MODERATE CDR scenarios require rapid emission reduction in the absence of sufficient negative emissions leading to relatively lower demand for carbon-based fuels compared to HIGH CDR scenario. Without new climate mitigation policies (REFERENCE scenario), refined liquids prices remain cheaper and the situation is similar to that of HIGH CDR scenario due to the slow phase down in fossil fuel consumption under this pathway. As a result of a larger consumption of biomass under the MODERATE and LOW CDR scenarios, higher subsidies are made available for biomass consumption, driving the total price of refined liquids into negative. USD GJ^{-1}: United States Dollar per Gigajoule

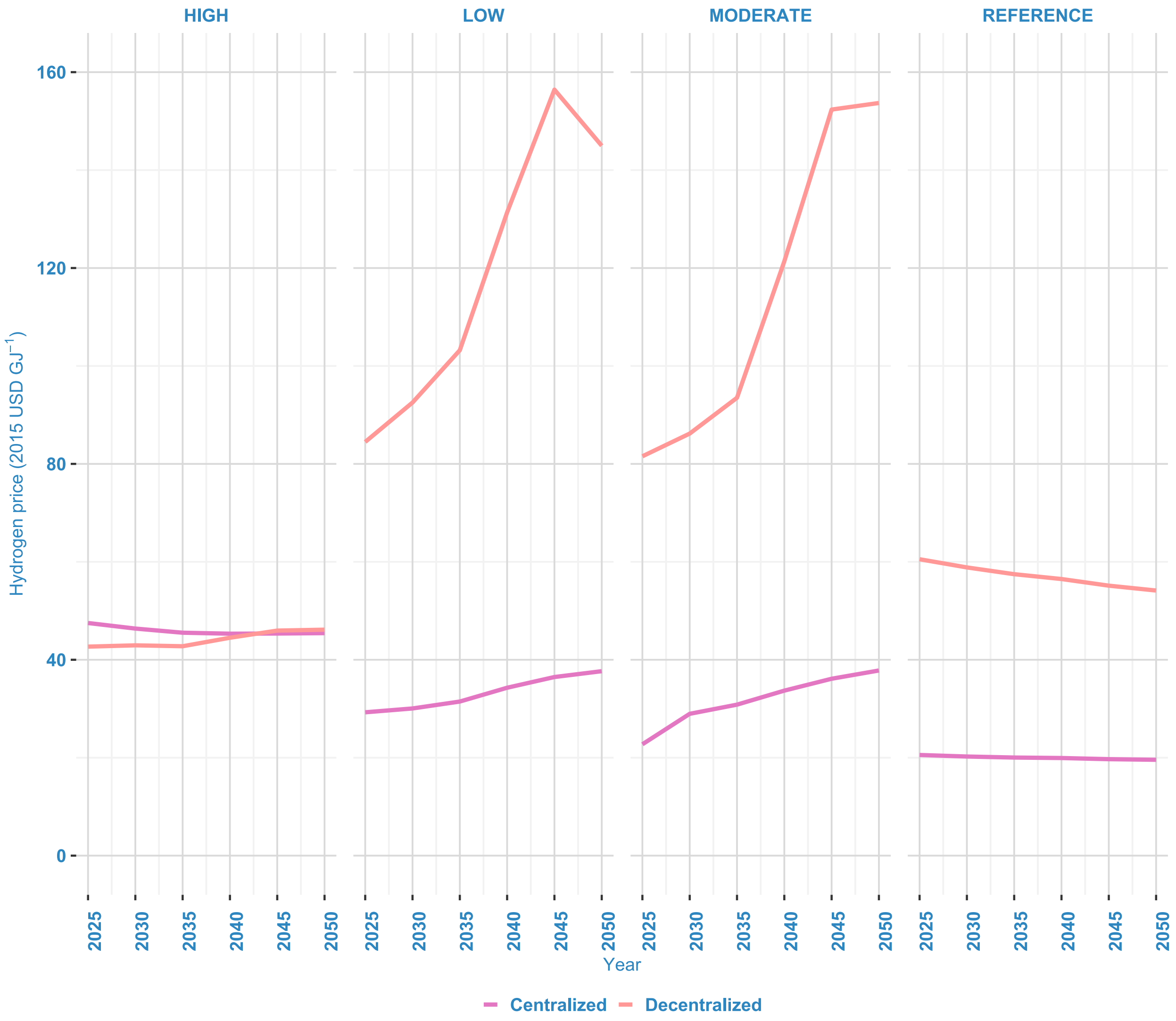

Supplementary Figure 14 Impact on hydrogen prices for centralized and decentralized hydrogen production. Average hydrogen production prices in Asia under varying levels of carbon dioxide removal (CDR) alongside a reference scenario. As a key fuel for decarbonizing hard-to-abate sectors, hydrogen has a critical role to play especially in scenarios where rapid emission cuts are required such as the MODERATE and LOW CDR scenarios. On average, the increasing demand for hydrogen increases hydrogen prices under LOW and MODERATE CDR scenarios compared to HIGH CDR, particularly hydrogen from decentralized systems. USD GJ^{-1}: United States Dollar per Gigajoule

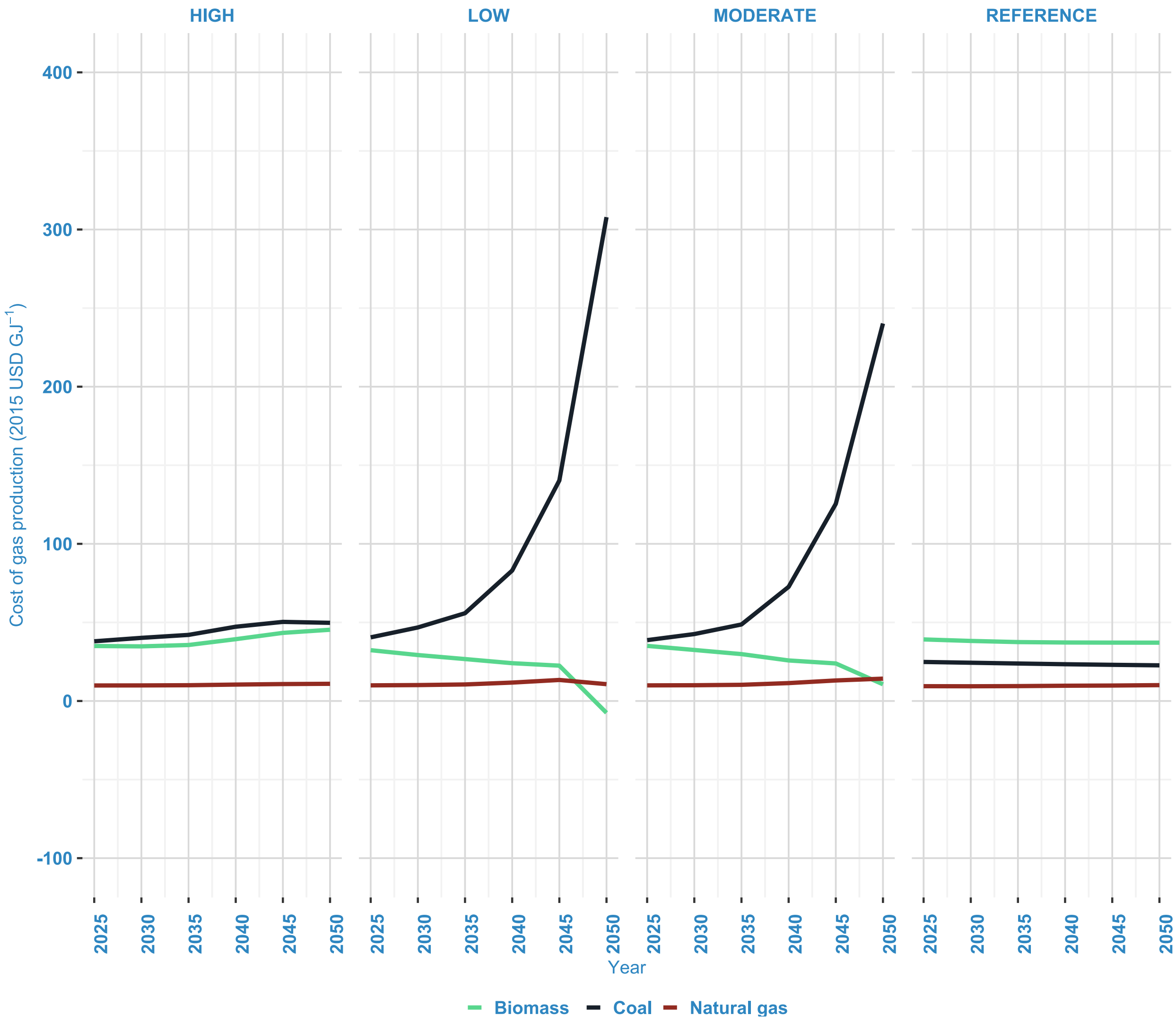

Supplementary Figure 15 Impact on gas production cost. Average gas production cost by source in Asia under varying levels of carbon dioxide removal (CDR) alongside a reference scenario. Higher requirements for emission reduction increase carbon prices making high carbon energy sources relatively expensive compared to lower carbon sources. Under HIGH CDR scenario, carbon prices are lower which causes lower prices in gas production compared to MODERATE and LOW CDR scenarios. The high carbon prices in LOW and MODERATE CDR scenarios increase the price of gas production from coal significantly compared to the price under HIGH CDR scenario. USD GJ^{-1}: United States Dollar per Gigajoule

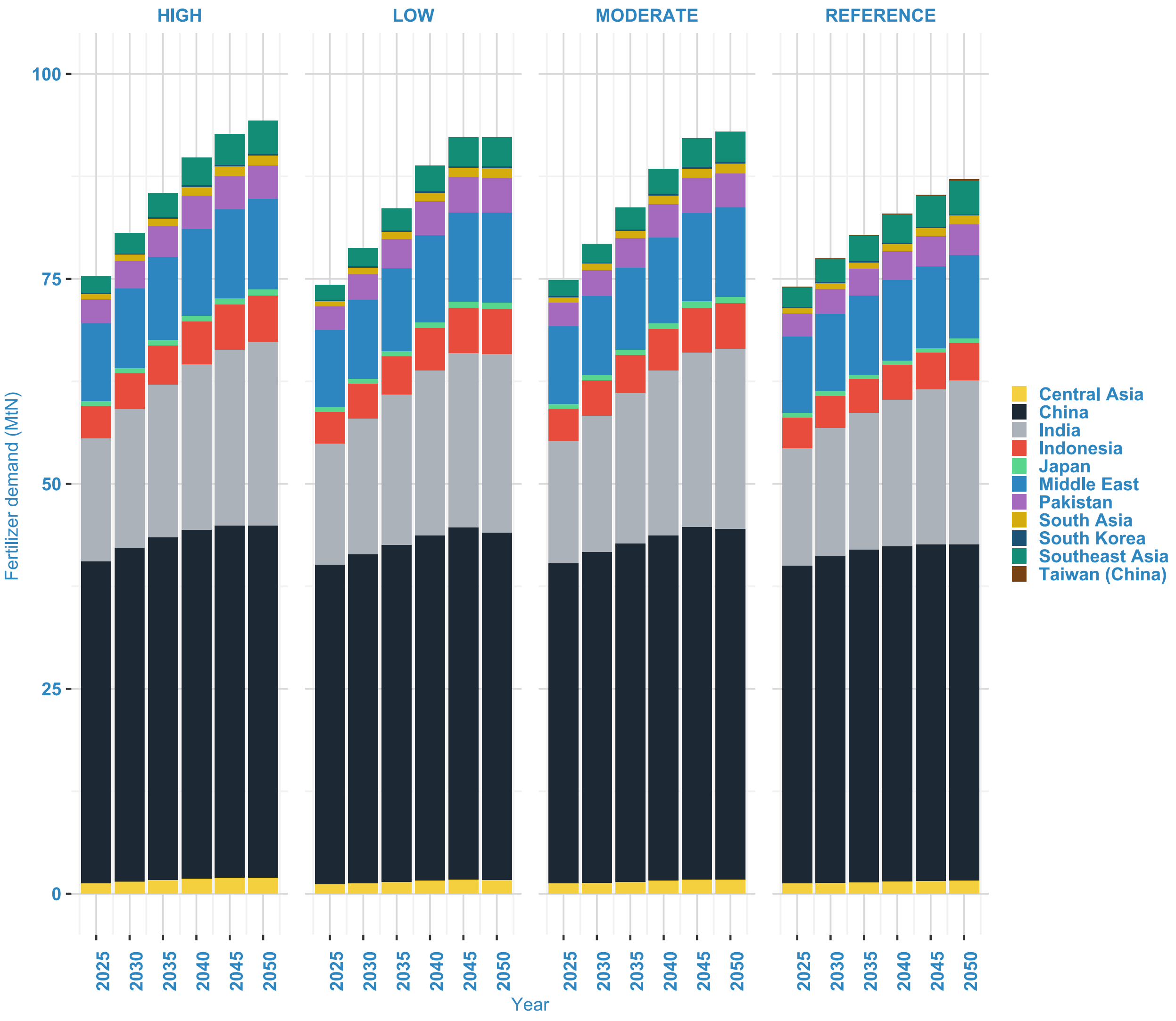

Supplementary Figure 16 Impact on nitrogen fertilizer demand. Nitrogen fertilizer demand by individual Asian country/region under varying levels of carbon dioxide removal (CDR) alongside a reference scenario. Overall, pursuing climate ambitions leads to an increase in fertilizer demand due to reasons such as fertilizer for growing bioenergy crops and this is reflected in the observation under the REFERENCE scenario compared to the net zero scenarios. In all scenarios, the demand for fertilizer is particularly higher in China and India due to their population and economic growth trajectory, requiring higher fertilizer for both food and climate change objectives. MtN: million tonnes of nitrogen

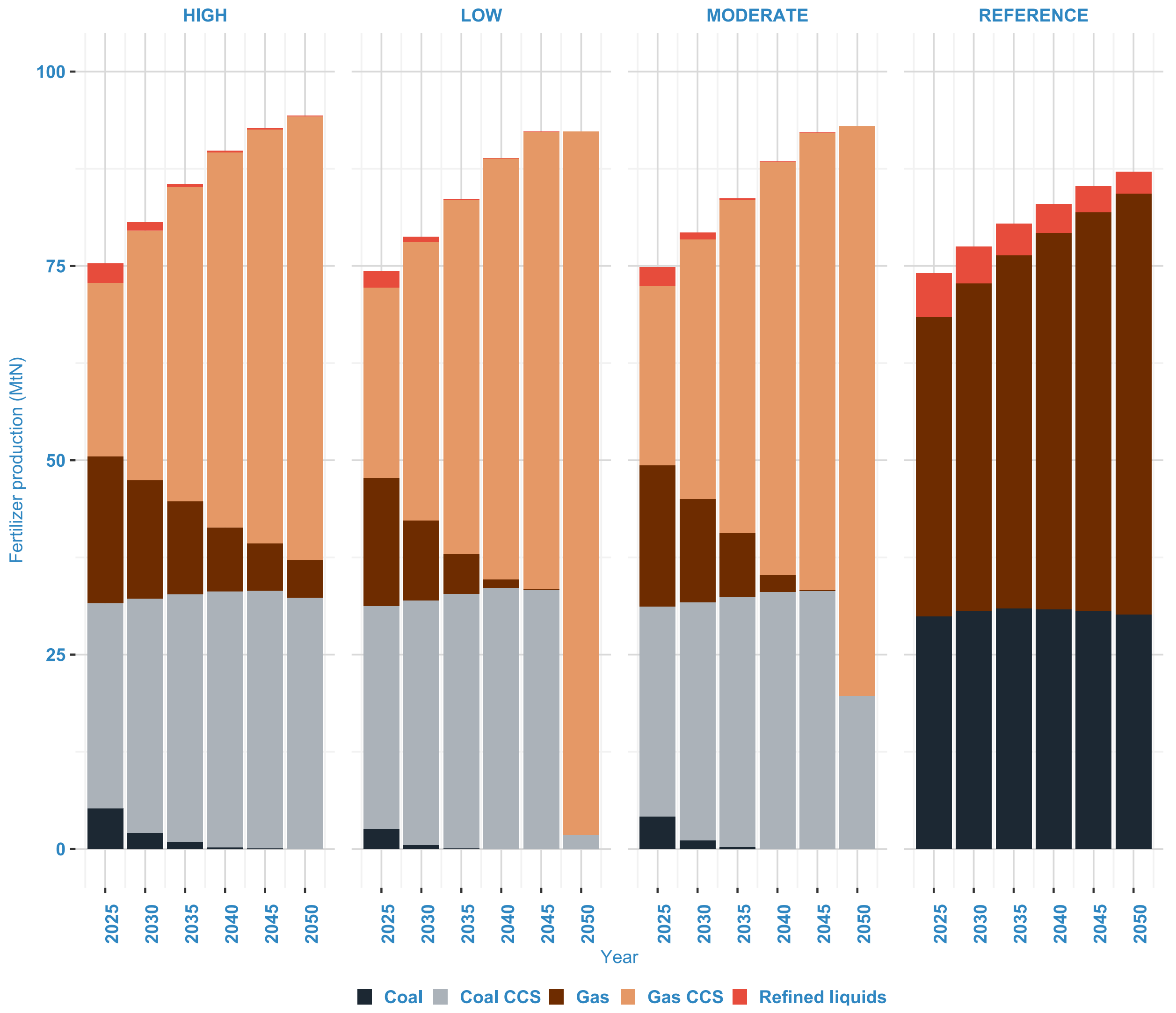

Supplementary Figure 17 Impact on fertilizer production. Fertilizer production by technology in Asia under varying levels of carbon dioxide removal (CDR) alongside a reference scenario. Higher requirements for emission reduction increase carbon prices making high carbon energy sources relatively expensive compared to lower carbon sources. Under HIGH CDR scenario, carbon prices are lower which causes higher fertilizer production from high carbon sources which are unabated compared to the situation under MODERATE and LOW CDR scenarios. There is rapid phase out of coal (especially unabated) for producing fertilizer under MODERATE and LOW CDR scenarios. These scenarios rely mostly on abated natural gas for producing fertilizer compared to the observation under HIGH CDR scenario. CCS: carbon capture and storage. CCS: carbon capture and storage; MtN: million tonnes of nitrogen

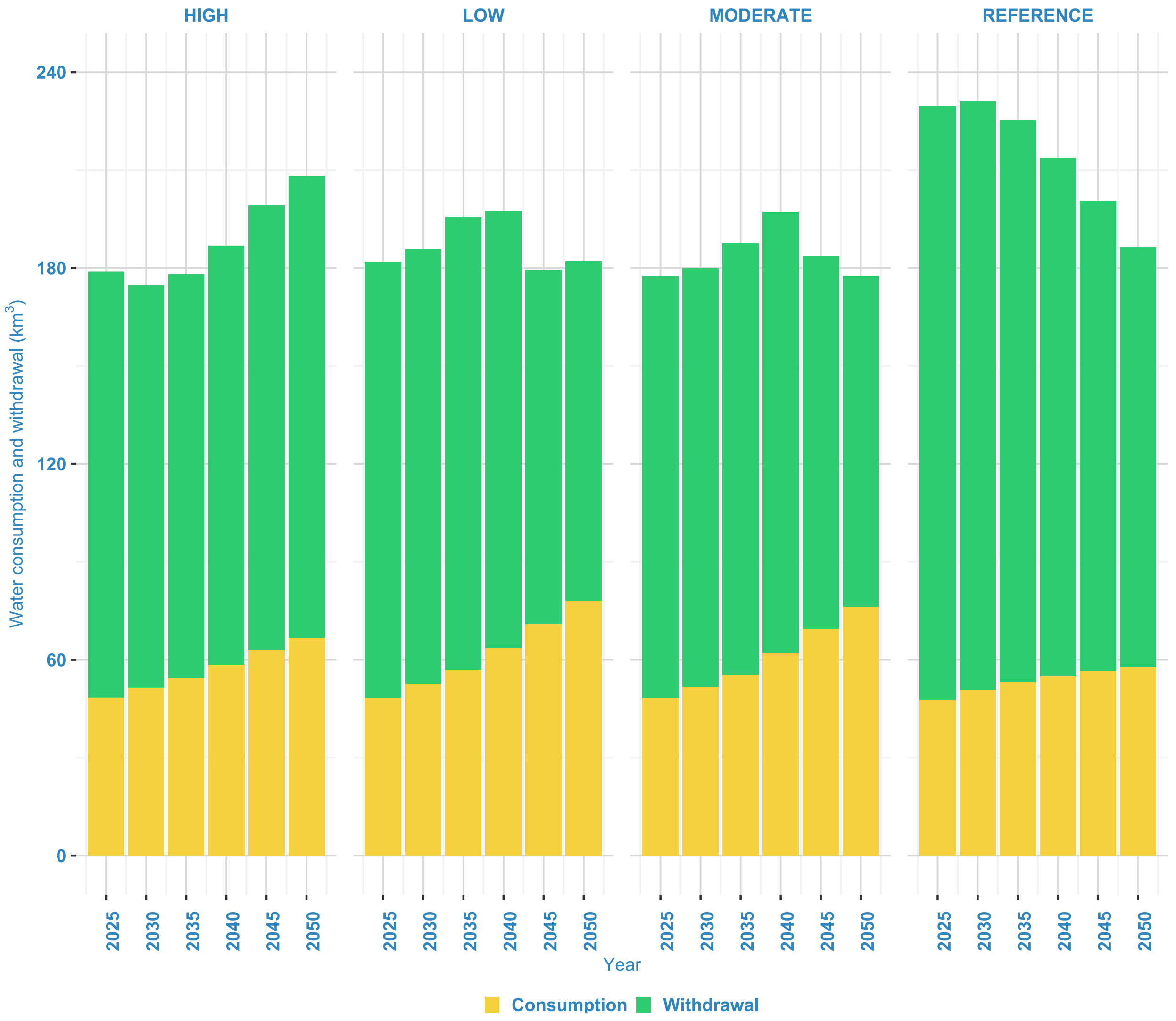

Supplementary Figure 18 Impact on water demand. Total water consumption and withdrawal by Asia's power sector under varying levels of carbon dioxide removal (CDR) alongside a reference scenario. Here, water use by humans or livestock or lost to evaporation is referred to as water consumption whiles water withdrawals implies any water withdrawn from surface water resources or from the ground and subsequently returned to the natural environment later [1]*. km^3: cubic kilometers*

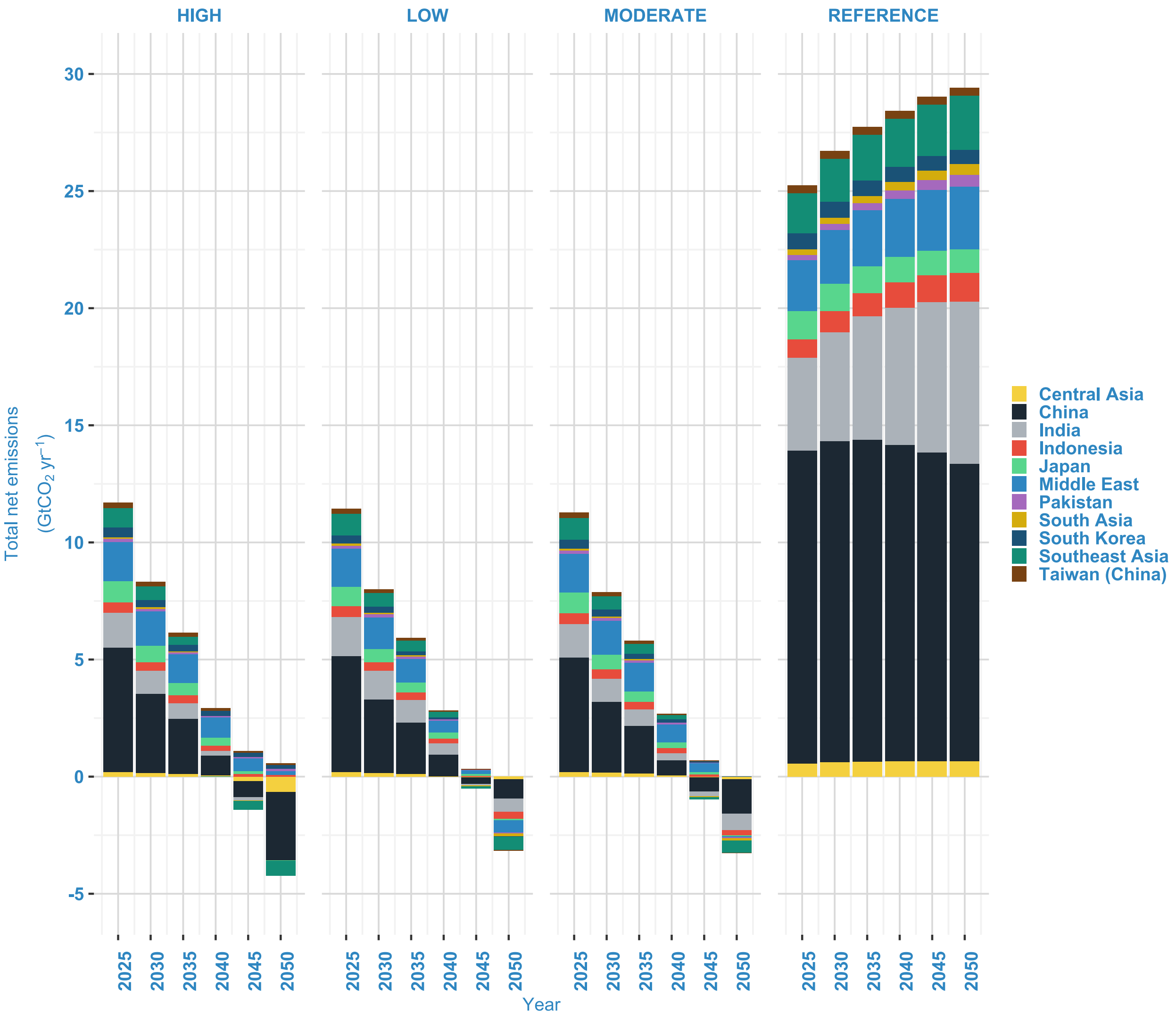

Supplementary Figure 19 Impact on net emissions. Total net CO_2 emissions by individual Asian country/region under varying levels of carbon dioxide removal (CDR) alongside a reference scenario. One of the key roles of CDR is to accelerate a reduction in net emissions. The large-scale reliance on CDR would produce deeper net negative emissions primarily led by China, but due to the associated moral hazard and low carbon prices, it is more cost-effective for countries to forego reaching net zero CO_2 by mid-century while they pursue carbon offsets from overseas. On the other hand, under LOW CDR scenario, all countries achieve domestic net zero CO_2 before 2050 due to the need for rapid emission reductions; $GtCO_2yr^{-1}$: gigatonnes of carbon dioxide per year

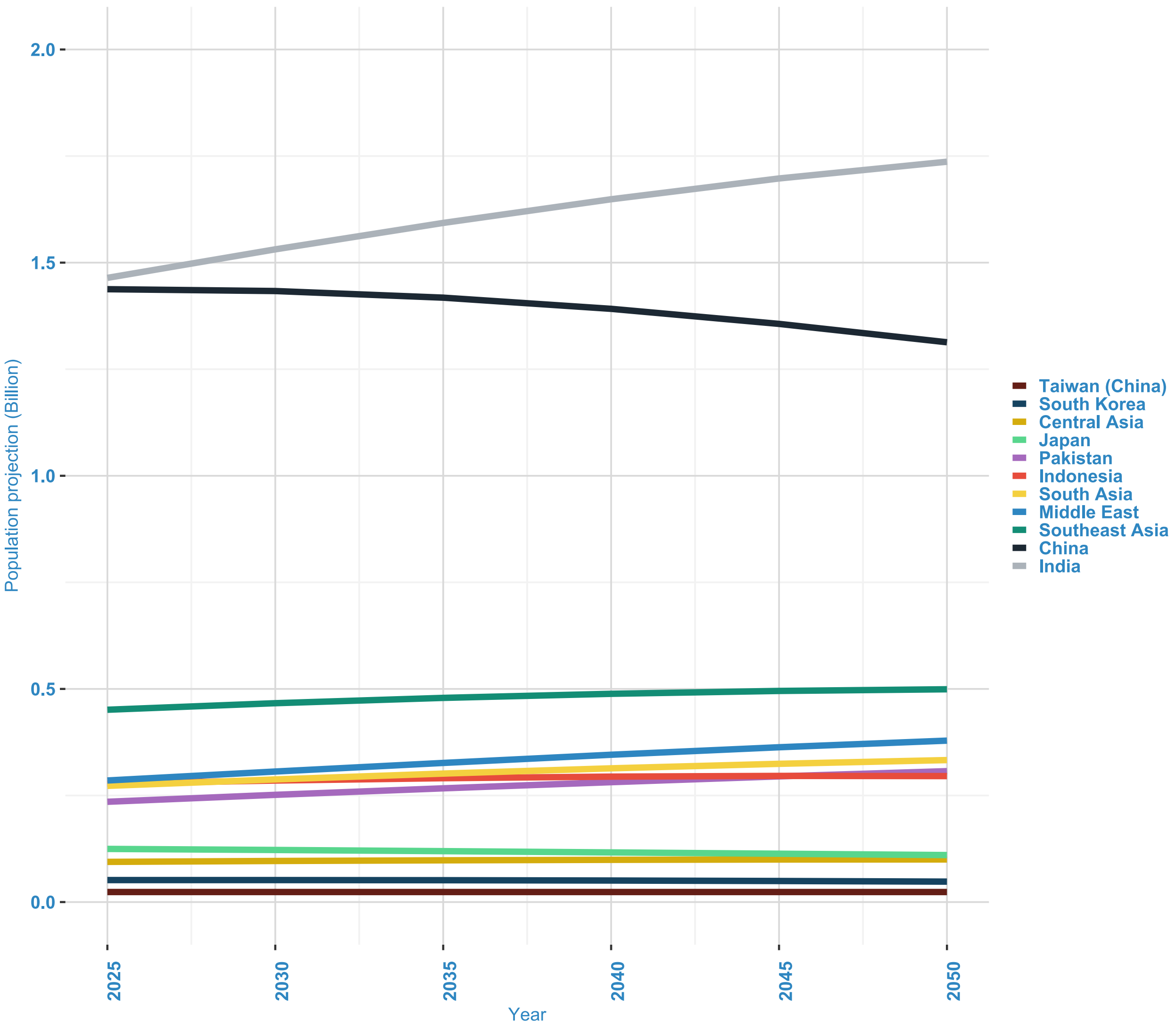

Supplementary Figure 20 Population projection in Asia. Population plays a key role in resource supply and demand in Global Change Assessment Model (GCAM). All scenarios have been modeled following a population trajectory under Shared Socio-economic Pathway 2 (SSP2). Across Asia, China's population is set to rapidly decline over time while others remain fairly constant or increase over time such as India.

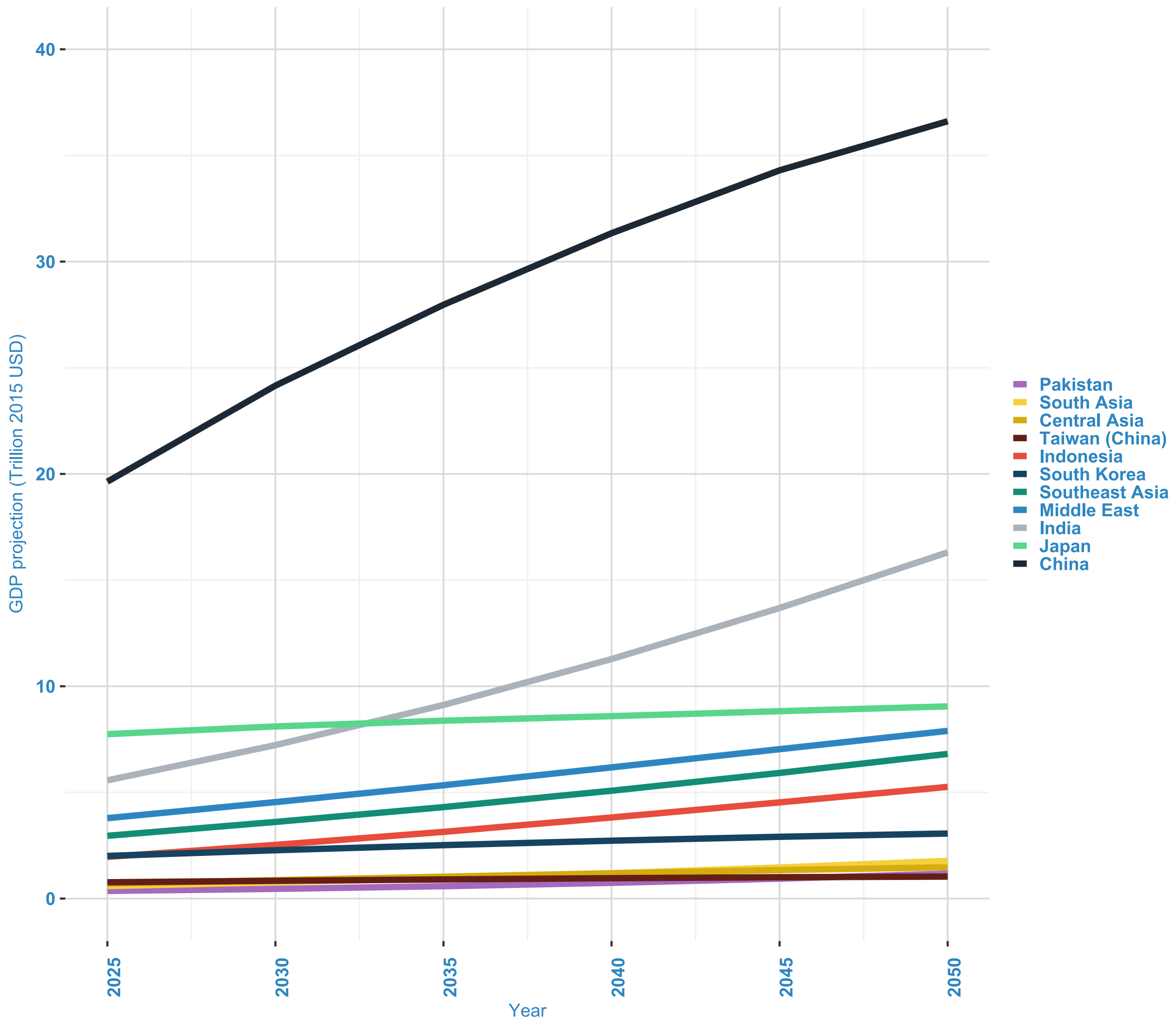

Supplementary Figure 21 *Gross domestic product (GDP) projection in Asia. GDP plays a key role in resource supply and demand in Global Change Assessment Model (GCAM). All scenarios have been modeled following a GDP trajectory under Shared Socio-economic Pathway 2 (SSP2). China's economy is bound to increase significantly which would mean increased energy demands and emissions. As such, China, followed by India, will be responsible for the large-scale deployment of both emission reduction and removal technologies to enable Asia to stay on track for our stylized net zero pathway for the continent. USD: United States Dollars*

Supplementary Discussion 1: Impact on primary and final energy demand, and abatement costs

The phenomenon of intensifying carbon lock-in is a significant risk associated with excessive reliance on CDR, as indicated by the results. Fig. 1a demonstrates that high CDR reliance indeed presents a risk by encouraging continued reliance on fossil fuels, thereby impeding the transition to sustainable energy systems. By attempting to remove large quantities of CO_2 from the atmosphere, the remaining carbon budget is extended [2], delaying the necessary transition away from fossil fuels to achieve climate targets. In the REFERENCE scenario, the share of renewables in primary energy consumption in Asia will reach approximately 17% by 2050 (Fig. 1a). Interestingly, the HIGH scenario, which follows a net-zero GHG pathway, achieves similar levels of renewable energy share at 19% by mid-century. Conversely, the shares increase to 30-40% in the MODERATE and LOW scenarios. The share of nuclear power is approximately twice as high in the MODERATE and LOW scenarios compared to the HIGH scenario. A slightly higher capacity of carbon capture and storage (CCS) technologies would be deployed in the HIGH scenario due to its greater reliance on carbon-based energy sources to stay on track for the net-zero goal.

Due to its relatively lower end-use sector electrification, electricity production in the HIGH scenario is relatively lower than the corresponding production under the MODERATE and LOW scenarios (Fig. 1b). By 2050, about 188-193 EJ of electricity would be produced in Asia under the MODERATE and LOW scenarios compared to 160 EJ under the HIGH scenario. Renewable and nuclear-based electricity production is also very significant under the MODERATE and LOW scenarios (170-175 EJ by 2050) compared to 125 EJ under the HIGH scenario. As a consequence, the HIGH scenario exhibits higher fossil-based electricity production.

The observation from electricity production is consistent with that of hydrogen production as shown in Fig. 1c. By 2050, hydrogen production in Asia from electrolysis or thermal splitting could reach nearly 2 EJ under the MODERATE and LOW scenarios, compared to less than 0.5 EJ under the HIGH scenario.

Exactly 50% of total hydrogen production by 2050 will be sourced from coal and natural gas under high CDR reliance compared to less than 20% when the reliance is minimized.

The results are further emphasised spatially in the Asian regions and countries. The findings indicate that countries such as China and India could satisfy their primary energy demands in 2050 with 33% and 45% coming from renewables (wind, hydro, geothermal, solar) under the MODERATE scenario, compared to 17% and 27% under the HIGH scenario, respectively (Fig. 2a-c). Furthermore, all Asian countries and regions exhibit slightly higher consumption of renewables under the MODERATE scenario compared to the LOW scenario, except for China and India. This is due to their pronounced carbon lock-in systems in comparison to other countries or regions on the continent. Fig. 2d-f also illustrates the share of electrified transportation by 2050 under different levels of CDR reliance. Under the HIGH scenario, the highest share of electrified transport in any Asian region or country remains below 15%, with Central Asia and Japan having the highest shares at 12.90% and 8.64%, respectively. Countries like China and India only exhibit shares of 4.50% and 3.75% for electrified transport, respectively, due to their highly carbon-intensive transport systems. However, under the MODERATE and LOW scenarios, the highest share of electrified transport can rapidly increase to 42-47%, with China and India's shares reaching about 25-30% and 30-35%, respectively. The observation is similar to the results related to the percentage reduction in building energy consumption by 2050 in the net-zero scenarios compared to the REFERENCE scenario (Fig. 2g-i). The results show that under the HIGH scenario, the highest reduction in building energy consumption would be recorded by Central Asia, South Asia, and China at 25.39%, 22.6%, and 22.50%, respectively. This reduction rate significantly increases to 38.8%, 50%, and 34% in these regions, respectively, under the MODERATE scenario.

Supplementary Figure1 illustrates the total final energy consumption by fuel type, and the observations align with those seen in primary energy. Without new climate policies (REFERENCE), total final energy consumption in Asia could reach nearly 350 EJ by 2050. A net-zero GHG ambition in the region would significantly facilitate a transition toward cleaner fuels and energy-efficient measures. However, in the

HIGH scenario, the total final energy consumption by 2050 is only 5% lower than the total demand in the REFERENCE scenario, whereas the MODERATE and LOW scenarios show reductions of 35%. While high reliance on negative emissions prolongs the continuous consumption of fossil fuels for final energy needs, energy-efficient practices and technologies are not highly prioritized either.

In the MODERATE scenario, Asia's final energy demands could be met with 162 EJ from electricity and hydrogen, which is 16% higher than the case in the HIGH scenario. While the energy consumption in the transport sector diminishes toward mid-century under the MODERATE and LOW scenarios, consumption in the sector continues to increase under the HIGH scenario, following the same pattern as in the absence of new climate policies (REFERENCE). Nearly 30 EJ of electricity and natural gas would be consumed under the HIGH scenario by 2050 solely for removing CO_2 from the atmosphere. This can be attributed to the higher reliance on energy-intensive CDR approaches like DACCS for achieving negative emissions [1,3,4].

On the other hand, the reluctance to transition away from fossil fuel energy systems to sustainable energy alternatives hinges on the relative cost-effectiveness of fossil fuels and their infrastructure compared to their cleaner counterparts [5–7]. Relating to Supplementary Figure 2a, in the absence of significant negative emissions, other non-CDR mitigation strategies are needed at scale to significantly reduce emissions and lower the need for negative emissions in the first place [8–10]. Unfortunately, these strategies are relatively expensive in the near term [11–13] compared to scenarios where cheaper carbon alternatives would continue and later offset all related emissions due to the availability of adequate negative emission solutions. The existing infrastructure is predominantly tailored to fossil fuel energy systems (carbon lock-in) [14–17], making the transition to carbon-free sources more challenging and expensive due to necessary overhauls and new installations. Furthermore, fossil fuel industries benefit from established economies of scale [18], which clean energy solutions are still developing. Results in Supplementary Figure 2a show the optimal marginal abatement cost of carbon required by each scenario to reach climate goals. The marginal abatement cost of carbon is the cost required to reduce the last unit of emissions to reach a climate target, and this cost is zero

for scenarios with no plans for new climate policies (REFERENCE) [8]. The results reveal a significant gap in how much is required to reduce one ton of CO_2 from the atmosphere in each pathway, with the HIGH scenario showing just over \$300 per tCO_2 by 2050, compared to over \$2500 per tCO_2 and \$3300 per tCO_2 in the MODERATE and LOW scenarios, respectively. Qiu et al. recently showed that the availability of negative emissions in the US electricity system presents economic advantage in mitigating the last few percent of CO_2 from the sector [11]. As the production and adoption of renewable energy increase, the costs are likely to decrease due to improved efficiency and technological advancements. Already, renewables and other clean energy solutions are increasingly becoming more affordable A recent report by IRENA illustrates that nearly 66% of the renewable energy capacities installed in 2021 were more cost-efficient than the most economical coal-powered alternatives in G20 nations. The expenses associated with electricity from onshore wind experienced a reduction of 15%, while those from offshore wind and solar photovoltaics (PV) decreased by 13% each in comparison to 2020 [19]. With continuous developments, learning curves, and economies of scale, alongside proper policy schemes, the marginal cost gap, as seen in the results, could be bridged. The results here emphasize that abatement cost is a major co-benefit that comes with high CDR reliance.

Supplementary Discussion 2: Impact on positive and negative emissions, net zero timing, and pollutants

Results in Supplementary Fig. 2b, which show positive GHG emissions by sector and species (including bio-derived CO_2 emissions), indicate that Asia's total GHG emissions would continue to grow under the REFERENCE scenario, reaching about 40 $GtCO_2e$ by 2050, approximately 80% of today's global GHG emissions. Significant reductions in emissions are expected under the net-zero pathways, and a net-zero GHG ambition by mid-century in Asia could reduce the region's total GHG emissions to less than 20 $GtCO_2e$ by 2050.

Economically speaking, undue substitution is a typical characteristic of CDR systems, allowing for continued fossil emissions through carbon offsetting [20,21]. As a result, while total positive GHG emissions (including biogenic CO_2) under the HIGH scenario reach 16 $GtCO_2e$ by 2050, those in the MODERATE and LOW scenarios reach 11 and 13 $GtCO_2e$, respectively. Methane emissions would continue to decline in the MODERATE and LOW scenarios, reaching 2.5 $GtCO_2e$ and 2.4 $GtCO_2e$, respectively, by 2050, while in the HIGH scenario, they increase throughout the period, reaching 3.3 $GtCO_2e$ by mid-century.

In terms of sectoral emissions, the most significant reductions in emissions in a low to moderate CDR reliant-environment (LOW and MODERATE) compared to a highly reliant-environment (HIGH) would be observed in the building and transport sectors. Transport sector emissions under the HIGH scenarios increase by 15% between 2025-2050, while emissions from buildings reduce by nearly 25% during the same period. In comparison, due to its higher affinity for electricity, hydrogen, and energy efficient approaches, transport and building sector emissions reduce by 85% and 90%, respectively, in the MODERATE scenario during the same period.

Total gross CO_2 removal by CDR approaches is also represented in Fig. 3a. It should be noted that the results only capture CDR approaches, so the CO_2 removal by bioenergy crops for their growth via photosynthesis is not included. Total gross CO_2 removal under the HIGH scenario would quickly scale to approximately 12 $GtCO_2$ by 2050, compared to 2.3 $GtCO_2$ and 0.5 $GtCO_2$ in the MODERATE and LOW scenarios, respectively. Under the HIGH scenario, BECCS (4.6 $GtCO_2$), DACCS (4.2 $GtCO_2$), and ERW (2.2 $GtCO_2$) would play the most important roles, collectively reaching 11 $GtCO_2$ of removal potential by 2050. DORCS would play a minor role, with deployment of under 2 $MtCO_2$ by 2050 in Asia. This could be attributed to high system cost and operation of DORCS (which is dominated by systems coupled with desalination plants). In these systems, desalination and CO_2 removal both share the cost and energy required for a successful operation of the plant. DORCS technology without such configurations (stand-alone) is relatively very expensive to deploy [22]. As such, the deployment of DORCS (co-located with desalination plants) is limited by the demand for desalinated water, and regions like the Middle East with high demands

for desalinated water benefit the most from this limitation. About 16 km^3 of desalinated water is required by the Middle East in 2050, and 40% of this demand would be met by DORCS (co-located with desalination plants). In perspective, the next region or country on the continent with the highest demand for desalinated water is Central Asia at 3.6 km^3 by 2050. Hence, about 70% of the total DORCS deployment for negative emissions in Asia by 2050 would be located in the Middle East alone. The presence of multiple CDR approaches in the HIGH scenario reduces the overall role of net negative emissions from land use change (LUC). Despite having significantly lower total gross CO_2 removal, about 452 million tonnes CO_2 ($MtCO_2$) of net negative emissions could be sourced from LUC under the MODERATE scenario, compared to 437 $MtCO_2$ in the HIGH scenario. The availability of multiple CDR approaches displaces some conventional sources of negative emissions, such as afforestation and reforestation, which reduces net negative emissions from LUC.

In the absence of new climate policies, Asia's total air pollutants, including black carbon (BC), ammonia (NH_3), non-methane volatile organic compounds (NMVOCs), nitrogen oxides (NOx), organic compounds (OC), and sulfur dioxide (SO_2), would decline from 237 Teragram (Tg) to 213 Tg between 2025-2050, with all pollutants decreasing during the 25-year period except NH_3 (Fig. 3b). Between 2025-2050, all air pollutants reduce in quantity in the net-zero pathways except NH_3 under the MODERATE and LOW scenarios, which show about a 14-16% growth between the two periods. Due to its high reliance on carbon-based energy sources, fuels, and inefficient practices, total air pollutants in the HIGH scenario reach 140 Tg by 2050, compared to 107 Tg and 103 Tg in the MODERATE and LOW scenarios, respectively. For all scenarios, the major contributor to total air pollutants is NMVOCs, except under the MODERATE and LOW scenarios – where NH_3 becomes the largest contributor between 2045-2050. During the pathway to net-zero emissions by mid-century in Asia, the highest emission reduction potential for any type of pollutant would be recorded in BC (> 75% between 2025-2050) under MODERATE and LOW followed by OC (68-75%). BC and OC emissions often result from incomplete combustion processes, such as those in diesel engines and solid fuel burning [23,24]. The significant reduction in fossil fuel consumption in the MODERATE

and LOW scenarios could lead to a substantial reduction in BC and OC emissions. The high reliance on fossil and solid fuels in the HIGH scenario leads to lower BC and OC reduction potential. The LOW and MODERATE scenarios also show a reduction of 55%-57%, 63-65%, 58-62% of NMVOC, NO_x, and SO_2, respectively from 2025 to 2050. In comparison, only 24%, 28%, and 37% of reduction of these air pollutants are achieved under the HIGH scenario during the same period.

Fig. 5a-c represents how total positive CO_2 emissions in Asia by 2050 are distributed among the countries and regions (includes biogenic CO_2). The results show that under the HIGH scenario, the following countries' (or regions') annual CO_2 emissions would exceed 1 $GtCO_2$ by mid-century: China (4.1 $GtCO_2$), India (2.4 $GtCO_2$), the Middle East (1.2 $GtCO_2$), and Southeast Asia (1.2 $GtCO_2$). The same four countries or regions individually cross the annual 1 $GtCO_2$ threshold by 2050 under the LOW scenario; that is, China (2.3 $GtCO_2$), India (1.7 $GtCO_2$), the Middle East (1.4 $GtCO_2$), and Southeast Asia (1.0 $GtCO_2$). Interestingly, among all countries and regions, only the Middle East and South Asia would record higher total positive CO_2 emissions by 2050 under the LOW scenario compared to their respective values under the HIGH scenario. This indicates the difficulty in significantly reducing emissions in those regions without the deployment of novel CDR technologies. For all countries and regions, the total positive CO_2 emissions would be lowest under the MODERATE scenario compared to their respective outputs in the HIGH and LOW scenarios. Under the MODERATE scenario, only China's (2.2 $GtCO_2$) and India's (1.4 $GtCO_2$) total positive emissions exceed 1 $GtCO_2$ by 2050. The results here indicate that the largest share of CO_2 removal in Asia would be concentrated within China, India, the Middle East, and Southeast Asia to offset their relatively higher gross positive emissions (fossil fuel and industry portion) compared to the other countries or regions on the continent. The distribution of total gross CDR by 2050 among Asian countries and regions is also shown in Fig. 5d-f. Under the HIGH scenario, CO_2 removal by mid-century would be mainly concentrated in China (6 $GtCO_2$), India (1.8 $GtCO_2$), and the Middle East (0.9 $GtCO_2$). Under the MODERATE scenarios, these removal capacities in the three countries/regions significantly reduce to 1.2, 0.5, and 0.08 $GtCO_2$, respectively, which could be attributed to the significant reduction in gross positive

emissions. It is interesting to note that under the LOW scenario, regions or countries like India, Japan, Pakistan, and South Korea show 'negative' values – indicating higher net positive land use emissions by these regions (i.e., deforestation exceeds afforestation in these regions by 2050 in the absence of novel CDR). An interesting observation can also be seen in Fig. 5g-i, which represents the year when each Asian country/region achieves net-zero CO_2 emissions (irrespective of them collectively reaching net-zero GHG emissions by mid-century, as the modeled pathway stipulates). Under the HIGH scenario, Southeast Asia would attain net-zero CO_2 emissions the earliest, before 2040, while countries like Central Asia, China, India, and South Asia achieve theirs before 2045, followed by Japan before 2050. The rest of the countries or regions (Indonesia, the Middle East, Pakistan, South Korea, and Taiwan (China) rather pursue cheaper decarbonization routes by purchasing foreign CDR instead of achieving net zero before the end of 2050, which is relatively more expensive. However, under the MODERATE and LOW scenarios, Asian countries or regions could advance or maintain the year of attaining net-zero CO_2 emissions compared to the case of the HIGH scenario. Interestingly, it becomes relatively cheaper to pursue net zero before the end of 2050 under the MODERATE and LOW scenarios than to purchase foreign CDR. Hence, all countries/regions would achieve net-zero CO_2 before 2050 under the MODERATE (except South Korea) and LOW scenarios.

Supplementary Discussion 3: Impact on land, water, and fertilizer consumption

There are potential trade-offs concerning water consumption associated with different levels of CDR reliance. As seen in Fig. 6b, high CDR reliance could risk increasing water demands in sectors or for activities such as bioelectricity CCS, bioenergy crop cultivation, CO_2 removal, industry, meat and dairy, municipal water, and non-food crop cultivation. Due to the deployment of 4.6 $GtCO_2$ of BECCS by mid-century under the HIGH scenario compared to 1.9 $GtCO_2$ in the MODERATE scenario, about 3.6 km^3 of water is consumed for bioelectricity CCS by 2050 under the HIGH scenario compared to 1.7 km^3 under the MODERATE scenario. Results from the previous sections show an increased affinity for electricity consumption in final energy under the MODERATE and LOW scenarios. As such, water consumption for

electricity generation would reach 76-80 km^3 under the MODERATE and LOW scenarios by 2050 compared to 64 km^3 under the HIGH scenario. Furthermore, despite having a relatively higher land allocation to bioenergy crop cultivation, the water consumption for growing bioenergy crops remains lower under the MODERATE and LOW scenarios compared to that under the HIGH scenario. This could be attributed to the nature of crops grown purposely for bioenergy in the three scenarios. The results indicate that due to its lower price pathway, the bioenergy crops under the HIGH scenario have a significantly higher biophysical water footprint and also possess additional requirements for artificial irrigation beyond what is required for bioenergy crops under the MODERATE and LOW scenarios.

By 2050, about 15 km^3 of water would be consumed by DACCS for removing CO_2 from the atmosphere under the HIGH scenario, which is completely avoided in the MODERATE and LOW scenarios. Also, due to the higher land allocation for food crops under the MODERATE scenario, the water consumption for cultivating food crops by 2050 would be 5% higher than that consumed under the HIGH scenario. On the other hand, the heavy industrial processes coupled with high fossil fuel consumption and inefficient practices under the HIGH scenario would cause a 13% higher water consumption in the industry sector by 2050 compared to that consumed under the MODERATE scenario.

Climate ambitions would increase fertilizer demand [25] primarily due to the increased requirement for bioenergy as shown in Fig. 6c. In the REFERENCE scenario, the requirement for nitrogen fertilizer is almost the same as that required in the net-zero pathways, at approximately 85 million tonnes of Nitrogen (MtN) by 2050. However, under the REFERENCE scenario, only 2 MtN of nitrogen fertilizer would be required for cultivating bioenergy crops by 2050, compared to about 7 MtN in the net-zero pathways. The demand for nitrogen fertilizer across different levels of CDR reliance is similar to the observation made in the results of water consumption. The HIGH scenario would lead to a slightly higher fertilizer demand for bioenergy crop cultivation (7 MtN by 2050) compared to 6.6 MtN and 6.8 MtN in the MODERATE and LOW scenarios, respectively.

Ambitious climate goals in Asia will lead to land-use changes in the region. The HIGH CDR scenario would allocate more land annually to bioenergy crop cultivation than the LOW and MODERATE scenarios would. Interestingly, croplands would receive slightly more allocation under HIGH CDR. This could be attributed to the LOW/MODERATE CDR pathways' higher allocation of agro-land use such as grass, other arable, pasture, and shrubs (more details in Supplementary Table 5). Fig. 7 highlights land allocation towards bioenergy crops and other agro-land allocation (crops, grass, other arable, pasture, and shrubs) among Asian countries and regions in response to varying CDR reliance from 2025 to 2050. We find that while HIGH CDR has higher annual land allocation towards bioenergy crop cultivation, the change in this land use type over the next three decades among Asian countries and regions is significantly higher in LOW CDR compared to HIGH and MODERATE CDR scenarios. Bioenergy consumption (without CCS), similar to other zero-carbon energy sources, increases under limited reliance on CDR. As such, land allocation for bioenergy crop cultivation under the LOW scenario will expand significantly between 2025 and 2050 among Asian countries and regions (Fig. 7a-c). In all pathways, South Korea exhibits the highest change in land allocation for cultivating bioenergy crops at over 3000% increase under LOW CDR compared to less than 1500% under HIGH CDR. Taiwan (China) exhibits the lowest changes in bioenergy cropland allocation especially under MODERATE CDR at a 50% increase. Aggregated land allocation for agro-land use types such as crops, grass, shrubs, and pasture witness decreasing growth rates over the next three decades across all scenarios. The reduction rates are least severe under the MODERATE CDR pathway compared to HIGH and LOW CDR scenarios. Under higher expectations for CDR, the most severe change in agro-land use (excluding biomass) would be recorded in Japan at -10.5% followed by Indonesia (-8.4%), and Pakistan (-7.7%). Similarly, the least affected country/region under this pathway would be South Korea (-1.9), followed by South Asia (-2%), and Central America (-2.6%) (Fig. 7d-f).

Supplementary Discussion 4: Validation

The existing data for validating our results corresponds to the R5ASIA dataset[26]. The R5ASIA dataset includes approximately 550 scenarios. However, we only focus on scenarios that explicitly outline pathways in alignment with the global 1.5°C target, maintaining consistency with our pathways.

The discrepancies in some indicators between our results and those from existing scenarios (Supplementary Table 6) can mainly be attributed to what Dekker et al. [27] referred to as 'energy model fingerprints.' These fingerprints describe how models differ in structure, objectives, assumptions, parameterization, and level of detail. These differences result in variations in the computed energy and climate policy scenarios [27,28]. Another reason could be how we categorize countries/regions under 'Asia' in our pathways compared to existing scenarios. For instance, the R5ASIA scenario does not include Japan in its scope (See Supplementary Table 7 for a complete list of Asian countries/regions under Global Change Assessment Model-Tianjin University (GCAM-TJU) and that of R5ASIA). Additionally, the variation in mitigation pathways between our scenarios and the existing ones can significantly affect key modeling results. To illustrate, our pathway is one in which Asia collectively achieves net zero GHG emissions around mid-century. In contrast, the existing scenarios usually follow a net-zero CO_2 pathway that typically supports a global net-zero by 2050, in line with the 1.5°C target. Consequently, the level of stringency in our pathways is of a higher magnitude than in the existing scenarios.

Supplementary Table 7 Countries and regions for Asia under R5ASIA and our GCAM-TJU

Source	Countries/Regions
GCAM-TJU	Armenia, Azerbaijan, Georgia, Kazakhstan, Kyrgyzstan, Mongolia, Tajikistan, Turkmenistan, Uzbekistan, China, India, Indonesia, Japan, United Arab Emirates, Bahrain, Iran, Iraq, Israel, Jordan, Kuwait, Lebanon, Oman, Palestine, Qatar, Saudi Arabia, Syria, Yemen, Pakistan, Afghanistan, Bangladesh, Bhutan, Sri Lanka, Maldives, Nepal, South Korea American Samoa, Brunei Darussalam, Cocos (Keeling) Islands, Cook Islands, Christmas Island, Fiji, Federated States of Micronesia, Guam, Cambodia, Kiribati, Laos, Marshall Islands, Myanmar, Northern Mariana Islands, Malaysia, Mayotte, New Caledonia, Norfolk Island, Niue, Nauru, Pacific Islands Trust Territory, Pitcairn Islands, Philippines, Palau, Papua New Guinea, Democratic Peoples Republic of Korea, French Polynesia

	Singapore, Solomon Islands, Seychelles, Thailand, Tokelau, Timor-Leste, Tonga, Tuvalu, Vietnam, Vanuatu, Samoa, Taiwan (China)
R5ASIA [26]	China, China Hong Kong SAR, China Macao SAR, Mongolia, Taiwan (China), Afghanistan, Bangladesh, Bhutan, India, Maldives, Nepal, Pakistan, Sri Lanka, Brunei Darussalam, Cambodia, Democratic People's Republic of Korea, East Timor, Indonesia, Lao People's Democratic Republic, Malaysia, Myanmar, Papua New Guinea, Philippines, Republic of Korea, Singapore, Thailand, Viet Nam

GCAM-TJU here refers to our modified version of the GCAM tool. TJU represents the abbreviation of the affiliation (Tianjin University) of the first and corresponding authors. R5ASIA is a dataset from the Intergovernmental Panel on Climate Change (IPCC) Sixth Assessment Report (AR6)[26]. The dataset contains AR6-assessed scenarios for the Asian regions mentioned in Supplementary Table 7 for "R5ASIA". SAR: Special Administrative Region.

Supplementary Discussion 5: Sensitivity analysis

To validate the robustness of our initial findings, we conducted a sensitivity analysis that involves varying key modeling parameters. This analysis includes four distinct modeling parameters, i.e., biochar application and sequestration rates, the proportion of GDP allocated to the negative emissions budget, and the role of land use change in climate mitigation. Our objective is to assess the degree to which each parameter impacts our initial results in terms of energy-land-water-fertilizer demand, mitigation costs, positive and negative emissions.

The biochar application rate, measured in tons per hectare, indicates the quantity of biochar applied to cropland areas in a single instance during the modeled years [29]. For clarity, if the application rate is 10 tons per hectare (tha^{-1}), 10 tons of biochar are applied to each hectare one time only from 2025 (the first year in our modeling period) to 2050 (the last year in our modeling period). Existing literature typically estimates biochar application rates within 10-100 tha^{-1} [29–31]. Our primary scenarios employ an application rate of 20 tha^{-1}. However, to ensure the robustness of our findings, we also consider application rates of 10 tha^{-1} and 100 tha^{-1}.

The sequestration rate denotes the proportion of the carbon content in biochar that effectively contributes to long-term carbon sequestration in soil, with a centennial scale perspective [29]. Biochar can exhibit

sequestration rates as high as 97% [32,33], but we have adopted a conservative 70% sequestration rate in our primary scenarios. We also included sequestration rates of 50% and 90% in our sensitivity analysis. This allows us to assess how the initial results are influenced by variations in the carbon sequestration rate of biochar.

The discount rate is an important factor to consider in climate mitigation scenarios, particularly those generated by IAMs. In climate change and IAMs, the discount rate refers to the rate at which future costs and benefits are discounted or given less weight compared to present costs and benefits [8]. Most IAMs commonly employ a discount rate of 3% to 5%. However, there are alternative rates within this range and beyond. Previous studies, such as those conducted by Riahi et al.[34], Fuhrman et al. [1] and Emmerling et al. [35], demonstrated that lower discount rates tend to favor earlier mitigation actions, reducing the reliance on CDR technologies and temperature overshoot. Essentially, a lower discount rate emphasizes taking immediate steps to mitigate climate change, resulting in higher initial transition costs. Nevertheless, this approach yields long-term economic benefits and earlier advantages in avoiding climate change impacts. Conversely, higher discount rates tend to postpone mitigation efforts, leading to delayed transition pathways.

In Global Change Assessment Model (GCAM), the standard discount rate used is either 3% or 5% (as used in this study). The discount rate represents the exogenous escalation in the price of CO_2 or GHG over time, either within a scenario with an end-of-century target or one with an exogenous carbon pricing trajectory [36]. Investigating the role of discount rate in our mitigation pathways is beyond the scope of this study. Our objective is not to evaluate the intertemporally "optimal" decision-maker, but rather to simulate imperfect decision-making. In our case, where constraints are set on GHG emissions (as opposed to scenarios with fixed end-of-century climate target [1,25,37]), the model simply needs to align with the pre-determined emissions constraint for each period regardless of the chosen discount rate. That is, we do not allow the flexibility of optimally delaying mitigation based on high discount rate.

While the influence of discount rates on climate mitigation is well-documented [1,34,35], the impact of the negative emissions budget has received considerably less attention within the scientific community. The

negative emissions budget represents the portion of GDP allocated for financial transfers related to negative emissions. In alignment with our primary research objective, we adjust this negative emissions budget from 0.25% to 1%. This variation allows us to assess the degree of influence it exerts on regional energy-land-water system under varying levels of reliance on CDR technologies.

The current study also investigates the impact of land use change on climate mitigation. In our constrained GHG emissions pathway, the emission constraint applies explicitly to carbon emissions from fossil fuels and industrial processes. Consequently, net emissions resulting from land use change, including afforestation/reforestation and deforestation, are not subject to this constraint. However, we have implemented a pricing mechanism for carbon emissions associated with land use, gradually increasing from 10% to 100% of the carbon price applied to fossil emissions, from 2025 to 2100. This approach serves a dual purpose. Firstly, it facilitates additional reductions in emissions, contributing to mitigation efforts. Secondly, it incentivizes carbon storage through land use changes, mainly through afforestation and reforestation practices.[38]. In the main scenarios, the carbon price on land use sector reaches 40% by 2050, representing high mitigation in the land use sector. For sensitivity analysis, we have introduced two additional mitigation roles for the land use sector. These roles consist of a mid-range role, where a constant carbon price of 30% is applied to the land use sector from 2025 to 2050, and a lower role, where a constant carbon price of 10% is applied to the land use sector over the same period.

It is important to highlight that the testing of biochar application and sequestration rates is carried out on the HIGH scenario since it is the only scenario that features biochar as a CDR option, while the examination of the negative emissions budget and the role of land use in mitigation is conducted within the MODERATE scenario. This scenario is chosen because it represents a middle ground, offering insights into the moderate challenges and regional impacts between decarbonization and carbon removal.

Our analysis indicates that, for the most part, the biochar application rate (Supplementary Fig. 22), biochar sequestration rate (Supplementary Fig. 3), and the allocation of GDP towards negative emissions (Supplementary Fig. 4) have minimal impact on our initial results. However, cropland allocation is highly

sensitive to changes in the biochar application rate. By 2050, in a scenario with a biochar application rate of 10 tha^{-1}, cropland allocation without biochar reaches 1.1 Mkm^2 compared to 1.3 Mkm^2 and 2.5 Mkm^2 in scenarios with application rates of 20 tha^{-1} and 100 tha^{-1}, respectively (Supplementary Fig. 22a). Similarly, cropland allocation is strongly influenced by changes in the carbon sequestration rate of biochar. Higher removal rates lead to increased biochar cropland allocation, which in turn reduces land allocation to croplands (i.e., cropland allocation without biochar). For example, at a removal rate of 90%, biochar demand reaches 4365 million tonnes (Mt), compared to 4200 Mt and 4000 Mt at sequestration rates of 70% and 50%, respectively. Consequently, cropland allocation without biochar by 2050 in a scenario with a biochar sequestration rate of 90% would reach 1.1 Mkm^2 compared to 1.3 Mkm^2 and 1.5 Mkm^2 in scenarios with sequestration rates of 70% and 50%, respectively (Supplementary Fig. 3a).

The role of negative emissions from land-use change has the most significant impact on our initial results (Supplementary Fig. 23). In the 10% carbon price scenario (Supplementary Fig. 23a), cropland allocation remains the highest at 4.14 Mkm^2 compared to 4.12 Mkm^2 at 30% and 4.10 Mkm^2 at 40% carbon prices. Since land use change has a limited role in offsetting residual emissions at a 10% carbon price, electrification, energy extraction, and biomass consumption would need to increase to compensate. Consequently, a 10% constant carbon price in the land use sector would lead to higher water consumption for negative emissions and energy generation compared to consumption at 30% and 40% (Supplementary Fig. 23b). Additionally, the increased biomass consumption and cropland allocation in the 10% carbon price scenario would result in higher demands for total nitrogen fertilizer (Supplementary Fig. 23c). Biomass, as a primary energy source, would need to increase significantly to compensate for the lower negative emissions from land use change under a 10% constant carbon price (Supplementary Fig. 23d). As a result of its substantial biomass demand, the consumption of renewables and nuclear energy tends to decrease slightly at a 10% carbon price compared with higher carbon prices in the land use sector (Supplementary Fig. 23e). Furthermore, a less stringent climate pathway in a lower carbon price in the land use sector would

lead to the highest residual GHG emissions (Supplementary Fig. 23g), lower gross CDR allocation (Supplementary Fig. 23h), and lower marginal cost (Supplementary Fig. 23i).

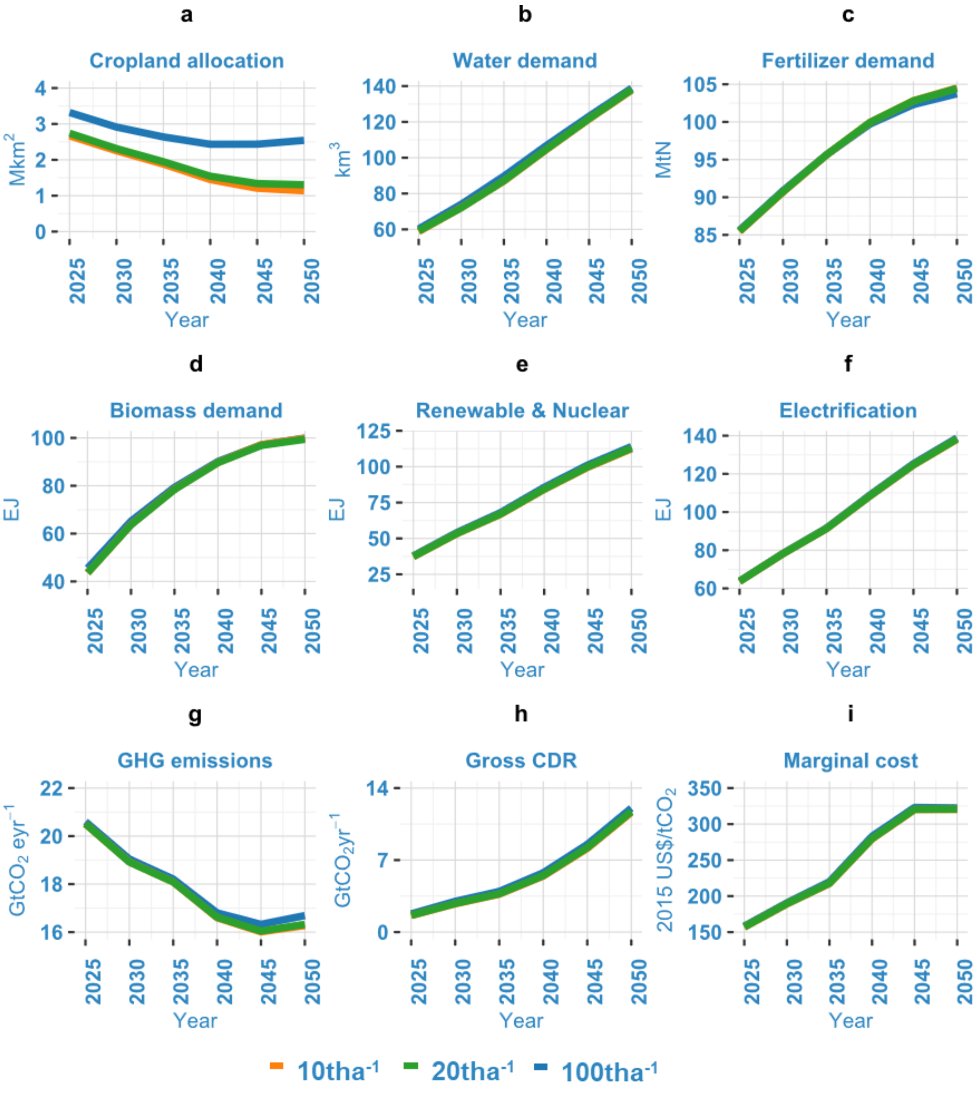

Supplementary Figure 22 Influence of biochar application rate (10-100 tha^{-1}). Impacts on cropland allocation without biochar (a) water demand (b) fertilizer demand (c) biomass demand (d) renewable and nuclear deployment (e) electricity consumption in end-use sectors (f) GHG emissions (g) gross CDR (h) carbon price (i). Since biochar, as a CDR approach, is only considered in our HIGH scenario, the sensitivity analysis was exclusively conducted on this particular scenario. Water demand represents water consumption for negative emissions and energy generation. $GtCO_2eyr^{-1}$: gigatonnes of carbon dioxide equivalent per year; EJ: Exajoule; $\$/tCO_2$: United States Dollar per ton of carbon dioxide; Mkm^2: million square kilometers; km^3: cubic kilometers; MtN: million tonnes of nitrogen; CDR: carbon dioxide removal; GHG: greenhouse gas; tha^{-1}: tonnes per hectare

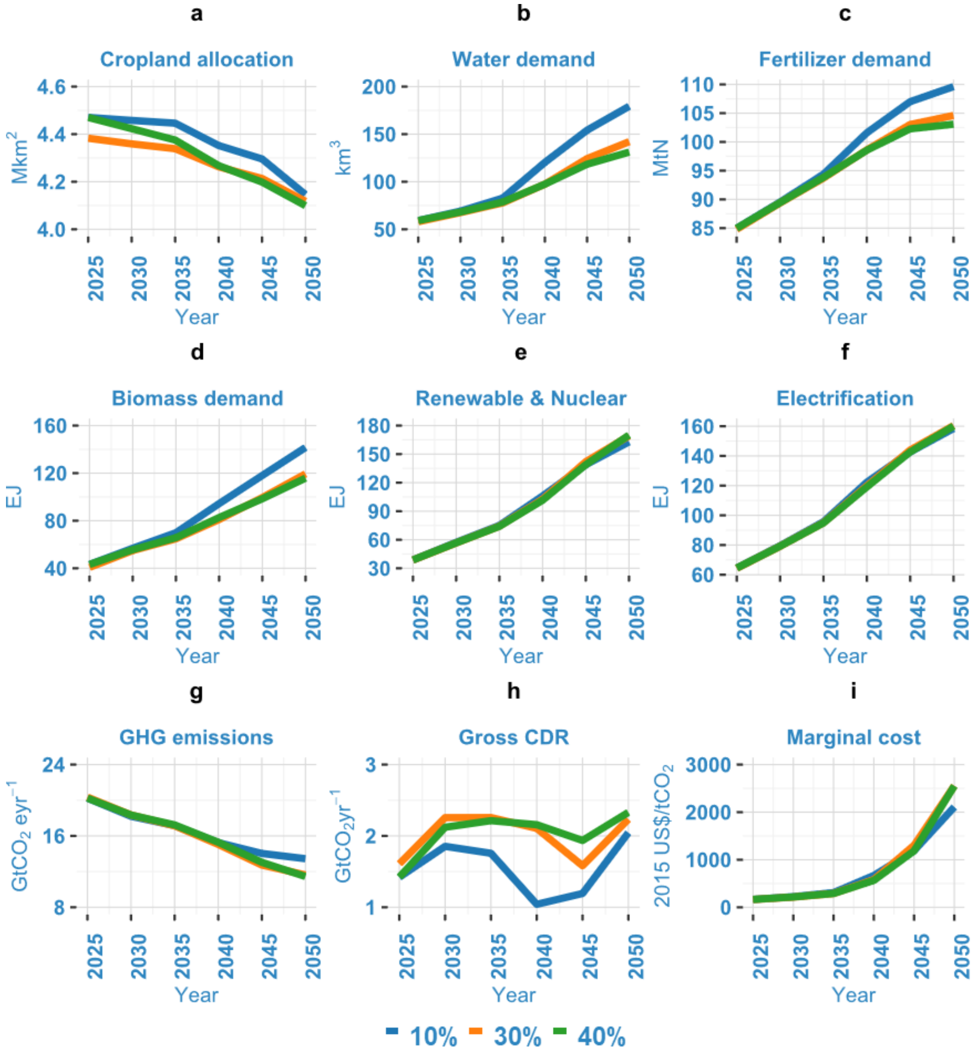

Supplementary Figure 23 Influence of land use sector involvement in climate mitigation. Impacts on cropland allocation (a) water demand (b) fertilizer demand (c) biomass demand (d) renewable and nuclear deployment (e) electricity consumption in end-use sectors (f) GHG emissions (g) gross CDR (h) carbon price (i). The sensitivity analysis here is based on our MODERATE scenario, since this scenario represents moderate challenges and regional impacts between decarbonization and carbon removal. Water demand represents water consumption for negative emissions and energy generation. 'LUC' means 'land use change'. 10-40% involvement represents the fraction of fossil fuel and industry emissions by 2050 that is used to price emissions from land use change. $GtCO_2eyr^{-1}$: gigatonnes of carbon dioxide equivalent per year; EJ: Exajoule; $\$/tCO_2$: United States Dollar per ton of carbon dioxide; Mkm^2: million square kilometers; km^3: cubic kilometers; MtN: million tonnes of nitrogen; CDR: carbon dioxide removal; GHG: greenhouse gas

Supplementary Note 1: Estimating capital stock turnover

To quantify the annual capacity additions, stranded assets, and associated capital investments in the electricity sector, we employ the approach outlined by Ou et al. [39] described as follows. GCAM tracks power plant capital stock, technology type, and vintage across the entire lifetime of each technology. The retirement of power plants can occur through two distinct pathways: 1) Natural retirement upon reaching the end of their designed physical lifetime, or 2) Profit-induced premature retirement when the continued operation of plants becomes less profitable, resulting in stranded assets. The calculations for capacity and capital stock turnover in the electric power sector were done using Plutus package in R [40], which follows a 3-step process summarized below [39,41]. Based on each country's output from our scenarios, the electricity generation by technology and vintage is extracted. In step 2, the natural retirement fraction for each vintage is estimated for each new fleet. Our scenario electricity generation output and the "expected" natural retirement trajectory from step 2 are compared to estimate the premature retirement and stranded assets. The product of premature retirement and the corresponding capital cost of each technology is used in calculating stranded assets [39,41]. Capital cost assumptions for the electric power sector and the physical lifetime assumptions for technologies in the electric power sector are provided in Supplementary Note 2 (Supplementary Table 8 and Supplementary Table 9, respectively).

Supplementary Note 2: Land use change as a carbon dioxide removal method in GCAM

GCAM tracks carbon flows between the atmosphere, biosphere, and anthroposphere resulting from land-use change (e.g., forest or grassland conversion to cropland). Under a carbon price policy, GCAM imposes a cost penalty on land-use change emissions and provides a subsidy for land-use carbon sequestration (e.g., expanding forest area). While fossil fuel emissions can theoretically be priced at the source, land-use change and agricultural emissions currently lack regulatory frameworks or mature market

infrastructure. Realizing the full climate benefits of biospheric carbon management will require developing governance and pricing mechanisms for these diffuse emissions and sinks [42].

GCAM applies a carbon price to all anthropogenic carbon flows, despite the lack of existing policy levers for biospheric carbon. To better reflect real-world constraints, we implement a separate carbon price trajectory for land-use change emissions, starting at 10% in 2025 and linearly approaching the fossil fuel carbon price by 2100. This gradual convergence represents the long-term effort needed to implement land-use policies and infrastructure that address current barriers, including non-permanence risks of biospheric carbon storage. For sensitivity analysis, we have introduced two additional mitigation roles for the land use sector. These roles consist of a mid-range role, where a constant carbon price of 30% is applied to the land use sector from 2025 to 2050, and a low role, where a constant carbon price of 10% is applied to the land use sector over the same period.

Supplementary Note 3: BECCS as a carbon dioxide removal method in GCAM

GCAM includes bioenergy with carbon capture and storage (BECCS) technologies in the refining, electricity generation, hydrogen production and industrial sectors. The land, water, and fertilizer requirements for bioenergy supply and afforestation/reforestation are resolved endogenously in GCAM among 384 land-use regions. These regions represent the intersection of 32 geopolitical regions and 235 water basins. This accounts for the interactions with food crop production and natural lands [22]. The parameterization of all biomass energy technologies, including BECCS, is based on previous GCAM studies [43–45]. In GCAM, land area allocated to biomass competes with other uses including cropland and natural lands. GCAM endogenously solves for irrigation water utilization (rainfed versus irrigated crops) and fertilizer application for biomass and all other agricultural commodities in each major river basin. It accounts for the yield effects of irrigation, fertilization, and any emissions tax or carbon sequestration subsidy [42].

Supplementary Note 4: DACCS as a carbon dioxide removal method in GCAM

Supplementary Table 19 reports the parameterizations used for the direct air carbon capture and storage (DACCS) technologies in this study. We generally followed the detailed methodology of Fasihi et al. [46], adjusting the financial discount rate assumptions to derive more conservative early cost estimates for these emerging technologies. For low-temperature DACCS, we converted the required low-temperature thermal energy to electricity assuming an electric compression heat pump with a coefficient of performance of 3. We also accounted for the additional electricity required to compress the captured CO_2 to pressures suitable for subsurface injection. To better inform near-term deployment potential, we assumed cost and energy efficiency improvements over the next decade (by 2030) that remain constant thereafter. The 1.6- to 4-fold non-energy cost reductions for DACCS over 10 years are partly due to our initially conservative estimates but are within historical improvement rates for solar photovoltaics, batteries, and other mitigation technologies [47]. Given minimal biophysical constraints on global DACCS scaling, even our lower bound estimates of financial and energetic inputs represent relatively conservative technology advancement projections compared to other literature [46,48].

Supplementary Note 5: DORCS as a carbon dioxide removal method in GCAM

Direct ocean removal with carbon storage (DORCS) leverages the higher effective CO_2 concentration in seawater, exceeding that in the atmosphere by approximately a factor of 120 due to dissolved inorganic carbon [49–51]. Electrochemical processes shift the pH to off-gas CO_2. When this aqueous CO_2 is sequestered in geologic reservoirs, the discharged seawater absorbs more atmospheric CO_2 to restore equilibrium [52,53]. Existing techno-economic studies indicate DORCS costs per tCO_2 far exceed DACCS, owing to the energy penalty and infrastructure costs of seawater intake and outfall, which can dwarf the electrodialysis system itself [54]. Co-locating DORCS with desalination plants could substantially reduce costs [52,55].

We considered two DORCS technologies, both parameterized from Digdaya et al. [52]. One is a stand-alone system structurally analogous to DACCS in GCAM, with unique cost and performance. The second, much less electricity-intensive system captures CO_2 as a desalination co-product. It competes with other desalination methods, deploying when the CO_2 capture subsidy (equal to the emissions price) exceeds the additional non-energy and electricity costs. Supplementary Table 22-Supplementary Table 25 provides modelling assumptions for DORCS.

Supplementary Note 6: Biochar as a carbon dioxide removal method in GCAM

Our biochar deployment in GCAM is based on the development of Bergero et al.[29]. Biochar application to croplands can increase recalcitrant carbon stocks in soils [22]. Biochar is produced by pyrolyzing biomass feedstocks under anoxic conditions, rendering the remaining photosynthetically fixed carbon resistant to reoxidation and enabling centennial-scale carbon storage [56]. This gives biochar unique potential compared to other soil carbon sequestration pathways with lower scalability and permanence [57]. Like enhanced weathering amendments, biochar may increase yields and reduce water and fertilizer needs, depending on climate zone and soil type [30,58–60]. Biochar production requires energy input but can co-produce useful energy such as biogas, albeit at the expense of lower net removal efficiency [61–63]. It can also be a co-product of biomass electricity generation [64]. In GCAM, biochar production is modeled from lignocellulosic biomass pyrolysis with syngas co-production. Competition between direct biomass use for energy (including BECCS) and biochar is endogenously determined in GCAM. Biochar is then an input option to improve crop yields, similar to fertilizer and irrigation [65,66]. In our primary scenarios, we employ an application rate of 20 tha^{-1}. However, to ensure the robustness of our findings, we also consider application rates of 10 tha^{-1} and 100 tha^{-1}. Biochar can exhibit sequestration rates as high as 97% [32,33], but in our primary scenarios, we have adopted a conservative 70% sequestration rate. As part of our sensitivity analysis, we have also included sequestration rates of 50% and 90%. Supplementary Table 26 represents biochar demand and supply assumptions (based on Ref. [29]).

Supplementary Note 7: ERW as a carbon dioxide removal method in GCAM

Enhanced rock weathering (ERW) accelerates natural processes that regulate Earth's carbon cycle over geological timescales [67–69]. Crushing calcium- or magnesium-bearing minerals increases their surface area, dramatically enhancing the reaction rate of atmospheric CO_2 into bicarbonate ions [70–72]. ERW has been demonstrated in lab experiments, small field trials, and idealized models [73–76]. Compared to DACCS, ERW may have lower removal costs per tonne CO_2 even considering crushing and transport energy inputs [46,77–79]. ERW poses less competition for land and water versus BECCS or afforestation/reforestation, as it can occur on already perturbed lands (e.g. cropland, managed forests/grasslands). Alkalinity addition may even boost crop yields and reduce irrigation needs in some climates and soils [80,81]. A portion of the bicarbonate precipitates into soils, while the remainder flows to oceans, ameliorating acidification [82]. However, dissolution along the land-aquatic-ocean continuum may reduce ERW efficacy [83]. Large material requirements may also cause local environmental damage from extraction, transport, crushing, and spreading [84]. We considered basalt ERW on croplands, using country-level potentials from Beerling et al. [71] to develop regional supply curves in GCAM. For missing regions, we scaled the aggregated curve linearly by regional potentials from Strefler et al. [77]. GCAM endogenously calculates regional electricity costs and carbon intensity, so we subtracted the fixed cost assumption from Strefler et al. [77] (Supplementary Supplementary Table 20). We assumed an electricity input declining from their 2020 upper bound of 2 Gigajoule per tonne of CO_2 (GJ per tCO_2) to their 2050 best estimate of 0.66 GJ per tCO_2 (Supplementary Supplementary Table 21). We also subtracted rock transport costs, with freight mode, fuel mix, and cost determined endogenously in GCAM. This leaves regional non-fuel cost supply curves. Assuming 300 km average transport and 0.3 tCO_2 per rock from Strefler et al. [77] gives a freight input of 1000 tonne-km per tCO_2 removed. This approach yields regional cost curves distinct from GCAM's existing geologic carbon disposal. For conservative 2020 estimates, each curve was shifted up by the "upper bound" versus "best

estimate" differential in investment and operations/maintenance costs from Strefler et al. [77] (\$138 per tCO_2). This adder declines to zero by 2050, leaving just the regional supply curves.

Supplementary Note 8: GCAM's land module

In GCAM, economic land use decisions are based on a logit model sharing approach according to relative inherent profitability of competing purposes. This represents distributions of profit behind each land use rather than point estimates, with higher average profits conferring higher shares. At the margin, profits equalize across uses so allocation is optimal, unlike constrained linear optimization.

Land uses are nested within nodes, with higher logit exponents indicating tighter competition (e.g. substituting crops). Lower exponents govern harder substitution across nodes (e.g. cropland expansion into pasture). Although relative average profits determine shares, equal marginal profit rates across uses mean marginal land values equalize. If one use has higher average profit, its profit distribution has greater density exceeding the marginal rate, conferring higher share, unlike simple optimization. The nonlinear logit approach produces diminishing returns as land uses diverge from history, obviating explicit constraints. This contrasts linear models with constant returns. Logit exponents relate to land use change elasticities. See Wise et al. [85] for further discussion.

The nesting strategy for competing land uses and substitution exponents (logit exponents) governing each nest involve expert judgement [86]. In standard runs, historical calibration does not depend on this structure or exponents. Instead, they affect future projections as conditions diverge from history. At one extreme is a single nest with high exponent, representing completely unconstrained optimization and easy switching between any land uses. The other extreme is near-zero exponents, disallowing substitution. We employ positive exponents for economics-driven but constrained allocation. Our nesting approach captures varying substitutability across land categories. The hierarchy begins with total land, divided into agro-forestry and non-agricultural. The latter can subdivide further if needed. Agro-forestry comprises pasture and non-pasture nodes. Pasture includes managed grazing and unmanaged land. Non-pasture consists of

shrub/grassland, forest, and cropland nodes, with forests competing against total croplands. Forests include managed, unmanaged, and woody biomass. Croplands encompass food, feed, fiber, and dedicated energy crops, plus fallow land. Crop areas further subdivide into irrigated/rainfed and high/low management. This structure balances model flexibility with computational constraints. The exponents aim to represent real-world frictions and transition costs limiting easy switching between all land uses.

The profit-based logit land sharing must be calibrated to match historical land use shares and profit rates. Conceptually, calibration infers distributions and parameters from data, given the chosen model structure. Based on the nesting, exponents, and base year data, the observed profit rates imply underlying economic land values. The approach is to solve for parameters that adjust observed profits to equal the potential average profits implied by base year shares and unmanaged land price. These implied rates represent average profit if all land went to that use; we refer to them as calibration profit rates. The resulting parameters (calibration profit scalers) rescale future projected profits in the logit sharing and profit equations. This grounds the future in history - with unchanged future conditions, shares and allocations reproduce the base year. As model dynamics shift future profits from historical values, shares also evolve. The absolute scaler values have meaning, unlike relative share weights. The calibration routine ensures internally consistent economics given the model structure, while allowing land use responses to diverging future conditions.

The equations that determine land allocation and the resulting carbon emissions from land-use and land-cover changes are described here.

The profit for managed land leafs is calculated in the supply module and passed to the land allocator. Profit for unmanaged land leafs is input into the model. Within the land allocator, profit is adjusted if land-related policies are included. Managed land leafs here generally refers to land areas that are under direct human management or influence. This can include agricultural lands, forest plantations, urban areas, and other land uses where human activities directly impact land cover and land use patterns.

The average profit of a node is calculated as (Supplementary Equation 1):

$$\pi_i = \left[\sum_{j=1}^{N} \lambda_j^{\rho} \pi_j^{\rho}\right]^{\frac{1}{\rho}} \tag{1}$$

Where λ_i is the profit scaler for leaf or node i, π_i is the profit for node i, π_j is the profit for leaf or node j contained within node i, and ρ is the logit exponent.

The share of each leaf or node is calculated as (Supplementary Equation 2):

$$s_i = \frac{(\lambda_i \pi_i)^{\rho}}{\sum_{j=1}^{N} (\lambda_j \pi_j)^{\rho}} \tag{2}$$

Where s_i is the share of leaf or node i, λ_i is the profit scaler for leaf or node i, π_i is the profit for leaf or node i, and i, ρ is the logit exponent.

To calculate land area, GCAM works its way down the nesting tree, starting from the top where the total land area in a region is provided as an input. For each node or leaf below, the area was calculated as (Supplementary Equation 3):

$$a_i = a_{above} * s_i \tag{3}$$

where a_i is the area for leaf or node i, s_i is the share, and a_{above} is the area of the parent node.

The total cumulative change in emissions is calculated as (Supplementary Equation 4):

$$E_t^{veg/soil} = \Delta C_t = A_t * D_t^{veg/soil} - A_{t-1} * D_{t-1}^{veg/soil} \tag{4}$$

where E indicates carbon emissions due to a land use change in time step t, C indicates carbon stocks, A indicates land area, and D indicates the average carbon density of the land area. These emissions are allocated differently over time for vegetation and soil carbon.

If vegetation emissions are positive (i.e., $E_t^{veg} > 0$), then all emissions are released in the current year y. That is, $E_y^{veg} = E_t^{veg}$.

If vegetation emissions are negative, then these emissions are spread over time using a sigmoid function (Supplementary Equation 5):

$$E_y^{veg} = E_t^{veg} * \left[1 - e^{\frac{-3.0*(y-t+1)}{M}}\right]^2 - \left[1 - e^{\frac{-3.0*(y-t)}{M}}\right]^2 \quad (5)$$

where t is the time of land conversion, y is the current year, and M is the mature age (specified by land type and region).

Soil carbon emissions followed an exponential approach (Supplementary Equation 6):

$$E_y^{soil} = E_t^{soil} * \left[\left(1.0 - e^{-1.0*\kappa*(y-t)}\right) - \left(1.0 - e^{-1.0*\kappa*(y-t-1)}\right)\right] \quad (6)$$

where $\kappa = \frac{\log(2)}{s/10.0}$ and s is the soil time scale, specified by region.

Total carbon stock, C_y in year, y is calculated as (Supplementary Equation 7):

$$C_y = C_{y-1} - \left[E_{y-1}^{veg} + E_{y-1}^{soil}\right] \quad (7)$$

where E_y^{veg} are vegetation carbon emissions in year y and E_y^{soil} are soil carbon emissions in year y.

Supplementary Note 9: GCAM's water module

Water demand is calculated for six major sectors: agriculture, electricity generation, industrial manufacturing, primary energy production, livestock, and municipal uses.

Agriculture water demand is determined by exogenous water coefficients and endogenous crop production. For irrigated crops, GCAM tracks water withdrawals, consumption, and biophysical consumption. Withdrawals include crop evapotranspiration met by irrigation ("blue water") and field losses. Conveyance losses are represented in water distribution sectors. Loss coefficients are from Rohwer et al. [87]. Consumption

is the blue water evapotranspiration only, excluding rainfall ("green water"). Biophysical consumption sums blue and green water needs, excluding losses. It applies to both rainfed and irrigated technologies. More documentation is available in Chaturvedi et al. [88] and Hejazi et al. [89]. The model accounts for different agricultural water flows and losses to represent water resource competition and agricultural production.

GCAM represents up to five cooling options for each thermal power technology: once-through, recirculating, pond, dry, and seawater once-through. These differ significantly in water withdrawal and consumption per National Energy Technology Laboratory (NETL) [90]. Technology-specific demand coefficients are from Macknick et al. [91]. Not all options are available in each region based on underlying data; for example, cooling ponds are often excluded. Currently there is no dry cooling option for nuclear plants. Cooling systems compete in a calibrated logit nest, like fuels. Capital costs vary per NETL [90]. Dry cooling has lower generation efficiency. Competition is endogenous and cost-based. Further documentation is in Davies et al. [92] and Kyle et al. [93]. The model captures different power plant cooling technologies and associated water demands to represent electricity-water interlinkages.

The industrial manufacturing sector’s water demands include surface and groundwater that is self-supplied by industrial manufacturers. The water demanded by this sector excludes water demands of coal mining and oil and gas production, which are represented in the respective energy production sectors. It also excludes water withdrawn for cooling of on-site (i.e., located at industrial facilities) thermo-electric power generation, which is modeled in the electricity generation sector. Finally, industrial facilities’ use of municipal water is also excluded (modeled in the municipal water sector).

All animal production technologies in GCAM have region-specific water demand coefficients representing drinking water and operations. There is no withdrawal/consumption distinction; all water is consumed. Coefficients are in cubic meters per kg commodity produced. They are calculated from Mekonnen and Hoekstra [94], which provides liters per animal per day by country around 2000. Computation also considers animal stocks from Food and Agriculture Organization Corporate Statistical Database (FAOSTAT) [95]. The resulting demand coefficients are held constant over time. This approach captures livestock water needs

and links them to regional production levels. The coefficients integrate data on water use per animal and animal inventories to represent total water demands.

The approach for modeling the water demands of primary energy production is documented in Hejazi et al. [89], and includes bottom-up estimates of water demand per unit energy produced for the following fuels: coal, oil (conventional and unconventional), natural gas, and uranium. The main data source used for estimating water consumption per unit energy produced is Maheu [96], which offers global average water consumption coefficients for each fuel type. These coefficients are somewhat higher than the values used in GCAM, as they do not distinguish between seawater and freshwater. The values read into GCAM are therefore the Maheu [96] estimates less the fraction assumed to be seawater. This fraction is assumed to be 95% in the Middle East, and 43% in all other regions; the latter value is from a USA-based estimate in Kenny et al. [97]. Water withdrawals are estimated as water consumption multiplied by an exogenous withdrawal to consumption ratio of 3.3, which comes from a 1995 USA assessment [98].

Food and Agriculture Organization (FAO) Aquastat [99] withdrawals are assigned to a municipal water sector in each region, growing with population, GDP, and moderated by technical change. Prices are from International Benchmarking Network (IBNET) [100]. The demand projection functional form accounts for future price increases. Consumption is also modeled, with the distinction from withdrawals based on an overall municipal supply efficiency from Shiklomanov [101]. Withdrawals indicate total input to the supply system, while consumption is the volume not returned. The future withdrawal/consumption ratio is static, so both scale with demand. This represents growing municipal water needs. Adaptation measures can explore altering intensity. See Hejazi et al.[89,102] for details. The approach combines data on total use and supply efficiency to separately model withdrawn and consumed municipal water.

Supplementary Note 10: GCAM's fertilizer module

GCAM includes a nitrogen (N) fertilizer module with regional production technologies and crop/AEZ consumption. Production and consumption by country use FAO ResourceSTAT data, with uniform

downward adjustment of production so global totals match consumption excluding non-agricultural uses. Production shares by technology are from International Energy Agency (IEA), which also provides regional energy intensities. Consumption is downscaled to crops via International Fertilizer Association (IFA)/FAO data, then to AEZ based on crop production and USDA details for the USA. Non-fuel costs are calibrated to market prices in base years. Input-output coefficients (kgN per kg crop) are constant, so future demand scales with yield improvements. FAO provides historical production including non-agricultural uses, exceeding consumption by 5-10%. GCAM includes only agricultural fertilizer, so global production is uniformly adjusted downward to match consumption. With significant base year trade, GCAM includes exogenous fertilizer trade. Exporting regions have additional fixed final demand, while importers have extra production without energy inputs. This captures fertilizer production technologies, energy use, consumption by crop and location, and trade flows to represent agricultural nitrogen demands.

Supplementary Note 11: Model parameterization

In this section, some underlying parameters/modelling assumptions are presented. Unless cited otherwise, all information is obtained from Ref. [103], and additional information not discussed or presented here can be obtained from the same source.

Supplementary Table 8 Capital cost assumptions for the electric power sector (2010 $ per kilowatt (kW))

Electricity Generation Technology	Overnight Capital Costs (2010 $ per kW)		
	2020	2030	2050
Biomass (conventional)	3,951	3,818	3,702
Biomass (Integrated Gasification Combined Cycle)	5,745	5,180	4,819
Biomass (conventional with Carbon Capture and Storage)	7,317	6,568	6,168
Biomass (Integrated Gasification Combined Cycle with Carbon Capture and Storage)	8,337	7,298	6,720
Coal (conventional pulverized)	2,337	2,242	2,196
Coal (Integrated Gasification Combined Cycle)	3,060	2,854	2,769
Coal (conventional pulverized with Carbon Capture and Storage)	5,503	4,925	4,619
Coal (Integrated Gasification Combined Cycle with Carbon Capture and Storage)	4,020	3,607	3,448
Gas (Combined Cycle)	859	824	807
Gas (steam/Combustion Turbine)	911	875	857
Gas (Combined Cycle with Carbon Capture and Storage)	1,864	1,677	1,605
Refined liquids (steam/Combustion Turbine)	742	717	694
Refined liquids (Combined Cycle)	1,036	1,004	972
Refined liquids (Combined Cycle with Carbon Capture and Storage)	2,356	2,079	1,937

Generation II Light Water Reactor (Nuclear)	5,500	5,500	5,500
Generation III (Nuclear)	4,400	4,044	3,901
Concentrated Solar Power	3,415	3,077	2,946
Concentrated Solar Power with storage	7,430	6,329	5,771
Photovoltaic	1,856	1,534	1,514
Photovoltaic with storage	4,212	3,799	3,534
Wind	1,662	1,526	1,481
Wind with storage	5,555	5,006	4,661
Rooftop Photovoltaic	4,499	4,057	3,776
Geothermal	4,348	4,199	4,073

This table presents only the overnight capital costs. A fixed charge rate of 13% is assumed to amortize capital costs over the capital lifetime of a power plant.

Supplementary Table 9 Physical lifetime assumptions for technologies in the electric power sector

Technology	Lifetime (years)
Biomass (conventional)	60
Biomass (Integrated Gasification Combined Cycle)	60
Biomass (conventional with Carbon Capture and Storage)	60
Biomass (Integrated Gasification Combined Cycle with Carbon Capture and Storage)	60
Coal (conventional pulverized)	60
Coal (Integrated Gasification Combined Cycle)	60
Coal (conventional pulverized with Carbon Capture and Storage)	60
Coal (Integrated Gasification Combined Cycle with Carbon Capture and Storage)	60
Gas (Combined Cycle)	45
Gas (steam/Combustion Turbine)	45
Gas (Combined Cycle with Carbon Capture and Storage)	45
Refined liquids (steam/Combustion Turbine)	45
Refined liquids (Combined Cycle)	45
Refined liquids (Combined Cycle with Carbon Capture and Storage)	45
Generation II Light Water Reactor (Nuclear)	60
Generation III (Nuclear)	60
Wind	30
Wind with storage	30
Photovoltaic	30
Photovoltaic with storage	30
Concentrated Solar Power	30
Concentrated Solar Power with storage	30
Geothermal	30

Supplementary Table 10 Fraction of CO_2 captured by transformation technologies

Supply sector	Subsector	Technology	1971	2100
Refining	Coal to liquids	Coal to liquids ccs level 1	0.818	0.818
Refining	Coal to liquids	Coal to liquids ccs level 2	0.9	0.9
Refining	Biomass liquids	Cellulosic ethanol ccs level 1	0.26	0.26
Refining	Biomass liquids	Cellulosic ethanol ccs level 2	0.9	0.9
Refining	Biomass liquids	Ft biofuels ccs level 1	0.818	0.818
Refining	Biomass liquids	Ft biofuels ccs level 2	0.9	0.9

Ft: Fischer-Tropsch; ccs: carbon capture and storage

Supplementary Table 11 Primary energy transformation technologies default cost assumptions (1975$ per GJ)

Supply sector	Technology	1971	2010	2100	Improvement max	Improvement rate
Gas processing	Natural gas	0.2	0.2	0.2		
Gas processing	Biomass gasification	7.030087	7.030087		0.7	0.03
Gas processing	Coal gasification	5.285779	5.285779		0.7	0.03
Nuclear fuel generation II	Enriched uranium	0.124464	0.124464	0.124464		
Nuclear fuel generation III	Enriched uranium	0.124464	0.124464	0.124464		
Refining	Oil refining	0.84	0.84	0.84		
Refining	Coal to liquids	5.294118	5.294118		0.7	0.03
Refining	Coal to liquids ccs level 1	5.980615	5.980615		0.6	0.05
Refining	Coal to liquids ccs level 2	6.467671	6.467671		0.6	0.05
Refining	Gas to liquids	3.970588	3.970588		0.7	0.03
Refining	Cellulosic ethanol	4.74	4.74		0.7	0.03
Refining	Cellulosic ethanol ccs level 1	4.991818	4.991818		0.6	0.05
Refining	Cellulosic ethanol ccs level 2	6.850562	6.850562		0.6	0.05
Refining	Fischer Tropsch (Ft) biofuels	7.802308	7.802308		0.7	0.03
Refining	Ft biofuels ccs level 1	8.516923	8.516923		0.6	0.05
Refining	Ft biofuels ccs level 2	8.97527	8.97527		0.6	0.05
Refining	Corn ethanol	2.38	2.38	2.38		
Refining	Sugar cane ethanol	2	2	2		
Refining	Biodiesel	1.88	1.88	1.88		

Ft: Fischer-Tropsch; ccs: carbon capture and storage

Supplementary Table 12 Electricity technology capacity factors

Supply sector	Subsector	Technology	1971	2100
Electricity	Coal	Coal (Conventional Pulverized Coal	0.85	0.85
Electricity	Coal	Coal (Conventional Pulverized Coal with CCS)	0.8	0.8
Electricity	Coal	Coal (Integrated Gasification Combined Cycle)	0.8	0.8
Electricity	Coal	Coal (Integrated Gasification Combined Cycle with CCS)	0.8	0.8
Electricity	Gas	Gas (Steam Cycle/Turbine)	0.8	0.8
Electricity	Gas	Gas (Combined Cycle)	0.85	0.85

Electricity	Gas	Gas (Combined Cycle with CCS)	0.8	0.8
Electricity	Refined liquids	Refined liquids (Steam Cycle/Turbine)	0.8	0.8
Electricity	Refined liquids	Refined liquids (Combined Cycle)	0.85	0.85
Electricity	Refined liquids	Refined liquids (Combined Cycle with CCS)	0.8	0.8
Electricity	Biomass	Biomass (conventional)	0.85	0.85
Electricity	Biomass	Biomass (conventional with CCS)	0.85	0.85
Electricity	Biomass	Biomass ((Integrated Gasification Combined Cycle)	0.8	0.8
Electricity	Biomass	Biomass ((Integrated Gasification Combined Cycle with CCS)	0.8	0.8
Electricity	Nuclear	Generation II Light Water Reactor	0.9	0.9
Electricity	Nuclear	Generation III	0.9	0.9
Electricity	Wind	Wind	0.37	0.37
Electricity	Wind	Wind with storage	0.37	0.37
Electricity	Solar	Photovoltaic (PV)	0.2	0.2
Electricity	Solar	PV with storage	0.2	0.2
Electricity	Solar	Concentrated solar power (CSP)	0.25	0.25
Electricity	Solar	CSP with storage	0.5	0.5
Electricity	Geothermal	Geothermal	0.9	0.9
Electricity	Rooftop Photovoltaic (PV)	Rooftop Photovoltaic (PV)	0.17	0.17

Supplementary Table 13 *Electricity technology capture fractions (portion of CO_2 emissions that are captured)*

Supply sector	Subsector	Technology	1971	2020	2100
Electricity	Coal	Coal (Conventional Pulverized Coal with CCS)	0.85	0.85	0.95
Electricity	Coal	Coal (Integrated Gasification Combined Cycle with CCS)	0.85	0.85	0.95
Electricity	Gas	Gas (Combined Cycle with CCS)	0.85	0.85	0.95
Electricity	Refined liquids	Refined liquids (Combined Cycle with CCS)	0.85	0.85	0.95
Electricity	Biomass	Biomass (conventional with CCS)	0.85	0.85	0.95
Electricity	Biomass	Biomass ((Integrated Gasification Combined Cycle with CCS)	0.85	0.85	0.95

Supplementary Table 14 Electricity technology retirement parameters

Subsector	Technology	Year	Lifetime	Half life	Steepness
Coal	Coal (Conventional Pulverized Coal)	Final-calibration-year	60	30	0.1
Gas	Gas (Steam Cycle/Turbine)	Final-calibration-year	45	22.5	0.1
Gas	Gas (Combined Cycle)	Final-calibration-year	45	22.5	0.1
Refined liquids	Refined liquids (Steam Cycle/Turbine)	Final-calibration-year	45	22.5	0.1
Biomass	Biomass (conventional)	Final-calibration-year	60	30	0.1
Nuclear	Generation II Light Water Reactor	Final-historical-year	60	30	0.1
Wind	Wind	Final-calibration-year	30	-	-
Solar	Photovoltaic	Final-calibration-year	30	-	-
Solar	Concentrated solar power	Final-calibration-year	30	-	-
Geothermal	Geothermal	Final-calibration-year	30	-	-
Coal	Coal (Conventional Pulverized Coal)	Initial-future-year	60	-	-
Coal	Coal (Integrated Gasification Combined Cycle)	Initial-future-year	60	-	-
Coal	Coal (Integrated Gasification Combined Cycle with Carbon Capture and Storage)	Initial-future-year	60	-	-
Gas	Gas (Combined Cycle with Carbon Capture and Storage)	Initial-future-year	45	-	-
Refined liquids	Refined liquids (Combined Cycle)	Initial-future-year	45	-	-

Refined liquids	Refined liquids (Combined Cycle with Carbon Capture and Storage)	Initial-future-year	45	-	-
Biomass	Biomass (conventional with CCS)	Initial-future-year	60	-	-
Biomass	Biomass (Integrated Gasification Combined Cycle)	Initial-future-year	60	-	-
Biomass	Biomass (Integrated Gasification Combined Cycle with Carbon Capture and Storage)	Initial-future-year	60	-	-
Nuclear	Generation III	Initial-nonhistorical-year	60	-	-
Wind	Wind with storage	Initial-future-year	30	-	-
Solar	Photovoltaic with storage	Initial-future-year	30	-	-
Solar	Concentrated solar power with storage	Initial-future-year	30	-	-
Wind	Wind offshore	Final-calibration-year	25	-	-
Wind	Wind offshore	Initial-future-year	25	-	-

Note: lifetime: maximum lifetime of cohort. If no retirement function is used; the entire cohort is retired in this number of years.

half life: number of years at which 50% of the cohort is retired; using the s-curve-shutdown-decider retirement function.

steepness: shape parameter used by the s-curve-shutdown-decider retirement function.

Supplementary Table 15 Industrial energy use default efficiencies

Technology	Energy input	Secondary output	1971	2020	2050	2080	2100
Biomass	Delivered biomass		0.746423	0.797	0.81	0.823	0.828
Biomass cogeneration	Delivered biomass	Electricity	0.515677	0.56	0.577	0.595	0.604
Coal	Delivered coal		0.80808	0.891	0.909	0.926	0.936
Coal cogeneration	Delivered coal	Electricity	0.582005	0.629	0.644	0.661	0.67
District heat	District heat		1	1	1	1	1
Electricity	Electricity (industry)		0.934197	1.015	1.046	1.078	1.094
Gas	Wholesale gas		0.82583	0.898	0.926	0.955	0.969

Gas cogeneration	Wholesale gas	Electricity	0.563321	0.612	0.63	0.649	0.659
Hydrogen	Hydrogen enduse		1	1	1.03	1.062	1.078
Hydrogen cogeneration	Hydrogen enduse	Electricity	0.457	0.457	0.471	0.485	0.492
Refined liquids	Refined liquids industrial		0.917381	1.001	1.033	1.062	1.077
Refined liquids cogeneration	Refined liquids industrial	Electricity	0.565189	0.614	0.632	0.652	0.662
Coal	Delivered coal		1	1	1	1	1
Gas	Wholesale gas		1	1	1	1	1
Refined liquids	Refined liquids industrial		1	1	1	1	1

Supplementary Table 16 Carbon storage resource supply curve points (2005$ per tCO_2)

Resource	Subresource	Grade	Fraction	Cost
Onshore carbon-storage	Onshore carbon-storage	Grade 1	0	0
Onshore carbon-storage	Onshore carbon-storage	Grade 2	0.005	0.1
Onshore carbon-storage	Onshore carbon-storage	Grade 3	0.1	5
Onshore carbon-storage	Onshore carbon-storage	Grade 4	0.6	10
Onshore carbon-storage	Onshore carbon-storage	Grade 5	0.295	75
Onshore carbon-storage	Onshore carbon-storage	Grade 6	0	3500

Supplementary Table 17 Calibration values for the CO_2 removal sector (million tonnes of carbon (MtC))

GCAM region	sector	year	value
Central Asia	CO_2 removal	2015	429.8247
China	CO_2 removal	2015	2116.542
India	CO_2 removal	2015	87.41423
Indonesia	CO_2 removal	2015	16.37849
Japan	CO_2 removal	2015	66.31417
Middle East	CO_2 removal	2015	334.5848
Pakistan	CO_2 removal	2015	7.458686
South Asia	CO_2 removal	2015	8.830837
South Korea	CO_2 removal	2015	0
Southeast Asia	CO_2 removal	2015	165.692
Taiwan (China)	CO_2 removal	2015	0.307808

Supplementary Table 18 CO_2 capture rates for direct air capture (DAC) and process heat DAC technology

Supply sector	Subsector	Technology	1971	2100
Process heat DAC	Gas ccs	Gas ccs	0.95	0.95
CO_2 removal	DAC	High temperature DAC (natural gas)	1	1
CO_2 removal	DAC	High temperature DAC (electricity)	1	1
CO_2 removal	DAC	Low temperature DAC (heat pump)	1	1

DAC: direct air capture; ccs: carbon capture and storage

Supplementary Table 19 Parametrizations for DACCS Technologies [1]

Technology	Scenario	Natural gas ($GtCO_2$)		Electricity ($GtCO_2$)		Non-energy cost (2015 $$tCO_2$)		Water cubic metres per tonne of CO_2 (m^3 per tCO_2)	
		2020	2030	2020	2030	2020	2030	2020	2030
High temperature direct air capture and carbon storage (DACCS) (natural gas)	Shared Socio-economic pathway (SSP) 2-middle of the road		5.3		1.3		185	4.7	
High temperature DACCS (fully electric)	SSP2-middle of the road	_			5		186	4.7	
Low temperature DACCS (electric heat pump)	SSP2-middle of the road	_			2.5		235	_	

Values are assumed to remain constant after 2030

Supplementary Table 20 Enhanced Weathering Cost Adder [22,77]

Cost type	Upper bound	Best estimate	Units
Investment	$14	$6	$ per t rock
Operating and maintenance (O&M)	$59	$26	$ per t rock
Total non-cost fuel	$73	$31	$ per t rock
	$242	$104	$ per t CO_2
Difference between upper bound + best estimate (2020 cost adder for GCAM assumption; declines to zero by 2050)	$138		$ per t CO_2

Supplementary Table 21 Electrical energy inputs for enhanced weathering [22,77]

Best estimate	0.66	Gigajoule (GJ) per tCO_2
Lower bound	0.23	GJ per tCO_2
Upper bound	2.03	GJ per tCO_2

Supplementary Table 22 Electrical Energy Input Derivation for DORCS paired with reverse osmosis desalination [22]

Row	Value	Units	Source
1	0.075	Kilowatt hour per kilogram of CO_2 (kWh kg^{-1} CO_2) captured (capture energy only; standalone capture energy equal to this value)	Ref. [52]
2	277.77	KWh per GJ	Unit conversion
3	3.666667	$kgCO_2$ per kgC	Unit conversion
4	9.89E-04	Gigajoule (GJ) electricity per kgC captured	Calculation [1]/[2]*[3]
5	13.1	Cubic metres (m^3) ocean water per kg captured CO_2	ref
6	48.03	m^3 ocean water per kg captured C	Calculation [5]*[1]
7	2.5	m^3 seawater processed per m^3 desalinated water produced	GCAM assumption
8	5.15E-05	GJ elec per m^3 desalinated water (CO_2 capture only)	Calculation [4]/[6]*[7]
9	2.20E-02 - 6.30E-03	GJ elec per m^3 desalinated water (desalination)	GCAM assumption

kgC: kilogram of carbon. m^3: cubic metres.

Supplementary Table 23 Non-fuel costs of DORCS [22]

Ocean capture scenario	Capital Expenditure (CapEx) (\$ $kg^{-1}CO_2$)	Non-fuel Operational Expenditure (Opex) (\$ kg^{-1} CO_2)			Total (\$ kg^{-1} CO_2)	GDP deflator from 2020 to 1975	1975 (\$$kg^{-1}$ C)
		O&M	Labor, tax, insurance	Replacements			
Co-located	0.18	0.05	0.06	0.18	0.47	3.79	0.45
Stand-alone	1.07	0.18	0.27	0.18	1.7		1.64

GDP: gross domestic product. \$$kg^{-1}$ C: Dollars per kilogram of carbon. \$$kg^{-1}CO_2$: Dollars per kilogram of carbon dioxide.

Supplementary Table 24 Non-energy cost assumptions for DORCS paired with reverse osmosis desalination [22]

Row	Description	Value	Units	Source
1	non-fuel costs of CO_2 capture for co-located DORCS	0.45	1975\$ per kgC	*
2	m^3 ocean water per kg captured C	48.03	m^3 per kgC	[52]
3	m^3 seawater processed per m^3 desalinated water produced	2.5	unitless	GCAM assumption
4	Non-energy cost adder for CO_2 capture	0.023	\$1975 per m^3 desalinated water	Calculation [1]/[2]*[3]
5	Non-fuel cost of desalinated water (reverse osmosis technology)	0.38	\$1975 per m^3 desalinated water	GCAM assumption
6	Total non-fuel cost of DORCS + reverse osmosis	0.40	\$1975per m^3 desalinated water	Calculation [4]+[5]

*Based on Supplementary Table 2 from Ref. 22. kgC: kilogram of carbon. m^3: cubic metres.

Supplementary Table 25 GCAM Assumptions for Direct Ocean Removal [22]

Type	Electricity input (GJ per tCO_2)	Non-fuel cost (2020$ per tCO_2)
Stand-alone	16.5	1700
Co-located with desalination (carbon capture only)	0.26	470

Supplementary Table 26 Biochar supply and demand assumption in GCAM [22,29]

Metric	Value	Unit	Source
Non-energy cost	45.93	2007 USD per ton of feedstock	[61]
Biomass input	3.65	Tons of switchgrass per ton of biochar	
Natural gas input	211.19	Megajoule (MJ) per dry ton of biochar	
Syngas co-product	20095	MJ per dry ton of biochar	
Net syngas co-product	19884	MJ per dry ton of biochar	
Application rate	10<br>20	Tons of biochar per hectare	[30,31]
Yield improvements	12 (tropical irrigated)<br>19 (tropical rainfed)<br>10 (temperate irrigated)<br>15 (temperate rainfed)	Percentage	
Carbon sequestered	70	Percentage	[104]

Application rates of 10/20/100 tons per hectare and sequestration rates of 50/70/90% are considered in this study

Supplementary Table 27 Average vegetation carbon density by SAGE biome type [105]

GCAM category	SAGE category	C density (Megagrams of Carbon per Hectare (MgC per ha))
AllForestLand	Tropical Evergreen Forest/Woodland	200
AllForestLand	Tropical Deciduous Forest/Woodland	140
AllForestLand	Temperate Broadleaf Evergreen Forest/Woodland	154
AllForestLand	Temperate Needleaf Evergreen Forest/Woodland	160
AllForestLand	Temperate Deciduous Forest/Woodland	135
AllForestLand	Boreal Evergreen Forest/Woodland	90
AllForestLand	Boreal Deciduous Forest/Woodland	90
AllForestLand	Evergreen/Deciduous Mixed Forest/Woodland	103 (Agro-Ecological Zone (AEZs) 1-12)
AllForestLand	Evergreen/Deciduous Mixed Forest/Woodland	50 (AEZs 13-18)
GrassLand	Savanna	25
GrassLand	Grassland/Steppe	4-10*[a]
ShrubLand	Dense Shrubland	55
ShrubLand	Open Shrubland	27
Tundra	Tundra	9
RockIceDesert	Desert	1
RockIceDesert	Polar Desert/Rock/Ice	0

A: Grassland carbon contents are assumed to increase with moisture, such that AEZs 1,7, and 13 have 4 MgC per ha, and AEZs 6, 12, and 18 have 10 MgC per ha

Supplementary Table 28 Average soil carbon density by SAGE biome type [105]

GCAM category	SAGE category	C density (MgC per ha)
AllForestLand	Tropical Evergreen Forest/Woodland	98
AllForestLand	Tropical Deciduous Forest/Woodland	98
AllForestLand	Temperate Broadleaf Evergreen Forest/Woodland	71
AllForestLand	Temperate Needleaf Evergreen Forest/Woodland	134
AllForestLand	Temperate Deciduous Forest/Woodland	134
AllForestLand	Boreal Evergreen Forest/Woodland	206
AllForestLand	Boreal Deciduous Forest/Woodland	206
AllForestLand	Evergreen/Deciduous Mixed Forest/Woodland	111 (AEZs 1-12)
AllForestLand	Evergreen/Deciduous Mixed Forest/Woodland	206 (AEZs 13-18)
GrassLand	Savanna	95
GrassLand	Grassland/Steppe	60-185*
ShrubLand	Dense Shrubland	69
ShrubLand	Open Shrubland	69
Tundra	Tundra	100-300*
RockIceDesert	Desert	38
RockIceDesert	Polar Desert/Rock/Ice	85

Supplementary Method 1: Global Change Assessment Model (GCAM)

The Global Change Analysis Model (GCAM), developed by the Joint Global Change Research Institute (JGCRI), serves as a pivotal tool in the exploration of the consequences and responses to global changes, with a particular focus on climate change. This model is an integrated assessment tool designed to provide comprehensive insights into the multifaceted impacts of climate change across various regions of the world and diverse sectors of the global economy. The primary objective of GCAM is to facilitate a profound understanding of the potential ramifications of climate mitigation actions, thereby aiding in the formulation of informed and effective policies and international agreements aimed at limiting greenhouse gas emissions.

"GCAM includes representations of five systems: economy, energy, agriculture and land-use, water and climate in 32 geopolitical regions across the globe and the associated land allocation, water use and agriculture production across 384 land subregions and 235 water basins. GCAM operates in 5-year time-steps from 2015 (calibration year) to 2100 by solving for the equilibrium prices and quantities of various

energy, agricultural, water, land-use and GHG markets in each time period and in each region. GCAM is a dynamic recursive model. Hence, solutions for each modelling period only depend on conditions in the last modelling period. Outcomes of GCAM are driven by exogenous assumptions about population growth, labour participation rates and labour productivity in the 32 geopolitical regions, along with representations of resources, technologies and policy. GCAM tracks emissions of 24 gases, including GHGs, short-lived species and ozone precursors, endogenously based on the resulting energy, agriculture and land-use systems as discussed in the following subsections. Selected underlying modelling equations are also presented as follows [36,103]: The model reduces greenhouse gases by placing a price on GHG emissions. This price then filters down through all the systems in the model and alters production and demand. For example, a price on carbon would put a cost on emitting fossil fuels. This cost would then influence the cost of producing electricity from fossil-fired power plants that emit CO_2, which would then influence their relative cost compared to other electricity-generating technologies and increase the price of electricity. The increased price of electricity would then make its way to consumers who use electricity, potentially decreasing its competitiveness relative to other fuels [36,103].

When a policy is initiated, such as a target to achieve net-zero emissions in 2050, the carbon price begins to increase and follows the steps described above to achieve the emission target across every modeling period.

Supplementary Method 2: Demand for energy services in GCAM

In GCAM, the growth of the energy system is driven fundamentally by the demand for energy services from end-use sectors such as industry, transportation, and buildings. The GCAM models the link between this demand side and the supply side that meets energy service needs. The demand for energy services in the end-use sectors can be calculated using Supplementary Equation 8 [106].

$$D_t = D_{bs} \times \left(\frac{G_t}{G_{bs}}\right)^{\alpha} \times \left(\frac{E_t}{E_{bs}}\right)^{\beta} \times \frac{N_t}{N_{bs}} \tag{8}$$

Where;

D_t = Energy service demand in year t; D_{bs} = Energy service demand in reference year; E_t = Energy service price in year t; E_{bs} = Energy service price in reference year; N_t = Population in year t; N_{bs} = Population in reference year; G_t = GDP in year t; G_{bs}= GDP in reference year; α = Economic activity elasticity; and β = Price elasticity. The equation relates current year demand D_t to reference year demand D_{bs} adjusted for changes in population N, economic activity G, and energy prices E between the current year t and base year bs. The elasticities α and β determine demand sensitivity to changes in economic activity and prices.

Supplementary Method 3: Economic choice function in GCAM

In GCAM, various economic operations offer multiple methods for achieving the same final outcome. This includes options such as selecting among energy sources like coal or natural gas, technologies like solar panels or wind turbines, and transportation methods like trains or cars. Sometimes, the decision involves distributing a finite resource among different uses, such as allocating land between competing uses like farming or housing. GCAM makes choices by ranking alternatives on a single numeric scale. This is referred to as choice indicator p, which is usually cost or profitability, though other metrics can be employed theoretically. In situations where several factors affect the decision, these additional elements are translated into a cost penalty. This penalty is then added to the base cost, resulting in a comprehensive indicator that encompasses all pertinent aspects.

A "choice function" is a specialized function used to convert a set of indicators into corresponding market shares for different choice alternatives. This concept is vital because it acknowledges that the optimal choice, according to the indicators alone, doesn't always dominate the market. Various unmodeled elements such as personal preferences, local cost differences, and random occurrences lead to some market share being captured by options that are not the best according to their indicator values alone. GCAM's design allows

for dynamic specification of choice functions, tailored for each sector. These functions are programmed as classes adhering to the IDiscreteChoice interface. GCAM currently provides two such classes: Logit and Modified Logit. Both options belong to a category of choice functions that consider the suitability of an option based on two factors: one is directly related to the choice indicator (like cost), and the other encompasses elements not included in the model, treated as a random variable with a specific distribution. The choice of this distribution is crucial as it defines the type of discrete choice model being applied. This dual-component approach in choice functions helps in better reflecting the complexity and variability of market behaviors in the GCAM framework.

In the Logit model [107,108], the market share s_i of each choice alternative i, which is associated with a cost p_i, is calculated using Supplementary Equation 9.

$$s_i = \frac{\alpha_i \exp{(\beta p_i)}}{\sum_{j=1}^{N} \alpha_j \exp{(\beta p_j)}} \tag{9}$$

In this equation, the share weights, denoted as α_i, play a crucial role and serve two main purposes. Firstly, they are instrumental in the calibration process of the model to align with historical data. This calibration helps in integrating region-specific preferences into the model. These preferences can stem from various factors such as cultural inclinations, existing infrastructure, market entry barriers, or similar influences, and are captured within the share weight parameters. Secondly, share weights are used to manage the introduction and progression of new technologies in the market. This is achieved by initially assigning low share weights to new technologies when they first appear. Over time, these weights are incrementally increased towards a neutral value. This gradual adjustment reflects the realistic market adoption of new technologies, considering factors like development, acceptance, and increasing competitiveness over time. The βparameter in the Logit model, known as the logit coefficient, plays a significant role in determining the sensitivity of market share to differences in cost between various options. Essentially, it quantifies the extent to which a change in cost influences the distribution of market shares among different choices. To

illustrate this concept more clearly, we can consider the expression for the ratio of market shares of two different options, i and j

$$\frac{s_i}{s_j} = \frac{\alpha_i}{\alpha_j} \exp\left(\beta\left(p_i - p_j\right)\right) \tag{10}$$

Supplementary Equation 10 shows that, holding share weights constant, the market share ratio depends entirely on the cost difference. The role of the logit coefficient in this context is to determine the scale at which cost differences become significant in affecting market shares.

In the Modified Logit model [109], the formula for determining the market share s_i of each choice option i is calculated as (Supplementary Equation 11):

$$s_i = \frac{\alpha_i p_i^{\gamma}}{\sum_{j=1}^{N} \alpha_j p_j^{\gamma}} \tag{11}$$

Similar to the Logit model, α_i represent share weights in the Modified Logit model. There is also a new parameter, γ called the logit exponent that plays a similar role to the logit coefficient in the Logit model. The ratio of shares between options i and j is:

$$\frac{s_i}{s_j} = \frac{\alpha_i}{\alpha_j}\left(\frac{p_i}{p_j}\right)^{\gamma} \tag{12}$$

The Modified Logit model in GCAM employs the ratio of choice indicators in a way that parallels the use of the difference in indicators in the standard Logit model. In Supplementary Equation 12, the value of the logit exponent γ is critical in determining the extent to which the ratio of the choice indicators influences the market shares. A notable implication of this modeling approach is that the Modified Logit model can exhibit problematic behavior when any of the choice indicators approach zero. To address this issue, GCAM implements a practical solution by establishing a minimum threshold, or a 'floor', for the choice indicator values. Specifically, any alternative with a choice indicator $p_i < 0.001$ is treated as its $p_i = 0.001$. This floor ensures that the model can always compute a valid share value, preventing the calculation from becoming undefined or nonsensical due to very small indicator values.

Supplementary Method 4: Total technology cost in GCAM

The total cost of a technology is the sum of the cost of the technology, the cost of its inputs, and any greenhouse gas (GHG) value (Supplementary Equation 13):

$$C = t + \sum_{j=1}^{n} i_j + \sum_{k=1}^{m} g_k - \sum_{l=1}^{o} v_l \tag{13}$$

where C is the total cost, t \$ is the exogenously specified technology cost (capturing capital and O&M costs), i_j is the cost of input j (e.g., fuel), g_k is the GHG value of gas k, and v_l is the value of secondary output l. Costs vary by region, technology, and year.

Supplementary Method 5: Renewable resource supply in GCAM

The specific supply curve in each region for wind and solar energy is assigned three parameters, as detailed in the following equation (Supplementary Equation 14):

$$Q = maxSubResource * \frac{P^{CurveExponent}}{(MidPrice^{CurveExponent} + P^{CurveExponent})} \tag{14}$$

where Q refers to the quantity of electricity produced, P is the price, and the remaining parameters are exogenous, with the names in the Extensible Markup Language (XML) input files corresponding to the names in the equation above. $maxSubResource$ indicates the maximum quantity of renewable energy that can be produced at any price, $CurveExponent$ is a shape parameter, and $MidPrice$ indicates the price at which 50% of the maximum available resource is produced.

Supplementary References

1. Fuhrman, J. *et al.* The role of direct air capture and negative emissions technologies in the shared socioeconomic pathways towards +1.5 °C and +2 °C futures. *Environ. Res. Lett.* **16**, 114012 (2021).

2. Carton, W., Hougaard, I.-M., Markusson, N. & Lund, J. F. Is carbon removal delaying emission reductions? *WIREs Clim. Change* **14**, e826 (2023).

3. Fuhrman, J. *et al.* Food–energy–water implications of negative emissions technologies in a +1.5 °C future. *Nat. Clim. Change* **10**, 920–927 (2020).

4. Realmonte, G. *et al.* An inter-model assessment of the role of direct air capture in deep mitigation pathways. *Nat. Commun.* **10**, 3277 (2019).

5. Newman, J., Bonino, C. A. & Trainham, J. A. The Energy Future. *Annu. Rev. Chem. Biomol. Eng.* **9**, 153–174 (2018).

6. Are renewable heating options cost-competitive with fossil fuels in the residential sector? – Analysis. *IEA* https://www.iea.org/articles/are-renewable-heating-options-cost-competitive-with-fossil-fuels-in-the-residential-sector (2021).

7. Williams, J. H. *et al.* Carbon-Neutral Pathways for the United States. *AGU Adv.* **2**, e2020AV000284 (2021).

8. IPCC. *Climate Change 2022: Mitigation of Climate Change. Contribution of Working Group III to the Sixth Assessment Report of the Intergovernmental Panel on Climate Change*. ([P.R. Shukla, J. Skea, R. Slade, A. Al Khourdajie, R. van Diemen, D. McCollum, M. Pathak, S. Some, P. Vyas, R. Fradera, M. Belkacemi, A. Hasija, G. Lisboa, S. Luz, J. Malley, (eds.)]. Cambridge University Press, Cambridge, UK and New York, NY, USA, 2022).

9. Williams, M., Reay, D. & Smith, P. Avoiding emissions versus creating sinks—Effectiveness and attractiveness to climate finance. *Glob. Change Biol.* **29**, 2046–2049 (2023).

10. Förster, J. *et al.* Framework for Assessing the Feasibility of Carbon Dioxide Removal Options Within the National Context of Germany. *Front. Clim.* **4**, (2022).

11. Qiu, Y., Cohen, S. & Suh, S. Decarbonization scenarios of the U.S. Electricity system and their costs. *Appl. Energy* **325**, 119679 (2022).

12. Sepulveda, N. A., Jenkins, J. D., de Sisternes, F. J. & Lester, R. K. The Role of Firm Low-Carbon Electricity Resources in Deep Decarbonization of Power Generation. *Joule* **2**, 2403–2420 (2018).

13. Cole, W. J. *et al.* Quantifying the challenge of reaching a 100% renewable energy power system for the United States. *Joule* **5**, 1732–1748 (2021).

14. Janipour, Z., de Nooij, R., Scholten, P., Huijbregts, M. A. J. & de Coninck, H. What are sources of carbon lock-in in energy-intensive industry? A case study into Dutch chemicals production. *Energy Res. Soc. Sci.* **60**, 101320 (2020).

15. Zhao, C., Dong, K., Zheng, S., Fu, X. & Wang, K. Can China's aviation network development alleviate carbon lock-in? *Transp. Res. Part Transp. Environ.* **115**, 103578 (2023).

16. Seto, K. C. *et al.* Carbon Lock-In: Types, Causes, and Policy Implications. *Annu. Rev. Environ. Resour.* **41**, 425–452 (2016).

17. Chen, Y., Liu, K., Ni, L. & Chen, M. Impact of carbon lock-in on green economic efficiency: Evidence from Chinese provincial data. *Sci. Total Environ.* **892**, 164581 (2023).

18. Oh, D. & Lee, Y.-G. Productivity decomposition and economies of scale of Korean fossil-fuel power generation companies: 2001–2012. *Energy* **100**, 1–9 (2016).

19. IRENA. Renewable Power Remains Cost-Competitive amid Fossil Fuel Crisis. https://www.irena.org/news/pressreleases/2022/Jul/Renewable-Power-Remains-Cost-Competitive-amid-Fossil-Fuel-Crisis (2022).

20. Asayama, S. The Oxymoron of Carbon Dioxide Removal: Escaping Carbon Lock-In and yet Perpetuating the Fossil Status Quo? *Front. Clim.* **3**, (2021).

21. Baumgartner, T. CDReality: Is CDR a 'Moral Hazard'? *OpenAir Collective* https://openaircollective.cc/cdreality-is-cdr-a-moral-hazard/ (2023).

22. Fuhrman, J. *et al.* Diverse carbon dioxide removal approaches could reduce impacts on the energy–water–land system. *Nat. Clim. Change* (2023) doi:10.1038/s41558-023-01604-9.

23. Wang, W., Khanna, N., Lin, J. & Liu, X. Black carbon emissions and reduction potential in China: 2015–2050. *J. Environ. Manage.* **329**, 117087 (2023).

24. Karthik, V., Vijay Bhaskar, B., Ramachandran, S. & Gertler, A. W. Quantification of organic carbon and black carbon emissions, distribution, and carbon variation in diverse vegetative ecosystems across India. *Environ. Pollut.* **309**, 119790 (2022).

25. Liu, H. *et al.* Deployment of hydrogen in hard-to-abate transport sectors under limited carbon dioxide removal (CDR): Implications on global energy-land-water system. *Renew. Sustain. Energy Rev.* **184**, 113578 (2023).

26. Byers, E., Krey, V., Kriegler, E., Riahi, K. & Schaeffer, R. AR6 Scenario Explorer and Database hosted by IIASA. https://data.ece.iiasa.ac.at/ar6/#/workspaces/2123.

27. Dekker, M. M. *et al.* Identifying energy model fingerprints in mitigation scenarios. *Nat. Energy* **8**, 1395–1404 (2023).

28. Ou, Y. Decoding energy model variations. *Nat. Energy* **8**, 1309–1310 (2023).

29. Bergero, C., Wise, M., Lamers, P., Wang, Y. & Weber, M. Biochar as a carbon dioxide removal strategy in integrated long-run mitigation scenarios. *Environ. Res. Lett.* (2024) doi:10.1088/1748-9326/ad52ab.

30. Jeffery, S., Verheijen, F. G. A., Van Der Velde, M. & Bastos, A. C. A quantitative review of the effects of biochar application to soils on crop productivity using meta-analysis. *Agric. Ecosyst. Environ.* **144**, 175–187 (2011).

31. Ye, L. *et al.* Biochar effects on crop yields with and without fertilizer: A meta-analysis of field studies using separate controls. *Soil Use Manag.* **36**, 2–18 (2020).

32. Woolf, D. *et al.* Greenhouse Gas Inventory Model for Biochar Additions to Soil. *Environ. Sci. Technol.* **55**, 14795–14805 (2021).

33. Schmidt, H. *et al.* Pyrogenic carbon capture and storage. *GCB Bioenergy* **11**, 573–591 (2019).

34. Riahi, K. *et al.* Cost and attainability of meeting stringent climate targets without overshoot. *Nat. Clim. Change* **11**, 1063–1069 (2021).

35. Emmerling, J. *et al.* The role of the discount rate for emission pathways and negative emissions. *Environ. Res. Lett.* **14**, 104008 (2019).

36. Bond-Lamberty, B. *et al.* JGCRI/gcam-core: GCAM 5.4. (2021) doi:10.5281/zenodo.5093192.

37. Pradhan, S. *et al.* Effects of Direct Air Capture Technology Availability on Stranded Assets and Committed Emissions in the Power Sector. *Front. Clim.* **3**, 660787 (2021).

38. Speizer, S. *et al.* A zero-emissions global transportation sector: Advanced technologies and their energy and environmental implications. *ResearchSquare* (2023) doi:http://dx.doi.org/10.21203/rs.3.rs-2921936/v1.

39. Ou, Y. *et al.* State-by-state energy-water-land-health impacts of the US net-zero emissions goal. *Energy Clim. Change* **4**, 100117 (2023).

40. Zhao, M. *et al.* plutus: An R package to calculate electricity investments and stranded assets from the Global Change Analysis Model (GCAM). *J. Open Source Softw.* **6**, 3212 (2021).

41. Afrane, S. *et al.* Deployment of carbon removal technologies could reduce the rapid and potentially disruptive pace of decarbonization in South Africa's climate ambitions. *J. Clean. Prod.* **464**, 142753 (2024).

42. Fauvel, C. *et al.* Regional implications of carbon dioxide removal in meeting net zero targets for the United States. *Environ. Res. Lett.* **18**, 094019 (2023).

43. Wise, M. *et al.* Implications of Limiting CO2 Concentrations for Land Use and Energy. *Science* **324**, 1183–1186 (2009).

44. Wise, M. A., McJeon, H. C., Calvin, K. V., Clarke, L. E. & Kyle, P. Assessing the Interactions among U.S. Climate Policy, Biomass Energy, and Agricultural Trade. *Energy J.* **35**, 165–180 (2014).

45. Muratori, M. *et al.* Carbon capture and storage across fuels and sectors in energy system transformation pathways. *Int. J. Greenh. Gas Control* **57**, 34–41 (2017).

46. Fasihi, M., Efimova, O. & Breyer, C. Techno-economic assessment of CO2 direct air capture plants. *J. Clean. Prod.* **224**, 957–980 (2019).

47. Shiraki, H. & Sugiyama, M. Back to the basic: toward improvement of technoeconomic representation in integrated assessment models. *Clim. Change* **162**, 13–24 (2020).

48. Breyer, C., Fasihi, M. & Aghahosseini, A. Carbon dioxide direct air capture for effective climate change mitigation based on renewable electricity: a new type of energy system sector coupling. *Mitig. Adapt. Strateg. Glob. Change* **25**, 43–65 (2020).

49. DeVries, T. The oceanic anthropogenic CO2 sink: Storage, air-sea fluxes, and transports over the industrial era. *Glob. Biogeochem. Cycles* **28**, 631–647 (2014).

50. DeVries, T., Holzer, M. & Primeau, F. Recent increase in oceanic carbon uptake driven by weaker upper-ocean overturning. *Nature* **542**, 215–218 (2017).

51. Khatiwala, S. *et al.* Global ocean storage of anthropogenic carbon. *Biogeosciences* **10**, 2169–2191 (2013).

52. Digdaya, I. A. *et al.* A direct coupled electrochemical system for capture and conversion of CO2 from oceanwater. *Nat. Commun.* **11**, 4412 (2020).

53. Patterson, B. D. *et al.* Renewable CO2 recycling and synthetic fuel production in a marine environment. *Proc. Natl. Acad. Sci.* **116**, 12212–12219 (2019).

54. Eisaman, M. D. *et al.* Indirect ocean capture of atmospheric CO2: Part II. Understanding the cost of negative emissions. *Int. J. Greenh. Gas Control* **70**, 254–261 (2018).

55. Davies, P. A., Yuan, Q. & Richter, R. de. Desalination as a negative emissions technology. *Environ. Sci. Water Res. Technol.* **4**, 839–850 (2018).

56. Woolf, D., Amonette, J. E., Street-Perrott, F. A., Lehmann, J. & Joseph, S. Sustainable biochar to mitigate global climate change. *Nat. Commun.* **1**, 56 (2010).

57. Smith, P. Soil carbon sequestration and biochar as negative emission technologies. *Glob. Change Biol.* **22**, 1315–1324 (2016).

58. Jeffery, S. *et al.* Biochar boosts tropical but not temperate crop yields. *Environ. Res. Lett.* **12**, 053001 (2017).

59. Borchard, N. *et al.* Biochar, soil and land-use interactions that reduce nitrate leaching and N2O emissions: A meta-analysis. *Sci. Total Environ.* **651**, 2354–2364 (2019).

60. Razzaghi, F., Obour, P. B. & Arthur, E. Does biochar improve soil water retention? A systematic review and meta-analysis. *Geoderma* **361**, 114055 (2020).

61. Roberts, K. G., Gloy, B. A., Joseph, S., Scott, N. R. & Lehmann, J. Life Cycle Assessment of Biochar Systems: Estimating the Energetic, Economic, and Climate Change Potential. *Environ. Sci. Technol.* **44**, 827–833 (2010).

62. Woolf, D., Lehmann, J., Fisher, E. M. & Angenent, L. T. Biofuels from Pyrolysis in Perspective: Trade-offs between Energy Yields and Soil-Carbon Additions. *Environ. Sci. Technol.* **48**, 6492–6499 (2014).

63. Lehmann, J. *et al.* Biochar in climate change mitigation. *Nat. Geosci.* **14**, 883–892 (2021).

64. Woolf, D., Lehmann, J. & Lee, D. R. Optimal bioenergy power generation for climate change mitigation with or without carbon sequestration. *Nat. Commun.* **7**, 13160 (2016).

65. Hejazi, M. I. *et al.* Integrated assessment of global water scarcity over the 21st century under multiple climate change mitigation policies. *Hydrol. Earth Syst. Sci.* **18**, 2859–2883 (2014).

66. Calvin, K. *et al.* GCAM v5.1: representing the linkages between energy, water, land, climate, and economic systems. *Geosci. Model Dev.* **12**, 677–698 (2019).

67. Goddéris, Y. *et al.* Onset and ending of the late Palaeozoic ice age triggered by tectonically paced rock weathering. *Nat. Geosci.* **10**, 382–386 (2017).

68. Walker, J. C. G., Hays, P. B. & Kasting, J. F. A negative feedback mechanism for the long-term stabilization of Earth's surface temperature. *J. Geophys. Res. Oceans* **86**, 9776–9782 (1981).

69. Schuiling, R. D. & Krijgsman, P. Enhanced Weathering: An Effective and Cheap Tool to Sequester Co2. *Clim. Change* **74**, 349–354 (2006).

70. Renforth, P. The negative emission potential of alkaline materials. *Nat. Commun.* **10**, 1401 (2019).

71. Beerling, D. J. *et al.* Potential for large-scale CO2 removal via enhanced rock weathering with croplands. *Nature* **583**, 242–248 (2020).

72. Hartmann, J. *et al.* Enhanced chemical weathering as a geoengineering strategy to reduce atmospheric carbon dioxide, supply nutrients, and mitigate ocean acidification. *Rev. Geophys.* **51**, 113–149 (2013).

73. Renforth, P., Pogge von Strandmann, P. A. E. & Henderson, G. M. The dissolution of olivine added to soil: Implications for enhanced weathering. *Appl. Geochem.* **61**, 109–118 (2015).

74. Amann, T. *et al.* Enhanced Weathering and related element fluxes – a cropland mesocosm approach. *Biogeosciences* **17**, 103–119 (2020).

75. Kantzas, E. P. *et al.* Substantial carbon drawdown potential from enhanced rock weathering in the United Kingdom. *Nat. Geosci.* **15**, 382–389 (2022).

76. Goll, D. S. *et al.* Potential CO2 removal from enhanced weathering by ecosystem responses to powdered rock. *Nat. Geosci.* **14**, 545–549 (2021).

77. Strefler, J., Amann, T., Bauer, N., Kriegler, E. & Hartmann, J. Potential and costs of carbon dioxide removal by enhanced weathering of rocks. *Environ. Res. Lett.* **13**, 034010 (2018).

78. Keith, D. W., Holmes, G., Angelo, D. S. & Heidel, K. A Process for Capturing CO2 from the Atmosphere. *Joule* **2**, 1573–1594 (2018).

79. Beuttler, C., Charles, L. & Wurzbacher, J. The Role of Direct Air Capture in Mitigation of Anthropogenic Greenhouse Gas Emissions. *Front. Clim.* **1**, 10 (2019).

80. Beerling, D. J. *et al.* Farming with crops and rocks to address global climate, food and soil security. *Nat. Plants* **4**, 138–147 (2018).

81. Kantola, I. B., Masters, M. D., Beerling, D. J., Long, S. P. & DeLucia, E. H. Potential of global croplands and bioenergy crops for climate change mitigation through deployment for enhanced weathering. *Biol. Lett.* **13**, 20160714 (2017).

82. Taylor, L. L. *et al.* Enhanced weathering strategies for stabilizing climate and averting ocean acidification. *Nat. Clim. Change* **6**, 402–406 (2016).

83. Bertagni, M. B. & Porporato, A. The Carbon-Capture Efficiency of Natural Water Alkalinization: Implications For Enhanced weathering. *Sci. Total Environ.* **838**, 156524 (2022).

84. Smith, P. *et al.* Land-Management Options for Greenhouse Gas Removal and Their Impacts on Ecosystem Services and the Sustainable Development Goals. *Annu. Rev. Environ. Resour.* **44**, 255–286 (2019).

85. Wise, M., Calvin, K., Kyle, P., Luckow, P. & Edmonds, J. ECONOMIC AND PHYSICAL MODELING OF LAND USE IN GCAM 3.0 AND AN APPLICATION TO AGRICULTURAL PRODUCTIVITY, LAND, AND TERRESTRIAL CARBON. *Clim. Change Econ.* **05**, 1450003 (2014).

86. Zhao, X., Calvin, K. V. & Wise, M. A. THE CRITICAL ROLE OF CONVERSION COST AND COMPARATIVE ADVANTAGE IN MODELING AGRICULTURAL LAND USE CHANGE. *Clim. Change Econ.* **11**, 2050004 (2020).

87. Rohwer, J., Gerten, D. & Lucht, W. Development of functional irrigation types for improved global crop modelling. *PIK Rep.* 1–61 (2007).

88. Chaturvedi, V. *et al.* Climate mitigation policy implications for global irrigation water demand. *Mitig. Adapt. Strateg. Glob. Change* **20**, 389–407 (2015).

89. Hejazi, M. *et al.* Long-term global water projections using six socioeconomic scenarios in an integrated assessment modeling framework. *Technol. Forecast. Soc. Change* **81**, 205–226 (2014).

90. NETL. *Water Requirements for Existing and Emerging Thermoelectric Plant Technologies*. (2008).

91. Macknick, J., Newmark, R., Heath, G. & Hallet, K. *A Review of Operational Water Consumption and Withdrawal Factors for Electricity Generating Technologies*. (2011).

92. Davies, E. G. R., Kyle, P. & Edmonds, J. A. An integrated assessment of global and regional water demands for electricity generation to 2095. *Adv. Water Resour.* **52**, 296–313 (2013).

93. Kyle, P. *et al.* Influence of climate change mitigation technology on global demands of water for electricity generation. *Int. J. Greenh. Gas Control* **13**, 112–123 (2013).

94. Mekonnen, M. M. & Hoekstra, A. Y. A Global Assessment of the Water Footprint of Farm Animal Products. *Ecosystems* **15**, 401–415 (2012).

95. FAOSTAT. FAOSTAT Statistics Database. https://data.apps.fao.org/aquastat/?lang=en (2016).

96. Maheu, A. *Energy Choices and Their Impacts on Demand for Water Resources: An Assessment of Current and Projected Water Consumption in Global Energy Production*. https://unisfera.org/sn_uploads/0Energy_demand_on_water_Finalversion.pdf (2009).

97. Keeny, J. F. *et al. Estimated Use of Water in the United States in 2005*. (2009).

98. Solley, W. B., Pierce, R. R. & Perlman, H. A. *Estimated Use of Water in the United States in 1995*. https://pubs.usgs.gov/publication/cir1200 (1998) doi:10.3133/cir1200.

99. FAO. *AQUASTAT Main Database*. https://www.fao.org/faostat/en/#home (2016).

100. IBNET. *Benchmarking Database*. https://www.ib-net.org/ (2016).

101. Shiklomanov, I. A. *World Water Resources and Water Use: Present Assessment and Outlook for 2025*. (2000).

102. Hejazi, M., Edmonds, J., Chaturvedi, V., Davies, E. & Eom, J. Scenarios of global municipal water-use demand projections over the 21st century. *Hydrol. Sci. J.* **58**, 519–538 (2013).

103. Bond-Lamberty, B. *et al. JGCRI/Gcam-Core: GCAM 6.0*. https://zenodo.org/record/6619287 (2022) doi:10.5281/ZENODO.6619287.

104. Wang, J., Xiong, Z. & Kuzyakov, Y. Biochar stability in soil: meta-analysis of decomposition and priming effects. *GCB Bioenergy* **8**, 512–523 (2016).

105. Kyle, G. P. *et al. GCAM 3.0 Agriculture and Land Use: Data Sources and Methods*. PNNL-21025, 1036082 http://www.osti.gov/servlets/purl/1036082/ (2011) doi:10.2172/1036082.

106. Ma, X., Peng, T., Zhang, Y., Wang, L. & Pan, X. Accelerating carbon neutrality could help China's energy system align with below 1.5 °C. *J. Environ. Manage.* **337**, 117753 (2023).

107. Train, K. *Discrete Choice Methods with Simulation*. (Cambridge University Press, New York, 2003).

108. Mcfadden, D. L. *Conditional Logit Analysis of Qualitative Choice Behavior*. (Frontiers in Econometrics, Academic Press: New York, 1973).

109. Clarke, J. F. & Edmonds, J. A. Modelling energy technologies in a competitive market. *Energy Econ.* **15**, 123–129 (1993).
